# Thermal conductivity predictions with foundation atomistic models

**DOI:** 10.1038/s41467-026-76391-w

**Published:** 2026-09-05

**Authors:** Balázs Póta, Paramvir Ahlawat, Gábor Csányi, Michele Simoncelli

**Affiliations:** 1 Theory of Condensed Matter Group of the Cavendish Laboratory, University of Cambridge, Cambridge, UK; 2 Engineering Laboratory, University of Cambridge, Cambridge, UK; 3 Max Planck Institute for Polymer Research, Mainz, Germany; 4 Department of Applied Physics and Applied Mathematics, Columbia University, New York, USA

**Keywords:** Condensed-matter physics, Thermodynamics, Energy modelling, Computational science, Quantum simulation

## Abstract

Advances in machine learning have led to the development of foundation models for atomistic materials chemistry, enabling quantum-accurate descriptions of interatomic forces across chemically diverse compounds at reduced computational cost. Hitherto, the accuracy and utility of these models have been assessed relying on descriptors based on formation energies or idealized harmonic atomic vibrations. Yet, the rigorous and physically interpretable quantification of their capability to describe both realistic anharmonic atomic dynamics and technologically relevant observables remains a pressing problem. Here, we address this problem, leveraging the Wigner formulation of heat transport and the Grüneisen approach to thermal expansion to connect the atomic-physics awareness of foundation models to their utility in predicting experimentally observable thermomechanical properties, presenting standards and fine-tuning protocols needed to achieve first-principles accuracy. We apply our framework to a database of 103 solids with diverse compositions and structures, demonstrating that it overcomes the major bottlenecks of current methods for designing heat-management materials — high cost, limited transferability, or lack of physics awareness — and has the potential to discover materials for technologies ranging from thermal insulation to neuromorphic computing.

## Abstract (web summary)

Researchers show how foundation atomistic models can predict heat transport and thermal expansion, using physics-based benchmarks and fine-tuning to reach first-principles accuracy.

## Introduction

Over the past decades, intense research efforts have been dedicated to tackling the challenging task of fitting the Born-Oppenheimer potential energy surface (PES) as a function of atomic coordinates^1–10^. These resulted in the development of machine-learning potentials (MLPs), which allow us to reproduce atoms’ interaction energies and forces with nearly quantum accuracy and computational cost orders of magnitude lower than first-principles methods. These advances have enabled scientists to make progress in understanding how atomistic structure and composition influence macroscopic observables—an example is the thermal conductivity, a property determined by atomic structure and forces, and relevant for technologies ranging from energy harvesting^11^ to efficient neuromorphic computing^12^. Major drawbacks of methods based on MLPs are the significant work required to generate a first-principles database needed to train and validate MLPs, as well as the applicability limited to specific materials’ compositions or structural phases. Past works attempted to bypass these limitations by employing end-to-end machine-learning methods, which predict microscopic^13,14^ or macroscopic^15–17^ materials’ properties very efficiently, but without necessarily accounting for the fundamental physics that determines them. Advances over the last decade have shown that Message Passing Networks^18^, when combined with equivariant neural-network architectures^9^ and many-body messages^19^, can fit the PES in an accurate and data-efficient way for complex chemical compositions. This success can be understood in terms of a relation between body order and message-passing depth^20^: increasing the body order of the local messages and iterating message passing enriches the geometric information available to the network. These breakthroughs have enabled the development of foundation machine-learning potentials (fMLPs)^21–33^, which are trained across nearly all chemical elements and can be combined to describe almost arbitrary compounds. Recent works have assessed the accuracy of fMLPs in predicting, e.g., atoms’ interaction energies (thermodynamic stability^34^) or harmonic vibrations^35–37^. However, the rigorous and physically interpretable quantification of the fMLP’s capability to describe atomic-level features of the PES (i.e., their physics awareness) and their overall utility to determine macroscopic observables are pressing questions that are largely unexplored, due to the complexity of the relation between many-body interatomic forces and observables^38–40^.

Here we introduce a framework to rigorously address such a question, quantifying how the accuracy of fMLPs in describing interatomic forces and PES derivatives connects to their accuracy in predicting experimentally observable thermomechanical properties. We elucidate how fundamental insights into the physics awareness, accuracy, and overall utility of fMLPs can be derived from microscopic analysis of the linear-response solution of the Wigner Transport Equation (WTE) for heat conduction in crystals and glasses^38,39^, and the quasi-harmonic Grüneisen approach to describe thermal expansion^41–44^. We leverage these insights to define physically interpretable, robust, and stringent benchmark metrics that expose subtle artifacts or inaccuracies in the smoothness (second and third derivatives) of the PES encoded by fMLPs. As these derivatives expose even subtle discontinuities in the PES, compared to established energy-based metrics, our metrics provide information that is physically richer, more robust, and stricter for measuring both the utility of fMLPs and the physical accuracy (smoothness) of the encoded PES^45^. We showcase the capabilities of this combined fMLP-WTE-Grüneisen framework in state-of-the-art (SOTA) non-proprietary fMLPs trained on the Materials Project database^46^ (hereafter collectively referred to as mp-fMLPs)—M3GNet^21^, CHGNet^22^, MACE-MP-0^23^, SevenNet^24^, and ORB-v1-MPtraj^47^—assessing their performance over a database of first-principles reference data for 103 chemically and structurally diverse compounds^48,49^. We discuss how these metrics can be used to assess and improve the expressivity, training, and fine-tuning of fMLPs, enabling first-principles-level predictions of anharmonic vibrational properties and heat conductivity in solids with arbitrary compositions and structures. We conclude by showing how these developments enable the theory-driven design of heat-management materials with microscopic heat-transport physics that violates the semiclassical Boltzmann regime, which are critical for energy and information-management technologies.

## Results

### Atomic vibrations and thermomechanical observables

The recently developed WTE^38,39^ offers a comprehensive approach to predict from atomistic vibrational properties the thermal conductivity of anharmonic crystals^50^, disordered glasses^51^, as well as the intermediate case of anharmonic-and-disordered complex crystals^52^. To assess how the accuracy in the conductivity prediction is affected by the precision with which fMLPs describe atomic vibrations, it is sufficient to consider the directionally averaged trace of the conductivity tensor obtained from the WTE solution in the relaxation-time approximation^39^:

1 $$\kappa (T)=	 {\kappa }_{P}(T)+{\kappa }_{C}(T)=\frac{1}{{{{\mathcal{V}}}}{N}_{c}}\sum\limits_{{{{\bf{q}}}},s} \\ 	 \left[C{({{{\bf{q}}}})}_{s}\frac{{\left\Vert {{\mathbf{\sf{v}}}}{({{{\bf{q}}}})}_{s,s}\right\Vert }^{2}}{3}{[{\Gamma} {({{{\bf{q}}}})}_{s}]}^{-1}\right.\\ 	+\sum\limits_{{s}^{{\prime} }\ne s}\frac{C{({{{\bf{q}}}})}_{s}}{C{({{{\bf{q}}}})}_{s}+C{({{{\bf{q}}}})}_{{s}^{{\prime} }}}\frac{\omega {({{{\bf{q}}}})}_{s}+\omega {({{{\bf{q}}}})}_{{s}^{{\prime} }}}{2}\left(\frac{C{({{{\bf{q}}}})}_{s}}{\omega {({{{\bf{q}}}})}_{s}}+\frac{C{({{{\bf{q}}}})}_{{s}^{{\prime} }}}{\omega {({{{\bf{q}}}})}_{{s}^{{\prime} }}}\right)\\ 	 \times \left.\frac{{\left\Vert {{\mathbf{\sf{v}}}}{({{{\bf{q}}}})}_{s,{s}^{{\prime} }}\right\Vert }^{2}}{3}\frac{\frac{1}{2}\left[{\Gamma} {({{{\bf{q}}}})}_{s}+{\Gamma} {({{{\bf{q}}}})}_{{s}^{{\prime} }}\right]}{{[\omega {({{{\bf{q}}}})}_{{s}^{{\prime} }}-\omega {({{{\bf{q}}}})}_{s}]}^{2}+\frac{1}{4}{[{\Gamma} {({{{\bf{q}}}})}_{s}+{\Gamma} {({{{\bf{q}}}})}_{{s}^{{\prime} }}]}^{2}}\right].$$

Here, $C{({{{\bf{q}}}})}_{s}=\frac{{\hslash }^{2}{\omega }^{2}{({{{\bf{q}}}})}_{s}}{{k}_{{{{\rm{B}}}}}{T}^{2}}\bar{{\mathsf{N}}}{({{{\bf{q}}}})}_{s}\left[\bar{{\mathsf{N}}}{({{{\bf{q}}}})}_{s}+1\right]$ is the specific heat at temperature *T* of the vibration having: wavevector **q**; mode *s*; energy *ℏω*(**q**)_*s*_; population given by Bose-Einstein distribution $\bar{{\mathsf{N}}}{({{{\bf{q}}}})}_{s}={[\exp (\hslash \omega {({{{\bf{q}}}})}_{s}/{k}_{{{{\rm{B}}}}}T)-1]}^{-1}$; total linewidth Γ(**q**)_*s*_ = Γ_a_(**q**)_*s*_ + Γ_i_(**q**)_*s*_, where Γ_a_(**q**)_*s*_ depends on *T* and is determined by anharmonic third-order derivatives of the interatomic potential, while Γ_i_(**q**)_*s*_ is *T*-independent and regulated by concentration of isotopes (see Methods for details). ${{{\mathbf{\sf{v}}}}}{({{{\bf{q}}}})}_{s,{s}^{{\prime} }}$ is the velocity operator obtained from wavevector differentiation of the solid’s dynamical matrix^39^, its diagonal elements $s={s}^{{\prime} }$ are the phonon group velocities and its off-diagonal elements describe couplings between modes *s* and ${s}^{{\prime} }$ at the same **q**; *N*_*c*_ is the number of **q**-points sampling the Brillouin zone and ${{{\mathcal{V}}}}$ is the crystal’s unit-cell volume.

Eq. (1) shows in the first line that the total macroscopic conductivity is determined by two contributions: the particle-like conductivity (*κ*_*P*_), and the wave-like coherence conductivity (*κ*_*C*_). In such an equation, the term inside the square brackets is the single-phonon contribution to the total conductivity, ${{{\mathcal{K}}}}{({{{\bf{q}}}})}_{s}$, which, as we will show later, is useful for benchmarking fMLPs. The second line shows that *κ*_*P*_ originates from phonons that carry heat *C*(**q**)_*s*_ by propagating particle-like with velocity ${{\mathbf{\sf{v}}}}{({{{\bf{q}}}})}_{s,s}$ over lifetime ${[{\Gamma} {({{{\bf{q}}}})}_{s}]}^{-1}$; it can be shown^38^ that *κ*_*P*_ is equivalent to the conductivity described by the phonon Boltzmann Transport Equation (BTE). The term on the third and fourth lines, instead, shows that *κ*_*C*_ describes conduction through wave-like tunneling between pairs of phonons $s,{s}^{{\prime} }$ at wavevector **q**. It has been shown^38,39^ that in simple crystals particle-like propagation dominates over wave-like tunneling (*κ*_P_ ≫ *κ*_C_), in complex crystals both these mechanisms co-exist (*κ*_P_ ~ *κ*_C_), and in strongly disordered glasses tunneling dominates (*κ*_P_ ≪ *κ*_C_)^51^.

In addition to the conductivity, the thermal-expansion coefficient is also a property of technological interest, e.g., for aerospace applications^53^. In general, there is no simple correlation between thermal conductivity and thermal expansion coefficients—materials with similar thermal conductivity can exhibit different coefficients of thermal expansion, and vice-versa^54,55^. Therefore, it is important to investigate to which extent accuracy in predicting thermal conductivity correlates with accuracy in predicting thermal expansion. Thermal expansion, the change in volume ${{{\mathcal{V}}}}$ driven by a change in temperature *T* at constant pressure *P*, is related to the Grüneisen parameter, $\upgamma (T)={\left.-\frac{\partial \ln T}{\partial \ln {{{\mathcal{V}}}}}\right\vert }_{S}=\frac{\beta {B}_{T}}{C}$, where this derivative is taken at constant entropy *S*, and here *β* is the coefficient of volumetric thermal expansion, *B*_*T*_ is the isothermal bulk modulus, and $C=\frac{1}{{{{\mathcal{V}}}}{N}_{c}}{\sum }_{{{{\bf{q}}}},s}C{({{{\bf{q}}}})}_{s}$ is the specific heat at constant volume^41–44^. Within the quasi-harmonic approximation (QHA) one can resolve the Grüneisen parameter γ in terms of microscopic phonon contributions (γ(**q**)_*s*_),

2 $$\upgamma=\frac{1}{{{{\mathcal{V}}}}{N}_{c}}{\sum}_{{{{\bf{q}}}}s}\left[\upgamma {({{{\bf{q}}}})}_{s}\frac{C{({{{\bf{q}}}})}_{s}}{C}\right],\ \ {{{\rm{with}}}}\ \ \upgamma {({{{\bf{q}}}})}_{s}=-\frac{{{{\mathcal{V}}}}}{\omega {({{{\bf{q}}}})}_{s}}\frac{\partial \omega {({{{\bf{q}}}})}_{s}}{\partial {{{\mathcal{V}}}}}.$$

This microscopic decomposition shows that, within the QHA at a fixed reference volume, the microscopic phonon-resolved Grüneisen contributions γ(**q**)_*s*_ are constant over temperature, and the temperature dependence of the macroscopic Grüneisen parameter γ entirely derives from the phonon specific heat term $\frac{C{({{{\bf{q}}}})}_{s}}{C}$. In the following, we will refer to the temperature-dependent microscopic contribution to the macroscopic Grüneisen parameter as the quantity in the square brackets in Eq. (2), i.e., ${{{\mathcal{G}}}}{({{{\bf{q}}}})}_{s}=\upgamma {({{{\bf{q}}}})}_{s}\frac{C{({{{\bf{q}}}})}_{s}}{C}$.

In the Methods, we provide a quantitative explanation about the different information about the PES provided by constant-volume properties such as the conductivity, and volume-deforming properties such as the Grüneisen parameter. Specifically, within perturbation theory, the mode Grüneisen parameter is linearly related to the third-order derivatives of the PES^44^. In contrast, the particle-like propagation contribution to the thermal conductivity, which is dominant in many materials considered here (see Supplementary Fig. 1), is inversely proportional to the anharmonic linewidths, which depend on the square of the third-order PES derivatives^39^. Therefore, macroscopic conductivity and Grüneisen parameter have a very different dependence on microscopic interatomic forces; this implies that conductivity and Grüneisen parameter practically provide complementary information on how accuracy in predicting PES translates into accuracy in describing macroscopic thermomechanical properties.

### Vibrational and thermal properties from fMLPs

Since *κ*(*T*) and γ(*T*) are observables that can be resolved in terms of microscopic harmonic and anharmonic vibrational properties^39,56–58^, it is natural to use them to assess the precision of fMLPs in reproducing reference microscopic and macroscopic vibrational properties obtained from Density-Functional Theory (DFT) or from experiments. In particular, we rely on the DFT interatomic force data of refs. ^48,49^, which include 103 diverse binary compounds, involving 34 different chemical species, and rocksalt, zincblende, or wurtzite structures. In the following, we will refer to this dataset as phononDB-PBE to highlight that it was computed using the Perdew, Burke, and Ernzerhof (PBE) functional^59^ used also to generate the training data for the fMLPs^46^. Following the expressions detailed in the Methods, we use these data to calculate the harmonic (*ℏω*(**q**)_*s*_, ${{{\mathbf{\sf{v}}}}}{({{{\bf{q}}}})}_{s,{s}^{{\prime} }}$, and Γ_i_(**q**)_*s*_) and third-order anharmonic (Γ_a_(**q**)_*s*_) vibrational properties that determine *κ*(*T*) (Eq. (1)). To evaluate the Grüneisen parameter (2), we extend this dataset to include second-order force-constant calculations at expanded and contracted volumes, as detailed in the Methods and the Supplementary Information.

**Fig. 1:**
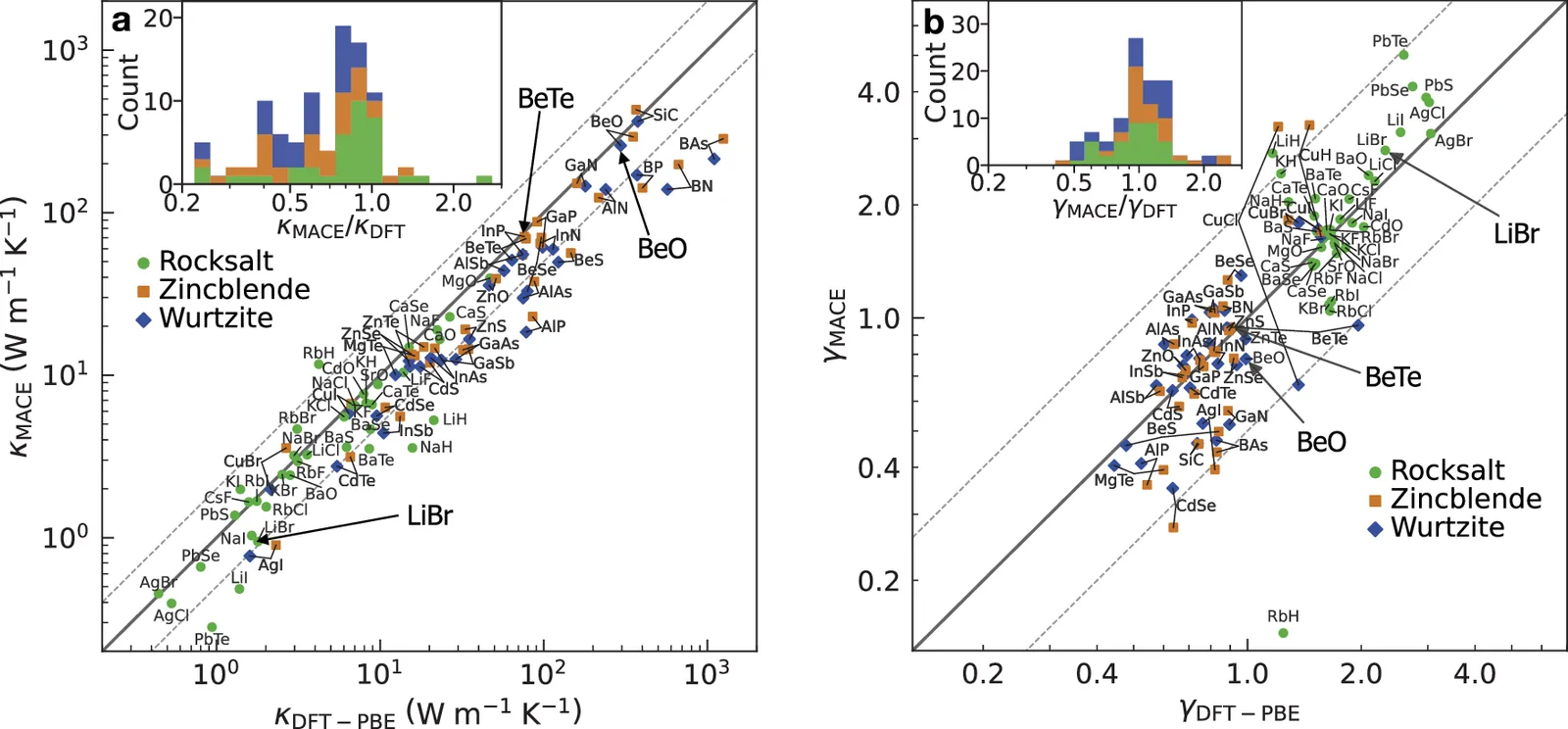
Conductivity and Grüneisen parameter at 300 K from DFT-PBE and MACE-MP-0. The evaluated 103 compounds are taken from the phononDB-PBE database, which have rocksalt (green), zincblende (orange), or wurtzite (blue) structures. The solid line indicates perfect agreement and the dashed lines discrepancies of a factor of 2. The arrows highlight materials discussed in detail later. Inset, distribution of relative deviations between the conductivities (**a**) and Grüneisen parameters (**b**) predicted by DFT-PBE and MACE-MP-0. The inset histograms show the distribution of the ratio between the MACE-MP-0 and the DFT results.

In Fig. 1a, b, we compare the conductivity and Grüneisen parameter, respectively, predicted from first principles (DFT-PBE) or from the mp-fMLP MACE-MP-0 large^23^ for the 103 materials in the phononDB-PBE dataset. We highlight that predictions from DFT or fMLPs are generally compatible within a factor of 2. Importantly, in about 20% of the compounds studied, the semiclassical particle-like BTE fails to fully describe heat transport, and it is crucial to employ the more general WTE to perform accurate predictions. These cases have very low conductivity (*κ*_TOT_ ≲ 2 W/mK), which receives significant contributions from both wave-like tunneling (described by the WTE but missing from the BTE) and particle-like propagation (described by both the WTE and BTE) heat-transport mechanisms, see Supplementary Fig. 1. We highlight how having accurate conductivity predictions does not necessarily imply having accuracy in predictions for the Grüneisen parameter, and vice-versa (e.g., in wurtzite BeO *κ* is predicted more accurately than γ, while in LiBr γ is more accurate than *κ*). We further observe that the distributions of *κ* and γ overlap for zincblende and wurtzite structures, while rocksalt structures generally exhibit lower *κ* and higher γ. Moreover, while fMLPs generally tend to systematically underestimate the conductivity, there is no such bias for the Grüneisen parameter. Overall, our findings highlight that the macroscopic *κ* and γ have different sensitivity to atomistic vibrational properties, and in the following, we show that they can provide complementary information on the accuracy with which fMLPs describe reference DFT data.

### Relating macroscopic and microscopic properties

**Fig. 2:**
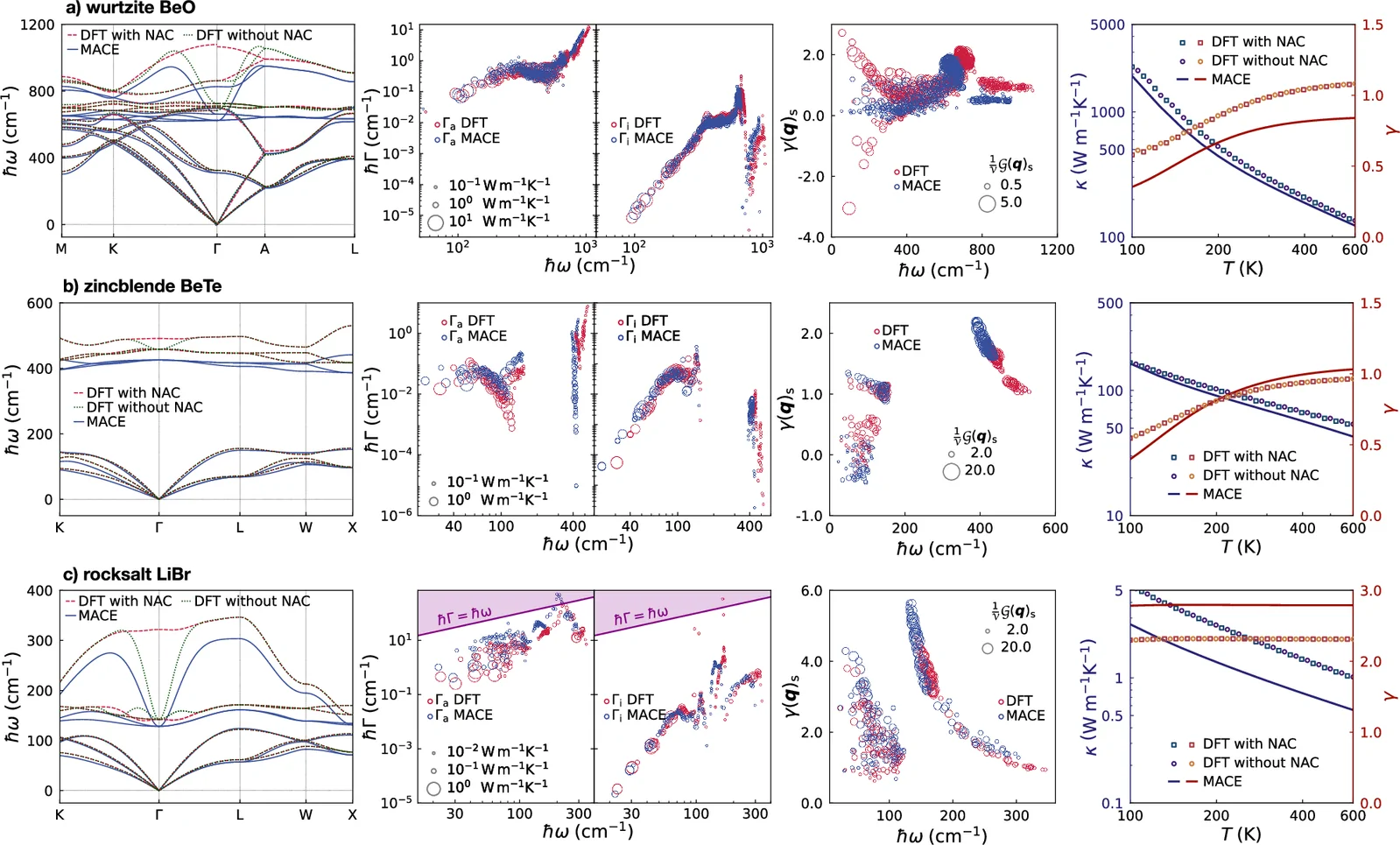
Phonon dispersion, linewidth & mode-Grüneisen distributions, along with macroscopic conductivity & Grüneisen parameter. **a** wurtzite BeO, **b** zincblende BeTe and **c** rocksalt LiBr. DFT-PBE values are computed using the data from refs. ^48,49^, while MACE values are computed using the MACE-MP-0 fMLP^23^. For the DFT-PBE phonons, we show the effect of including (red) or omitting (green) the long-range non-analytical contribution (NAC); MACE-MP-0 does not fully account for NAC (see text). In the frequency-linewidth distributions at 300 K, we resolve the anharmonic (Γ_*a*_) and isotopic (Γ_*i*_) parts of the linewidths; the markers' areas are proportional to the contribution of the given phonon mode to the conductivity. Similarly, the mode Grüneisen parameter distribution against phonon energy is evaluated at 300 K with marker size describing the contribution of the modes to the macroscopic Grüneisen parameter.

To understand the microscopic origin of the discrepancies between the DFT-PBE and MACE-MP-0 conductivities and Grüneisen parameters, we select three representative materials—wurtzite BeO, zincblende BeTe, and rocksalt LiBr—and show in Fig. 2 their phonon band structures, the macroscopic conductivity and Grüneisen resolved in terms of contributions of microscopic phonon modes. Examining the phonon band structures in the first column, we observe that MACE-MP-0 tends to underestimate the high-frequency optical modes compared to DFT-PBE. In particular, we plot the DFT-PBE phonons with and without the long-range non-analytical correction (NAC) term^60^. The NAC term is responsible for the energy splitting between the longitudinal-optical and transverse-optical modes in polar dielectrics^60^, and is not fully considered in fMLPs trained using a radial cutoff (e.g., for MACE-MP-0^23^ such cutoff is 6 Å, while it is 5 Å for SevenNet^24^, CHGNet^22^, and M3GNet^21^). This explains why the DFT-PBE phonons without NAC are in closer agreement with the MACE-MP-0 phonons. Importantly, even without NAC, DFT-PBE energies for the optical phonon modes tend to be higher than MACE-MP-0 energies, confirming the general tendency of MACE-MP-0 to underestimate phonon energies^36^. We show in Supplementary Fig. 3 that both the underestimation of the optical phonon energies and the inclusion or omission of NAC have negligible effect on the specific heat at constant volume—because in these materials the specific heat is dominated by acoustic phonons. Figure 2 also shows that including or omitting the NAC term has a negligible impact on *κ*(*T*) in BeO, BeTe, and LiBr. This can be understood from the third column of Fig. 2, where we show that in these materials a significant amount of heat is carried by low-energy acoustic phonons that are negligibly affected by NAC, as well as by the systematic phonon softening. Specifically, we report the energy-linewidth distributions, resolving the anharmonic and isotopic parts of the linewidths (Γ_a_(**q**)_*s*_ and Γ_i_(**q**)_*s*_, respectively), and quantifying how much each phonon contributes to the conductivity with the single-mode conductivity contribution ${{{\mathcal{K}}}}{({{{\bf{q}}}})}_{s}$ defined in Eq. (1). We note, in passing, that while for the 103 materials considered here the NAC term has negligible influence on thermal transport, it could be important in materials where significant heat is carried by optical modes. SOTA fMLPs do not account for NAC; its inclusion will be the subject of future work. In finite-displacement DFT calculations, real-space interatomic force constants partially account for long-range interactions (up to the range set by the supercell), while the NAC term describes the remaining dipole–dipole contribution^61^. Similarly, in fMLPs, this long-range contribution is partially accounted for up to the model cutoff. Since the fMLP cutoffs used here are larger than the real-space range sampled by the finite-displacement supercells used to compute phonons, the finite-supercell part of the long-range interaction is contained within the fMLP receptive field. Therefore, it is natural to use the DFT reference without the NAC contribution to calculate thermal conductivity and the Grüneisen parameter; the inclusion of the remaining long-range dipole–dipole contribution will be the subject of future work.

The most accurate zero-shot predictions for the energy-linewidth distributions (for BeO in Fig. 2a) correspond to a discrepancy of  ~ 7% between *κ*(*T*) predicted from fMLP (MACE-MP-0) and DFT. We stress that fMLPs make it possible to obtain this accuracy at a computational cost that is more than 3 orders of magnitude lower than DFT (see Supplementary Fig. 6). Figure 2b shows that compatibility between *κ*(*T*) and γ(*T*) predicted from DFT or fMLP can also result from cancellation of microscopic errors. E.g., in BeTe there are visible differences in the energy-linewidth distributions, and these largely cancel out when integrated to determine the conductivity. Finally, Fig. 2c illustrates that discrepancies in the energy-linewidth distributions do not always compensate and can translate into significant differences (a factor of 2) in *κ*(*T*). We also note that, depending on the chemical composition, the anharmonic linewidths at room temperature can dominate over the isotopic linewidth (e.g., in BeO) or not (e.g., in BeTe). Similar cancellation of microscopic errors can also emerge for the Grüneisen parameter, as shown in Fig. 2b.

The results above motivate us to quantitatively investigate when agreement between DFT and fMLP conductivities and Grüneisen parameters is obtained as a result of accurately described microscopic harmonic and anharmonic vibrational properties, or because of compensating errors. Starting from conductivity, we quantify the macroscopic discrepancies using the Symmetric Relative Error (SRE),

3 $$\,{{\rm{SRE}}}\,\left[\kappa \right]=2\frac{\left\vert {\kappa }_{{{{\rm{fMLP}}}}}-{\kappa }_{{{{\rm{DFT}}}}}\right\vert }{{\kappa }_{{{{\rm{fMLP}}}}}+{\kappa }_{{{{\rm{DFT}}}}}}\,.$$

**Fig. 3:**
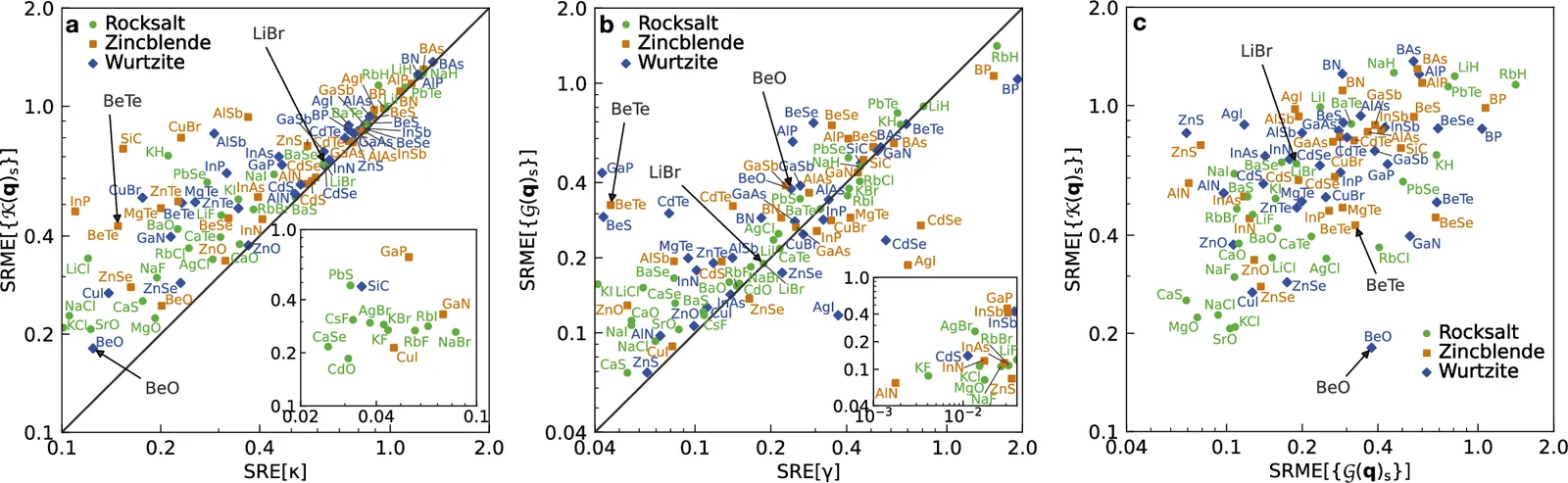
Symmetric relative (mean) errors, SRE (SRME). Panels show errors in conductivity (**a**), Grüneisen parameter (**b**), and their correlation (**c**) in 103 chemically and structurally diverse compounds. For conductivity (panel a), large discrepancies between DFT-PBE and mp-fMLP (MACE-MP-0) on microscopic, single-phonon conductivity contributions are described by large conductivity SRME (4). The microscopic SRME metric is more informative than the macroscopic SRE metric, since it captures the cases where microscopic (in)accuracy translates into macroscopic (in)accuracy—e.g., in BeO (LiBr) a low (high) SRME implies a low (high) SRE—and, because SRME is a sum of non-negative contributions, it detects also cases in which compensation of microscopic errors occurs (e.g., BeTe exhibits high SRME but low SRE). For Grüneisen parameter (panel b), the behavior of macroscopic (SRE, Eq. (5)) and microscopic (SRME, Eq. (6)) errors is more complex due to the possibility of having both positive and negative microscopic contributions, which can lead to both cancellation of single-phonon errors and amplification of macroscopic relative errors when large negative and positive contributions coexist, as discussed in the text. However, most of the materials studied here (**b**) display large SRME and positive microscopic contributions to the Grüneisen parameter, implying that cancellation of errors can be detected, as for the conductivity, by large SRME and low SRE. The insets in panels a and b show materials with very low SRE, while panel c displays the comparison of SRME in thermal conductivity and Grüneisen parameter, demonstrating no clear correlation.

Next, to quantify the error on the microscopic phonon properties, we introduce the Symmetric Relative Mean Error (SRME) on the single-phonon contribution to *κ*:

4 $$\begin{array}{l}\,{{\rm{SRME}}}\,\left[\{{{{\mathcal{K}}}}{({{{\bf{q}}}})}_{s}\}\right]=\frac{2}{{N}_{c}{{{\mathcal{V}}}}}\frac{{\sum }_{{{{\bf{q}}}}s}\left\vert {{{{\mathcal{K}}}}}_{{{{\rm{fMLP}}}}}{({{{\bf{q}}}})}_{s}-{{{{\mathcal{K}}}}}_{{{{\rm{DFT}}}}}{({{{\bf{q}}}})}_{s}\right\vert }{{\kappa }_{{{{\rm{fMLP}}}}}+{\kappa }_{{{{\rm{DFT}}}}}}, \end{array}$$

where ${{{{\mathcal{K}}}}}_{{{{\rm{DFT}}}}}{({{{\bf{q}}}})}_{s}$ refers to the single-phonon conductivity (term inside square brackets in Eq. (1)) evaluated using DFT, and ${{{{\mathcal{K}}}}}_{{{{\rm{fMLP}}}}}{({{{\bf{q}}}})}_{s}$ refers to the same expression evaluated using fMLP. Fig. 3a illustrates that a large SRME generally implies large SRE, as SRE gives a lower bound on the SRME. Importantly, knowing both SRE and SRME enables us to identify when microscopic error compensation occurs—this is indicated by a large SRME but small SRE. We note that the SRME[${{{\mathcal{K}}}}{({{{\bf{q}}}})}_{s}$] error can stem from discrepancies in the harmonic (second-order) or anharmonic (third-order) force constants. In Supplementary Fig. 2, we resolve how errors on harmonic and anharmonic vibrational properties contribute to SRME.

Analyzing macroscopic and microscopic errors on the Grüneisen parameter requires additional considerations. In fact, while the positive-definite *κ* can be resolved in terms of microscopic non-negative single-phonon contributions, the single-phonon contributions to the Grüneisen parameter can assume positive or negative sign, and yield a macroscopic Grüneisen parameter with positive or negative sign depending on their relative strength. To take this into account, we define the SRE for the Grüneisen parameter as

5 $$\,{{\rm{SRE}}}\,\left[\upgamma \right]=2\frac{\left\vert {\upgamma }_{{{{\rm{fMLP}}}}}-{\upgamma }_{{{{\rm{DFT}}}}}\right\vert }{\left\vert {\upgamma }_{{{{\rm{fMLP}}}}}\right\vert+\left\vert {\upgamma }_{{{{\rm{DFT}}}}}\right\vert }\,,$$

and the corresponding SRME as

6 $$\begin{array}{l}\,{{\rm{SRME}}}\,\left[\{{{{\mathcal{G}}}}{({{{\bf{q}}}})}_{s}\}\right]=2\frac{{\sum }_{{{{\bf{q}}}}s}\left\vert {{{{\mathcal{G}}}}}_{{{{\rm{fMLP}}}}}{({{{\bf{q}}}})}_{s}-{{{{\mathcal{G}}}}}_{{{{\rm{DFT}}}}}{({{{\bf{q}}}})}_{s}\right\vert }{{\sum }_{{{{\bf{q}}}}s}\left\vert {{{{\mathcal{G}}}}}_{{{{\rm{fMLP}}}}}{({{{\bf{q}}}})}_{s}\right\vert+\left\vert {{{{\mathcal{G}}}}}_{{{{\rm{DFT}}}}}{({{{\bf{q}}}})}_{s}\right\vert }\,,\ \ \end{array}$$

where ${{{{\mathcal{G}}}}}_{{{{\rm{DFT}}}}}{({{{\bf{q}}}})}_{s}$ refers to the single-phonon contribution to the Grüneisen parameter (term inside square brackets in Eq. (2)) evaluated using DFT, and ${{{{\mathcal{G}}}}}_{{{{\rm{fMLP}}}}}{({{{\bf{q}}}})}_{s}$ refers to the same expression evaluated using fMLP. These definitions yield a non-negative Grüneisen SRME (6), with maximal value 2 and minimal value 0, in analogy to the conductivity SRME (4).

We have seen that because the microscopic conductivity contributions are non-negative, the SRE provides a lower bound on the SRME. In contrast, microscopic Grüneisen parameter contributions can range from positive to negative, implying four distinct possible scenarios for SRME and SRE: (i) when both SRE and SRME are low, the fMLP’s prediction is accurate (e.g., zincblende AlN in Fig. 3b); (ii) when both are high, large microscopic errors add into a large error for the macroscopic Grüneisen parameter, and the prediction is clearly inaccurate (e.g., wurtzite BP in Fig. 3b); (iii) when SRME is high but SRE is low, predictions for the macroscopic Grüneisen parameter appear accurate, but are not reliable because they originate from a fortuitous cancellation of microscopic errors in the underlying phonon physics (e.g., wurtzite GaP in Fig. 3b); and (iv) when SRE is high and SRME is low, small deviations can induce significant relative errors in cases where the macroscopic Grüneisen parameter vanishes due to large-magnitude and compensating positive and negative microscopic single-phonon contributions (e.g., zincblende AgI in Fig. 3b). While cases (i), (ii), and (iii) are totally analogous to those discussed for the positive-definite conductivity, case (iv) emerges only for signed thermomechanical quantities such as the Grüneisen parameter. For most of the 103 materials in the phononDB-PBE database, the macroscopic Grüneisen parameter at 300 K is positive, and SRME exceeds SRE, indicating that case (iv) occurs rarely and that for these materials, SRME is overall more informative than SRE in assessing the overall accuracy with which the Grüneisen parameter is described.

Importantly, we note that the SRME in the Grüneisen parameter is not clearly correlated with the SRME in the conductivity, see Fig. 3c. This demonstrates the importance of analyzing both conductivity and Grüneisen to assess the accuracy and physics-awareness of the fMLPs.

### Accuracy of various SOTA foundation models

**Fig. 4:**
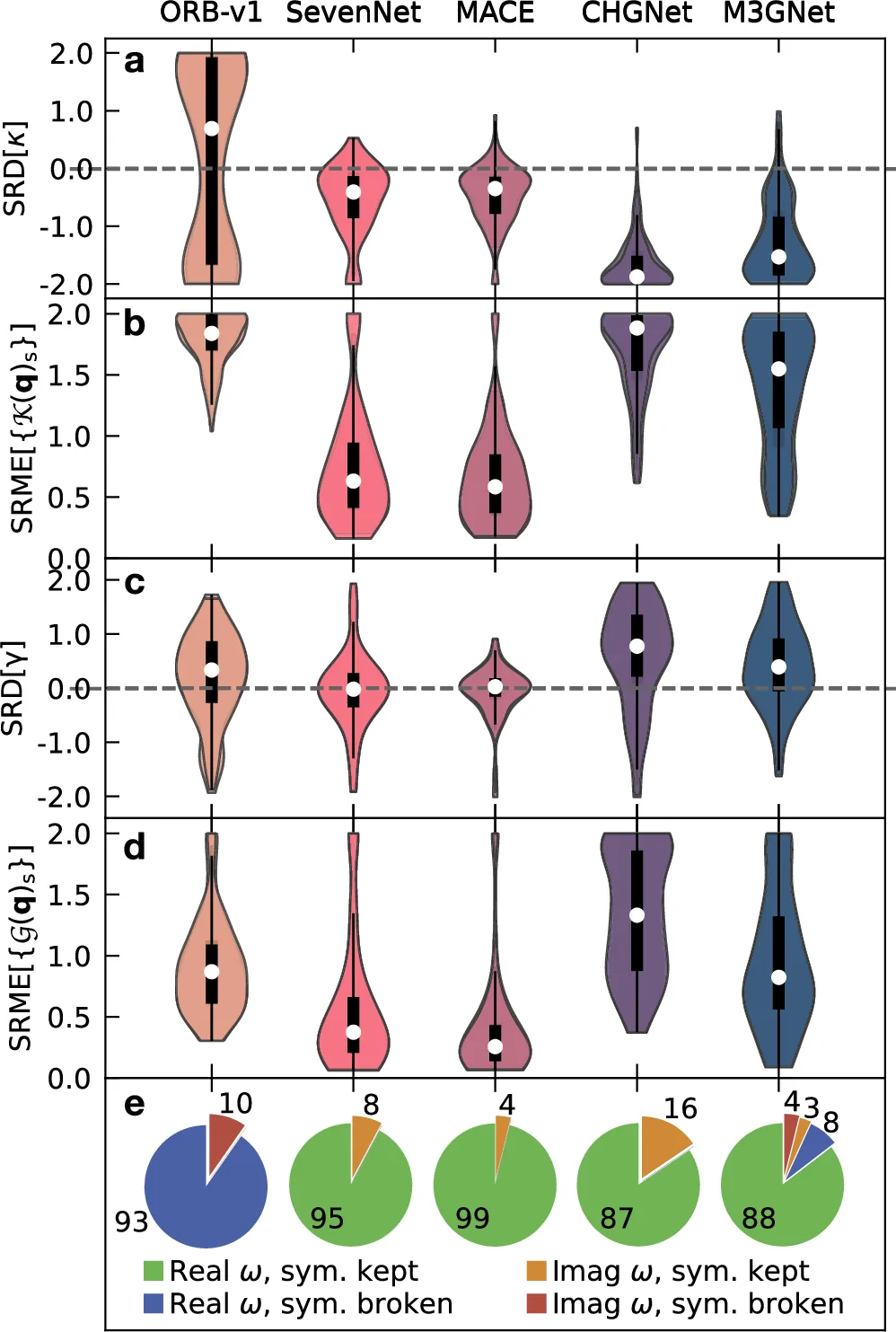
Performance of foundation machine-learning potentials in predicting thermal conductivity and Grüneisen parameter. **a**, **c** violin plots for the symmetric relative difference (SRD) in the total Wigner conductivity (7) and in the total Grüneisen parameter, respectively, for the materials in phononDB-PBE simulated with ORB-v1-MPtraj^47^, SevenNet^24^, MACE-MP-0^23^, CHGNet^22^, M3GNet^21^. **b**, **d** are symmetric relative mean errors, $\,{\mbox{SRME}}\,\left[\{{{{\mathcal{K}}}}{({{{\bf{q}}}})}_{s}\}\right]$ and $\,{\mbox{SRME}}\,\left[\{{{{\mathcal{G}}}}{({{{\bf{q}}}})}_{s}\}\right]$, respectively, in the same materials database. In all panels, the medians are marked with white scatter points, the widths of the boxes represent the interquartile range, and the whiskers show the range of data points without outliers. The pie charts in (**e**) display the number of compounds with non-negative frequencies that either retained the correct crystal symmetry after unconstrained relaxation or did not (green and blue, respectively); we also show whether structural instabilities (imaginary phonon frequencies) were observed in relaxations performed with (orange) or without (red) enforcing symmetries. Unstable structures with imaginary phonon frequencies were included considering *κ*_fMLP_ = 0 and γ_fMLP_ = 0, i.e., SRD = − 2, SRME = 2.

Here, we utilize SRE and SRME for *κ* and γ to study the accuracy of non-proprietary mp-fMLPs on the structures contained in the phononDB-PBE database^48,49^, comparing DFT predictions against M3GNet^21^, CHGNet^22^, MACE-MP-0^23^, SevenNet^24^, and ORB-v1-MPtraj^47^ in Fig. 4. To determine if a particular mp-fMLP tends to systematically overestimate or underestimate the conductivity and Grüneisen compared to DFT-PBE, we rely on the Symmetric Relative Difference (SRD). For the conductivity this is defined as:

7 $${{{\rm{SRD}}}}\left[\kappa \right]=2\frac{{\kappa }_{{{{\rm{fMLP}}}}}-{\kappa }_{{{{\rm{DFT}}}}}}{{\kappa }_{{{{\rm{fMLP}}}}}+{\kappa }_{{{{\rm{DFT}}}}}}\,,$$

and similarly for the Grüneisen parameter,

8 $${{{\rm{SRD}}}}\left[\upgamma \right]=2\frac{{\upgamma }_{{{{\rm{fMLP}}}}}-{\upgamma }_{{{{\rm{DFT}}}}}}{| {\upgamma }_{{{{\rm{fMLP}}}}}|+| {\upgamma }_{{{{\rm{DFT}}}}}| }\,.$$

In both cases the SRD ranges from −2 to +2, corresponding to fMLP underestimating and overestimating the reference DFT values, respectively.

Figures 4a, c summarize in violin plots the SRD for the compounds in the phononDB-PBE database. Unstable structures with imaginary phonon frequencies were included considering *κ*_fMLP_ = 0 and γ_fMLP_ = 0, i.e., SRD = − 2, SRME = 2. This analysis reveals that even the most accurate mp-fMLP tends to underestimate the thermal conductivity, as a consequence of underestimating vibrational frequencies^36^, and in some cases overestimating anharmonic linewidths. In contrast, such a systematic error is not observed for the Grüneisen parameter, confirming that the analyses of *κ* and γ provide complementary information on the accuracy of fMLPs.

To examine how the macroscopic SRD in Fig. 4a, c are influenced by microscopic errors, we show in Fig. 4b, d violin plots for SRME in the mode contribution to conductivity and Grüneisen parameter. For the conductivity, this directly relates to microscopic error compensation, whereas the relation between SRD (or SRE) and SRME in the Grüneisen parameter is more complex due to the possibility of negative values and amplification of the relative error.

Among the mp-fMLPs tested, the one that shows the highest accuracy is MACE-MP-0^23^, which produces zero-shot conductivities compatible within a factor of two of the DFT-PBE values for 69% of the materials in the phononDB-PBE database^48,49^. The zero-shot Grüneisen parameter is compatible within a factor of two for 67% of the materials.

To quantify the overall accuracy of fMLPs in predicting the macroscopic conductivity and Grüneisen parameter over a materials database, without resolving the possible compensation of microscopic errors, it is informative to consider the mean of the modulus of the deviations, i.e., the mean of the distribution of SRE (3)—we prefer this over the mean of the SRD distribution, as the latter can be close to zero in the presence of a very broad but sign-symmetric distribution.

Starting from the conductivity, the mean for SRE[*κ*] and mean for SRME[$\{{{{\mathcal{K}}}}{({{{\bf{q}}}})}_{s}\}$] (Table 1) are comparable in the absence of cancellation of microscopic errors, while the mean SRE[*κ*] is significantly lower than mean SRME[$\{{{{\mathcal{K}}}}{({{{\bf{q}}}})}_{s}\}$] when cancellation of microscopic errors occurs. As anticipated, this relation is not necessarily respected for SRE[γ] and SRME[$\{{{{\mathcal{G}}}}{({{{\bf{q}}}})}_{s}\}$], however the materials in focus here have non-negligible Grüneisen parameter with dominating positive single-phonon contributions, and therefore mostly display a relation between SRE[γ] and SRME[$\{{{{\mathcal{G}}}}{({{{\bf{q}}}})}_{s}\}$] analogous to the one for the conductivity.

**Table 1:** Benchmark metrics for foundation machine-learning potentials

	ORB-v1	7Net	MACE	CHGNet	M3GNet
mean ${{{\rm{SRE}}}}[\kappa ]$	1.555	0.605	0.528	1.695	1.340
mean ${{{\rm{SRME}}}}[\{{{{\mathcal{K}}}}{({{{\bf{q}}}})}_{s}\}]$	1.786	0.761	0.669	1.717	1.409
mean ${{{\rm{SRE}}}}[\upgamma ]$	0.667	0.499	0.282	0.976	0.694
mean ${{{\rm{SRME}}}}[\{{{{\mathcal{G}}}}{({{{\bf{q}}}})}_{s}\}]$	0.899	0.557	0.374	1.345	0.941
F1 (11/9/2024)^34^	0.763	0.724	0.669	0.613	0.569

The mean of the symmetric relative (mean) errors, SRE[*κ*], $\,{\mbox{SRME}}\,[\{{{{\mathcal{K}}}}{({{{\bf{q}}}})}_{s}\}]$, SRE[γ] and $\,{\mbox{SRME}}\,[\{{{{\mathcal{G}}}}{({{{\bf{q}}}})}_{s}\}]$ introduced here resolve the accuracy of fMLPs in describing interatomic forces and derived structural and thermal properties. The Matbench F1^34^ descriptor is based on atomic energies and resolves thermodynamic stability. Different (similar) values for SRE and SRME indicate the presence (absence) of cancellation of errors on microscopic vibrational properties. The lower the SRE is, the higher the accuracy is in predicting macroscopic *κ*(300K) and γ(300K). The lower the SRME is, the higher the accuracy is in predicting both microscopic vibrational properties and macroscopic *κ*(300K) and γ(300K). The higher the F1 score is, the higher the accuracy is in describing thermodynamic stability.

Importantly, we note that our benchmarks on SRME[$\{{{{\mathcal{K}}}}{({{{\bf{q}}}})}_{s}\}$], SRME[$\{{{{\mathcal{G}}}}{({{{\bf{q}}}})}_{s}\}$] and SRE[*κ*], SRE[γ] evaluate the accuracy of fMLPs in predicting experimentally observable conductivity and Grüneisen parameter, which are macroscopic properties determined not only by a single (local-energy-minima) point of the PES, but also by its second- and third-order derivatives (i.e., harmonic and anharmonic interatomic forces^62^). Because these derivatives expose even subtle discontinuities in the PES, our metrics provide information that is physically richer and more robust compared to established energy-based metrics, and overall stricter in measuring both the utility of fMLPs and the physics encoded in the smoothness of the PES^45^. Table 1 highlights how fMLPs that are highly accurate according to tests based on interaction energies can be inaccurate in predicting crystal structures, interatomic forces, and thermomechanical properties. Conversely, for the materials studied so far, fMLPs that are highly accurate according to the SRME[$\{{{{\mathcal{K}}}}{({{{\bf{q}}}})}_{s}\}$] and SRME[$\{{{{\mathcal{G}}}}{({{{\bf{q}}}})}_{s}\}$] metrics are also highly ranked by energy-based tests, and Fig. 4e also shows that low SRME[$\{{{{\mathcal{K}}}}{({{{\bf{q}}}})}_{s}\}$] is correlated with correctly describing crystal symmetries.

Importantly, the MPtrj dataset, used to train the fMLPs discussed in this work, includes multiple DFT simulations for the same structure computed with DFT settings of different accuracy (see, e.g., BeTe mp-252, which contains tasks mp-657299 and mp-1057046 that were computed with different k-point grids); this may introduce noise into the training data. In this study, to ensure consistency, we adopted the same DFT parameters as those used for the phononDB-PBE database^48,49^ in both conductivity and Grüneisen calculations. Therefore, even if the MPtrj and phononDB-PBE datasets were computed with the same DFT functional, the DFT thresholds in these two datasets are not always exactly the same, and the MPtrj dataset may contain noise related to variations in the stringency of the DFT thresholds. The strong improvement in the accuracy of fMLPs upon fine-tuning shows that these discrepancies are unimportant for assessing the performance of the fMLPs discussed in this work. However, as fMLPs continue to improve in accuracy, discrepancies between DFT and fMLP predictions—especially the residual few-percent differences after fine-tuning—may become comparable to the noise introduced by variations in the stringency of the DFT thresholds in the different datasets. Future work should therefore focus on generating more accurate training and reference DFT data for fMLPs, with higher accuracy, comprehensiveness, and quality^63^.

### Achieving first-principles accuracy via fine-tuning

Earlier, we discussed LiBr as a paradigmatic example where the zero-shot conductivity predictions from MACE-MP-0 are in strong disagreement—about a factor of two—with DFT-PBE reference data. Predictions for the Grüneisen parameter also show a 20% discrepancy. These discrepancies might be due to lack of relevant reference data in the MP dataset^46^ used to train MACE-MP-0, and in this section we introduce a fine-tuning protocol to correct them and achieve first-principles accuracy.

Employing the same DFT settings used to generate reference DFT data (see Methods for details), we prepared three distinct datasets for fine-tuning. Each dataset contains a set of different DFT-PBE frames—by frame we mean a set comprising forces, total energy, and stress computed from DFT for an atomic configuration perturbed from equilibrium. Perturbations consist of rattling (random atomic displacements drawn from a Gaussian distribution with standard deviation of 0.03 Å)^64^, and in some cases also a few-percent isotropic rescaling of volume. DFT frames have the same size as those used to compute the reference phononDB-PBE phonons, a 4 × 4 × 4 supercell of LiBr (512 atoms).

**Fig. 5:**
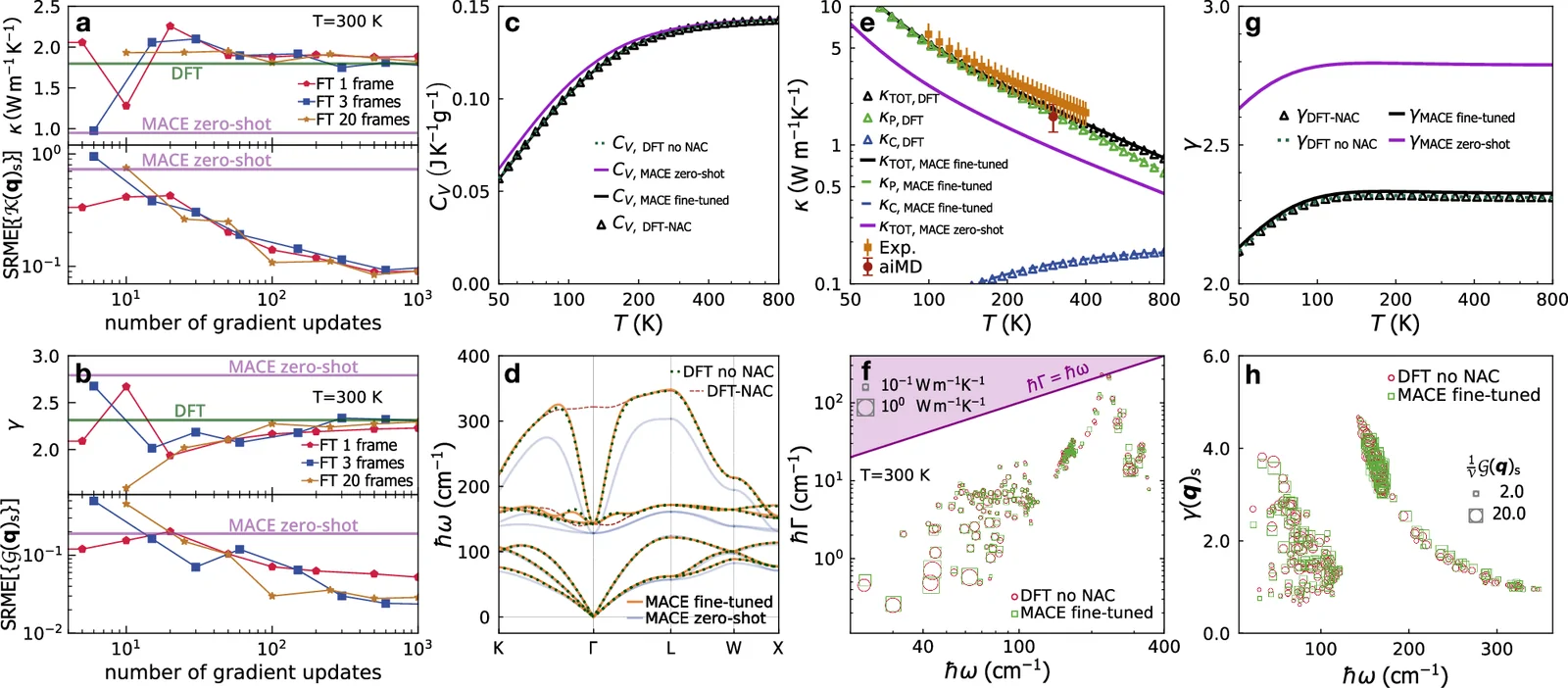
Achieving first-principles accuracy on LiBr’s vibrational & thermal properties through fine-tuning. The top of panel **a** (**b**) shows conductivity (Grüneisen parameter) as a function of the number of DFT-PBE supercell frames (see text) in the fine-tuning dataset, and the number of fine-tuning gradient updates. The MACE zero-shot prediction for conductivity (Grüneisen parameter) is purple; performing fine-tuning on one single DFT frame and 100 fine-tuning gradient updates yields compatibility within 7% for conductivity (5.5% for Grüneisen) with the reference DFT-PBE value (green), while datasets containing 3 frames with rattling and volume variations yield compatibility within 2% for conductivity (0.5% for Grüneisen) at 600 gradient updates. The bottom of panel **a** (**b**) shows that the symmetric relative mean errors, SRME$\left[\{{{{\mathcal{K}}}}{({{{\bf{q}}}})}_{s}\}\right]$ and SRME$\left[\{{{{\mathcal{G}}}}{({{{\bf{q}}}})}_{s}\}\right]$, converge to a minimum upon increasing the number of gradient updates, and larger datasets yield faster convergence. After fine-tuning on 3 frames for 600 gradient updates, good agreement between fine-tuned MACE and DFT-PBE is observed for: specific heat (**c**), phonons with and without the non-analytical correction (NAC) term (**d**), microscopic phonon energy-linewidth (**f**) and mode Grüneisen parameter distributions (**h**), macroscopic *κ*(*T*) (**e**) and γ(*T*) (**g**). We note that after fine-tuning, our Wigner transport equation (WTE) *κ*(*T*) predictions agree with experiments^65^, as well as with predictions from ab-initio (DFT-PBE) molecular-dynamics (aiMD) at 300 K^57^.

We generated three training datasets for fine-tuning, which contain: (i) one single frame at equilibrium volume and with atomic rattling; (ii) 3 frames, out of which 2 feature  ± 1% isotropic volume rescaling and rattling, plus the frame in (i) at equilibrium volume and with rattling; (iii) 20 frames, out of which 10 are at equilibrium volume with different rattling, and 10 frames with rattling and volume variations of  ± 0.25 %,  ± 0.50%,  ± 1%,  ± 2%, and  ± 5%. Using these datasets, we performed fine-tuning training with number of gradient updates ranging from 5 to 1000. Training root-mean-square errors (RMSE) on energy, forces, and stresses are discussed in Supplementary Fig. 7. Validation was performed relying on reference data for LiBr in the phononDB-PBE database, in particular Fig. 5a, b show the validation error on SRME[$\{{{{\mathcal{K}}}}{({{{\bf{q}}}})}_{s}\}$] and SRME[$\{{{{\mathcal{G}}}}{({{{\bf{q}}}})}_{s}\}$] respectively. Fig. 5a highlights how fine-tuning relying on the 3-frame dataset (ii) and for 600 gradient updates yields a validation error on SRME[$\{{{{\mathcal{K}}}}{({{{\bf{q}}}})}_{s}\}$]<0.1, which corresponds to compatibility within 2% with the reference DFT-PBE conductivity *κ*(300 K). In the case of the Grüneisen parameter, in Fig. 5b, the SRME[$\{{{{\mathcal{G}}}}{({{{\bf{q}}}})}_{s}\}$] is <0.03, which corresponds to compatibility within 0.5% with the reference DFT-PBE Grüneisen parameter γ(300 K). We note that low ${{{\rm{SRME}}}}[\{{{{\mathcal{K}}}}{({{{\bf{q}}}})}_{s}\}]$ and ${{{\rm{SRME}}}}[\{{{{\mathcal{G}}}}{({{{\bf{q}}}})}_{s}\}]$ imply also strong compatibility with reference phononDB-PBE data for specific heat (Fig. 5c), phonons (Fig. 5d), microscopic energy-linewidth distribution (Fig. 5f) and mode Grüneisen parameter (Fig. 5h). The accuracy of the fine-tuned fMLP in predicting the macroscopic, experimentally measurable conductivity was evaluated over a test set including: ab-initio molecular dynamics (aiMD)^57^ performed at 300 K using the same PBE functional used in our work; experimental data^65^ in the temperature range 100–800 K. Fig. 5e highlights how our predictions agree, within error bars, both in trend and magnitude with experiments (error bars show the compatibility threshold of 20% discussed in ref. ^65^), as well as with independent aiMD predictions^57^. We also note that using the larger fine-tuning dataset (iii) does not show significant improvements in accuracy, and the single-frame dataset (i) allows us to reduce conductivity discrepancies from 47% to 7% in 100 fine-tuning gradient updates. Fig. 5g shows that the obtained Grüneisen parameter is compatible with the reference DFT-PBE data (the maximum deviation in the temperature range 50–800 K is smaller than 0.7%); comparisons against experiments on thermal expansion or Grüneisen parameter are not reported because, to the best of our knowledge, these are not available in the literature.

Finally, we emphasize that employing the WTE is essential for accurately describing heat transport in LiBr over a broad temperature range. In fact, with increasing temperature the tunneling conductivity *κ*_*C*_ becomes more important and non-negligible compared to the propagation conductivity *κ*_*P*_, and the total WTE conductivity *κ*_*T*_(*T*) = *κ*_*P*_(*T*) + *κ*_*C*_(*T*) is in better agreement with the experimental trend compared to the BTE (propagation-only) conductivity *κ*_*P*_(*T*).

We highlight how with one single fine-tuning procedure we obtained quantitatively accurate predictions for both thermal conductivity and Grüneisen parameter (evaluated with the volume-derivative method, see Supplementary Information for workflow). More generally, additional properties like temperature-dependent bulk modulus and thermal-expansion coefficient can be directly obtained from fine-tuned fMLPs by direct free energy minimization within QHA^41,66^ (see Methods). This represents an advantage compared to established methods such as: (i) compressive sensing, which requires different DFT datasets at different volumes to compute these properties (see, e.g., ref. ^64^ for a thorough discussion of compressive sensing methods applied to simulations of vibrational properties); (ii) training a conventional (non-foundation) machine-learning potential from scratch, which requires a large training dataset to obtain accurate results. Importantly, fMLPs enable zero-shot predictions that are rapidly becoming increasingly more accurate^45^; this is an advantage compared to established compressive-sensing or non-foundation methods, which require supercell DFT calculations for direct predictions or training.

Another important application of fMLPs is that of high-throughput materials screening, which aims at identifying optimal materials for target applications by systematically calculating and comparing the properties of a large number of materials with different compositions and structures^15,37,46,67–71^. In the Supplementary Information, we demonstrate that SOTA fMLPs can be simultaneously fine-tuned for multiple compositions (rather than independently fine-tuning different compositions), a property that may be highly beneficial to minimize the computational cost of high-throughput screening applications. In particular, we show that simultaneous fine-tuning in three compositions yields conductivity and Grüneisen parameter compatible within 5% with DFT reference. This fine-tuning was particularly data efficient, as it was performed using only three atomic configurations: a single rattled supercell at equilibrium volume per material; two unit-cell-sized configurations at expanded and contracted volume, respectively, to describe stresses.

## Discussion

We introduced a framework that leverages foundation models for atomistic materials chemistry^21–33^, the Wigner formulation of heat transport^38,39^, and the quasi-harmonic approach for thermal expansion^41,44^ to address the major challenges of current techniques for designing heat-management materials: high computational cost, limited transferability, or lack of physics awareness. We employ this framework to quantify how the accuracy of fMLPs in describing many-body interatomic forces connects to their accuracy in predicting experimentally observable thermomechanical properties. We rely on these insights to define physically interpretable, robust, and stringent benchmark metrics that expose how subtle artifacts or inaccuracies in the smoothness of the Born-Oppenheimer PES encoded by fMLPs affect the prediction of constant-volume observables such as the thermal conductivity, and volume-deforming properties such as the Grüneisen parameter. Because our metrics test not only single-energy values of the PES but also its derivatives (smoothness), they are physically richer, more robust, and stricter compared to established single-energy metrics^45^, as recognized by several works^45,72–74^ released after the first preprint version of this manuscript. For these reasons, shortly after the preprint version of this work, our *κ*_SRME_ conductivity metric (Eq. (4)) has been included on the Matbench Discovery platform^45^ and recognized as a key metric to assess both the utility and physical accuracy of the potential energy surface described by fMLPs^45,72–74^. These statements are supported by very recent work from Orbital Materials^73^: they employed the metrics discussed here (in the first preprint version of this manuscript) to develop the Orb-v3 model, which displays significantly improved zero-shot accuracy compared to the model Orb-v1 discussed in this manuscript.

We also demonstrated that zero-shot fMLP predictions for conductivity and Grüneisen parameter can be within a factor of 2 accuracy from DFT reference data, and near DFT accuracy (within 2%) can be obtained through fine-tuning fMLPs. Most importantly, we showed how fine-tuned fMLPs also enable the prediction of several thermomechanical properties at significantly reduced computational cost compared to current DFT-based methods (e.g., based on systematic finite-differences^48^, compressive sensing^64^, or density-functional perturbation theory^75^), as well as compared to conventional (composition-specific, non-foundation) machine-learning potentials that require computationally expensive training from scratch. Importantly, our physics-aware framework also overcomes the reliability issues (unknown unknowns) of current end-to-end machine-learning methods for predicting vibrational or thermal properties of materials with arbitrary composition and structure.

Finally, we have shown that our framework enables the screening or prediction of materials that violate semiclassical Boltzmann transport, which are crucial for applications ranging from thermal insulation^11,39^ to neuromorphic computing^12^. It is worth mentioning that after posting the preprint of this manuscript on arXiv, the effectiveness of fMLP-based computational frameworks in predicting thermomechanical properties has also been discussed by Microsoft Research AI for Science, which employed the fMLP MatterSim^25^ to perform high-throughput screening of materials with extreme lattice thermal conductivities^76^. Ultimately, we have shown that combining the Wigner Transport Equation with fMLP enables the theory-driven optimization, design, and discovery of materials for energy and information-management technologies.

## Methods

### Vibrational properties relevant to compute *κ*(*T*) and γ(*T*)

In this section, we discuss the relation between interatomic vibrational energies and the quantities that appear in the WTE thermal conductivity expression (1) and in the formula for the Grüneisen parameter (2). The starting point is the Born-Oppenheimer Hamiltonian for atomic vibrations^39^ expanded up to anharmonic third-order^56,77–83^ in the atomic displacements from equilibrium, and accounting for energy perturbations due to the presence of isotopes ^84–88^.

9 $$\hat{H}=	 {\sum\limits_{{{{\bf{R}}}},b,\alpha }\frac{{\hat{p}}_{{{{\bf{R}}}}b\alpha }^{2}}{2{M}_{b}}+\frac{1}{2}\left.{\sum}_{{{{\bf{R}}}},b,\alpha \atop {{{{\bf{R}}}}}^{{\prime} },{b}^{{\prime} },{\alpha }^{{\prime} }}\frac{{\partial }^{2}V}{\partial {u}_{{{{\bf{R}}}}b\alpha }\partial {u}_{{{{{\bf{R}}}}}^{{\prime} }{b}^{{\prime} }{\alpha }^{{\prime} }}}\right| }_{{{{\rm{eq}}}}}{\hat{u}}_{{{{\bf{R}}}}b\alpha }{\hat{u}}_{{{{{\bf{R}}}}}^{{\prime} }{b}^{{\prime} }{\alpha }^{{\prime} }}\\ 	 {+\frac{1}{3!}\left.{\sum}_{{{{\bf{R}}}},b,\alpha \atop {{{{\bf{R}}}}}^{{\prime} },{b}^{{\prime} },{\alpha }^{{\prime} } {{{{\bf{R}}}}}^{{\prime\prime} },{b}^{{\prime\prime} },{\alpha }^{{\prime\prime} }}\frac{{\partial }^{3}V}{\partial {u}_{{{{\bf{R}}}}b\alpha }\partial {u}_{{{{{\bf{R}}}}}^{{\prime} }{b}^{{\prime} }{\alpha }^{{\prime} }}\partial {u}_{{{{{\bf{R}}}}}^{{\prime\prime} }{b}^{{\prime\prime} }{\alpha }^{{\prime\prime} }}}\right| }_{{{{\rm{eq}}}}}{\hat{u}}_{{{{\bf{R}}}}b\alpha }{\hat{u}}_{{{{{\bf{R}}}}}^{{\prime} }{b}^{{\prime} }{\alpha }^{{\prime} }}{\hat{u}}_{{{{{\bf{R}}}}}^{{\prime\prime} }{b}^{{\prime\prime} }{\alpha }^{{\prime\prime} }}\,\\ 	+\sum\limits_{{{{\bf{R}}}},b,\alpha }\left(\frac{{M}_{b}}{{m}_{b}}-1\right)\frac{{\hat{p}}_{{{{\bf{R}}}}b\alpha }^{2}}{2{M}_{b}}.$$

Here, ${\hat{p}}_{{{{\bf{R}}}}b\alpha }$ and ${\hat{u}}_{{{{\bf{R}}}}b\alpha }$ are the momentum and position-displacement operators for the atom *b* having isotope-averaged mass *M*_*b*_, position **R** + **τ**_*b*_ (**R** is the Bravais-lattice vector and **τ**_*b*_ the position in the crystal’s unit cell), and along the Cartesian direction *α*; the last term describes kinetic-energy perturbations induced by isotopes (*m*_*b*_ is the exact mass of the atom at **τ**_*b*_, we adopt the common approximation^81,84^ that the mass *m*_*b*_ depends only on the index *b* and not on the lattice vector **R**). The leading (harmonic) term in Eq. (9) determines the vibrational frequencies. In particular, the Fourier transform of the mass-rescaled Hessian of the interatomic potential yields the dynamical matrix at wavevector **q**,

10 $${\mathsf{D}}{({{{\bf{q}}}})}_{b\alpha,{b}^{{\prime} }{\alpha }^{{\prime} }}={\left.\sum \limits_{{{{\bf{R}}}}}\frac{{\partial }^{2}V}{\partial {u}_{{{{\bf{R}}}}b\alpha }\partial {u}_{{{{\bf{0}}}} {b}^{{\prime} }{\alpha }^{{\prime} }}}\right| }_{{{{\rm{eq}}}}}\frac{{e}^{-i{{{\bf{q}}}}\cdot ({{{\bf{R}}}}+{{{{\boldsymbol{\tau }}}}}_{b}-{{{{\boldsymbol{\tau }}}}}_{{b}^{{\prime} }})}}{\sqrt{{M}_{b}{M}_{{b}^{{\prime} }}}},$$

and by diagonalizing the dynamical matrix,

11 $${\sum}_{{b}^{{\prime} }{\alpha }^{{\prime} }}{\mathsf{D}}{({{{\bf{q}}}})}_{b\alpha,{b}^{{\prime} }{\alpha }^{{\prime} }}{{{\mathcal{E}}}}{({{{\bf{q}}}})}_{s,{b}^{{\prime} }{\alpha }^{{\prime} }}={\omega }^{2}{({{{\bf{q}}}})}_{s}{{{\mathcal{E}}}}{({{{\bf{q}}}})}_{s,b\alpha },$$

one obtains from the eigenvalues the phonon energies of the solid *ℏω*(**q**)_*s*_ (*s* is a band index ranging from 1 to 3*N*_at_, where *N*_at_ is the number of atoms in the unit cell), and from the eigenvectors ${{{\mathcal{E}}}}{({{{\bf{q}}}})}_{s,b\alpha }$ the displacement patterns of atom *b* in direction *α* for the phonon having wavevector **q** and mode *s*.

The vibrational energies *ℏω*(**q**)_*s*_ are sensitive to the thermally induced volume expansion, as quantified by the mode Grüneisen parameter, Eq. (2)^44^. In practice, the volume derivative is calculated using the Hellmann-Feynman theorem^66^,

12 $$\upgamma {({{{\bf{q}}}})}_{s}=-\frac{{{{\mathcal{V}}}}}{2\omega {({{{\bf{q}}}})}_{s}^{2}}{\sum}_{b\alpha,{b}^{{\prime} }{\alpha }^{{\prime} }}{{{{\mathcal{E}}}}}^{*}{({{{\bf{q}}}})}_{s,b\alpha }\frac{\partial {\mathsf{D}}{({{{\bf{q}}}})}_{b\alpha,{b}^{{\prime} }{\alpha }^{{\prime} }}}{\partial {{{\mathcal{V}}}}}{{{\mathcal{E}}}}{({{{\bf{q}}}})}_{s,{b}^{{\prime} }{\alpha }^{{\prime} }},$$

where the partial derivative of the dynamical matrix is obtained by computing the numerical differences at volumes expanded or contracted by 1%.

As discussed in detail in ref. ^39^, the velocity operator appearing in the WTE conductivity expression (1) is determined by the wavevector derivative of the square root of the dynamical matrix:

13 $${{\mathbf{\sf{v}}}}{({{{\bf{q}}}})}_{s,{s}^{{\prime} }}={\sum}_{b,\alpha,{b}^{{\prime} },{\alpha }^{{\prime} }}{{{{\mathcal{E}}}}}^{\star }{({{{\bf{q}}}})}_{s,b\alpha }\left[{{\nabla }_{{{{\bf{q}}}}}\sqrt{{\mathsf{D}}({{{\bf{q}}}})}}_{b\alpha,{b}^{{\prime} }{\alpha }^{{\prime} }}\right]{{{\mathcal{E}}}}{({{{\bf{q}}}})}_{{s}^{{\prime} },{b}^{{\prime} }{\alpha }^{{\prime} }}.$$

The anharmonic linewidths *ℏ*Γ_a_(**q**)_*s*_, which contribute to the total linewidths *ℏ*Γ(**q**)_*s*_ = *ℏ*Γ_a_(**q**)_*s*_ + *ℏ*Γ_i_(**q**)_*s*_ appearing in Eq. (1), are the energy broadenings due to anharmonic three-phonon interactions^77,78,81,89,90^. It can be shown^80^ that these are determined by the third derivative of the interatomic potential (see Eq. (9)),

14 $$\hslash {{\Gamma} }_{{{{\rm{a}}}}}{({{{\bf{q}}}})}_{s}=	 \frac{\pi }{{N}_{c}^{2}}{\sum}_{{{{{\bf{q}}}}}^{{\prime} }{{{{\bf{q}}}}}^{{\prime\prime} }\atop {s}^{{\prime} },{s}^{{\prime\prime} }}\left\{2\left[\bar{{\mathsf{N}}}{({{{{\bf{q}}}}}^{{\prime} })}_{{s}^{{\prime} }}-\bar{{\mathsf{N}}}{({{{{\bf{q}}}}}^{{\prime\prime} })}_{{s}^{{\prime\prime} }}\right]\delta \left(\omega {({{{\bf{q}}}})}_{s}+\omega {({{{{\bf{q}}}}}^{{\prime} })}_{{s}^{{\prime} }}-\omega {({{{{\bf{q}}}}}^{{\prime\prime} })}_{{s}^{{\prime\prime} }}\right)\right.\\ 	 \left.+\left[1+\bar{{\mathsf{N}}}{({{{{\bf{q}}}}}^{{\prime} })}_{{s}^{{\prime} }}+\bar{{\mathsf{N}}}{({{{{\bf{q}}}}}^{{\prime\prime} })}_{{s}^{{\prime\prime} }}\right]\delta \left[\omega {({{{\bf{q}}}})}_{s}-\omega {({{{{\bf{q}}}}}^{{\prime} })}_{{s}^{{\prime} }}-\omega {({{{{\bf{q}}}}}^{{\prime\prime} })}_{{s}^{{\prime\prime} }}\right]\right\}\times \\ 	 \left| {\sum}_{\alpha,{\alpha }^{{\prime} },{\alpha }^{{\prime\prime} }\atop b,{b}^{{\prime} },{b}^{{\prime\prime} },{{{{\bf{R}}}}}^{{\prime} }{{{{\bf{R}}}}}^{{\prime\prime} }}\frac{{\partial }^{3}V}{\partial {u}_{{{{\bf{0}}}}b\alpha }\partial {u}_{{{{{\bf{R}}}}}^{{\prime} }{b}^{{\prime} }{\alpha }^{{\prime} }}\partial {u}_{{{{{\bf{R}}}}}^{{\prime\prime} }{b}^{{\prime\prime} }{\alpha }^{{\prime\prime} }}}{{{\mathcal{E}}}}{({{{\bf{q}}}})}_{s,b\alpha }{{{\mathcal{E}}}}{({{{{\bf{q}}}}}^{{\prime} })}_{{s}^{{\prime} },{b}^{{\prime} }{\alpha }^{{\prime} }}{{{\mathcal{E}}}}{({{{{\bf{q}}}}}^{{\prime\prime} })}_{{s}^{{\prime\prime} },{b}^{{\prime\prime} }{\alpha }^{{\prime\prime} }}\right.\\ 	 {\left.\sqrt{\frac{{\hslash }^{3}}{8}}\frac{{{\Delta }}({{{\bf{q}}}}+{{{{\bf{q}}}}}^{{\prime} }+{{{{\bf{q}}}}}^{{\prime\prime} }){e}^{-i[{{{\bf{q}}}}\cdot {{{{\boldsymbol{\tau }}}}}_{b}+{{{{\bf{q}}}}}^{{\prime} }\cdot ({{{{\bf{R}}}}}^{{\prime} }+{{{{\boldsymbol{\tau }}}}}_{{b}^{{\prime} }})+{{{{\bf{q}}}}}^{{\prime\prime} }\cdot ({{{{\bf{R}}}}}^{{\prime\prime} }+{{{{\boldsymbol{\tau }}}}}_{{b}^{{\prime\prime} }})]}}{\sqrt{{M}_{b}{M}_{{b}^{{\prime} }}{M}_{{b}^{{\prime\prime} }}\omega {({{{\bf{q}}}})}_{s}\omega {({{{{\bf{q}}}}}^{{\prime} })}_{{s}^{{\prime} }}\omega {({{{{\bf{q}}}}}^{{\prime\prime} })}_{{s}^{{\prime\prime} }}}}\right | }^{2},$$

where $\bar{{\mathsf{N}}}{({{{\bf{q}}}})}_{s}={[\exp (\hslash \omega {({{{\bf{q}}}})}_{s}/{k}_{{{{\rm{B}}}}}T)-1]}^{-1}$ is the Bose-Einstein distribution, ${{\Delta }}({{{\bf{q}}}}+{{{{\bf{q}}}}}^{{\prime} }+{{{{\bf{q}}}}}^{{\prime\prime} })$ is the Kronecker delta (equal to 1 if ${{{\bf{q}}}}+{{{{\bf{q}}}}}^{{\prime} }+{{{{\bf{q}}}}}^{{\prime\prime} }$ is a reciprocal lattice vector, 0 otherwise), *δ* is the Dirac delta. The linewidth due to isotopic-mass disorder is determined exclusively by harmonic properties and concentration of isotopes^84^:

15 $$\hslash {{\Gamma} }_{{{{\rm{i}}}}}{({{{\bf{q}}}})}_{s}=\frac{\hslash \pi }{2{N}_{c}}{[\omega {({{{\bf{q}}}})}_{s}]}^{2}{\sum }_{{{{{\bf{q}}}}}^{{\prime} },{s}^{{\prime} }}\delta \left[\omega {({{{\bf{q}}}})}_{s}-\omega {({{{{\bf{q}}}}}^{{\prime} })}_{{s}^{{\prime} }}\right]\\ \times {\sum }_{b}{g}_{2}^{b}{\left| {\sum }_{\alpha }{{{\mathcal{E}}}}{({{{\bf{q}}}})}_{s,b\alpha }^{\star }{{{\mathcal{E}}}}{({{{{\bf{q}}}}}^{{\prime} })}_{{s}^{{\prime} },b\alpha }\right| }^{2},$$

where ${g}_{2}^{b}={\sum }_{i}{f}_{i,b}{\left(\frac{{M}_{b}-{m}_{i,b}}{{M}_{b}}\right)}^{2}$ describes the variance of the isotopic masses of atom *b* (*f*_*i*,*b*_ and *m*_*i*,*b*_ are the mole fraction and mass, respectively, of the *i*th isotope of atom *b*; *M*_*b*_ = ∑_*i*_*f*_*i*,*b*_*m*_*i*,*b*_ is the weighted average mass).

### Relation between Grüneisen parameter and third-order force constants

In the quasi-harmonic approximation, expanding the Hamiltonian to first order in applied strain allows one to evaluate the mode Grüneisen parameter^44,91^, which depends linearly on the third-order derivatives of the PES. In contrast, the thermal conductivity is, in the BTE relaxation-time approximation regime, inversely proportional to the anharmonic linewidths, and the latter depend on the square of the third-order derivatives of the PES. These distinct dependencies of conductivity and Grüneisen parameter on PES derivatives intuitively show why our SRME$\left[\{{{{\mathcal{K}}}}{({{{\bf{q}}}})}_{s}\}\right]$ and SRME$\left[\{{{{\mathcal{G}}}}{({{{\bf{q}}}})}_{s}\}\right]$ provide complementary information: errors in the PES derivatives that are very important for conductivity may have negligible impact on Grüneisen parameter, and vice-versa. Therefore, jointly analyzing thermal conductivity and Grüneisen parameters enables a more comprehensive benchmark of fMLP accuracy, helping in minimizing the risks of overlooking errors that could arise from subtle inaccuracies in modeling the physical PES.

### DFT reference and zero-shot mp-fMLP calculations

Computational details of the DFT calculations underlying the phononDB-PBE database can be found in refs. ^48,49^. Applying the same settings on the same set of materials, we computed the Grüneisen parameters by calculating the phonon band structures following the automated Grüneisen parameter workflow described in the Supplementary Information. These calculations employed the Perdew, Burke, and Ernzerhof (PBE) exchange-correlation functional^59^, consistent with the approach used for generating the MP dataset^46^, used to train mp-fMLPs.

Supplementary Fig. 6 shows that in wurtzite BeO, the zero-shot MACE-MP-0-L2 calculation has a computational cost that is more than 3 orders of magnitude lower than the conventional DFT calculation, and yields a conductivity compatible within 7% with DFT reference values (Fig. 2a). Importantly, we note that speedups much stronger than those highlighted in Supplementary Fig. 6 are expected in disordered materials without symmetries^51,92^. In fact, BeO has a simple unit cell containing just 4 atoms and with 12 crystal symmetries^93^, which allow one to reduce the number of displacements^94^. In contrast, in disordered materials symmetries are broken and consequently a larger number of finite-displacement calculations are needed. Moreover, standard DFT approaches scale with the cube of the number of atoms, while fMLPs have a linear scaling. Based on these considerations, we expect even stronger speedups in disordered materials^95–100^.

We note that the DFT parameters used to generate the phononDB-PBE database are similar but not always exactly equal to those used for DFT calculations in the MP database. In the regime of computational convergence, variations of the DFT parameters at fixed functional are expected to yield unimportant effects on the conductivity. In particular, ref. ^101^ shows that for the prototypical case of silicon, computing force constants using DFT parameters within the range of computationally converged values found in the literature produces conductivity variations that are within 7%, and also shows that in ideal cases varying DFT parameters within the domain of computational convergence should yield conductivity variations smaller than 2%. As such, the significant discrepancies shown in Fig. 3 are not expected to be caused by possible small differences in the DFT parameters.

The computational details on the mp-fMLPs can be found in the following references: M3GNet^21^, CHGNet^22^, MACE-MP-0^23^, SevenNet^24^, and ORB-v1-MPtraj^47^. M3GNet was used through the matgl^102^ (version 1.1.2) package with the M3GNet-MP-2021.2.8-PES model. The CHGNet version 0.3.8 was used. The MACE-MP-0 2024-01-07-mace-128-L2 model was run through LAMMPS^103^. SevenNet was used through the sevenn package (version 0.9.2) with the SevenNet-0_11July2024 model. ORB-v1-MPtraj was used through the orb-models package (version 0.3.0, commit d142854) with the ORB-v1-MPtraj-only model. The details on the violin plots presented in Fig. 4 can be found in ref. ^104^.

The ORB-v1-MPtraj, SevenNet, MACE, and CHGNet calculations were performed on an Nvidia Tesla A100 SXM4 80GB GPU, with the relaxation and force calculations for all 103 structures costing  ~ 3 GPU h per model. M3GNet was executed on AMD EPYC 7702 CPUs, with relaxation and force calculations costing around  ~ 30 CPU h. Thermal conductivity calculations for all 103 structures required about  ~ 50 CPU h per model, and similar resources were needed for evaluating thermal conductivity from the DFT force constants. Overall, the analysis of these five models required ~12 GPU h and ~330 CPU h.

### Details on fine-tuning

In this section we discuss how the size of the dataset and the number of fine-tuning gradient updates influence the training error for energies, forces, and stresses in LiBr. Results are shown for different fine-tuning datasets in Supplementary Fig. 7.

The three datasets used for fine-tuning contain DFT single-point calculations in 4 × 4 × 4 supercells of LiBr (the same size as used for the phonon calculation in the phononDB-PBE^48,49^ database) perturbed from equilibrium with atomic rattling and in some cases also volume variations from 0.25% to 5%. All DFT calculations are performed employing the Perdew, Burke, and Ernzerhof (PBE) exchange-correlation functional^59^, and the projector augmented wave (PAW) formalism for electronic minimization, implemented within the VASP (Vienna ab initio simulation package) code^105,106^. Following ref. ^49^, we use a 600 eV plane-wave-basis cutoff (20% higher than the default one), and the Brillouin zone is sampled at the Gamma point. Using these DFT settings, the LiBr structure in the phononDB-PBE database shows a residual negative stress of 960 Bar. Therefore, to make the most accurate possible comparison between fine-tuned MACE-MP-0 and reference DFT data, we relaxed the fine-tuned MACE-MP-0 structure exactly to the same residual stress present in the reference DFT-PBE data.

The MACE-MP-0 2024-01-07-mace-128-L2 model (https://github.com/ACEsuit/mace-mp/commits/mace_mp_0) was fine-tuned using the mace_run_train script in the main branch of (https://github.com/ACEsuit/mace). The training started from the last checkpoint of MACE-MP-0 L2. The three datasets discussed above were used to perform three independent trainings. For trainings on the 1-frame and 3-frame datasets we employed a batch size of 1, and for trainings on the 20-frame dataset we used a batch of size 4. Since our fine-tuning datasets were computed with the DFT-PBE functional, which was also employed to generate the MP database^46^ used to train MACE-MP-0, in the fine-tuning we kept the E0s fixed at their original MACE-MP-0 value. Representative examples of training scripts are available at: (https://github.com/MSimoncelli/fMLP_conductivity.git). The fine-tuning was performed on an Nvidia Tesla A100 SXM4 80GB GPU.

### Hybrid fMLP-DFT approach to include non-analytical correction term

**Fig. 6:**
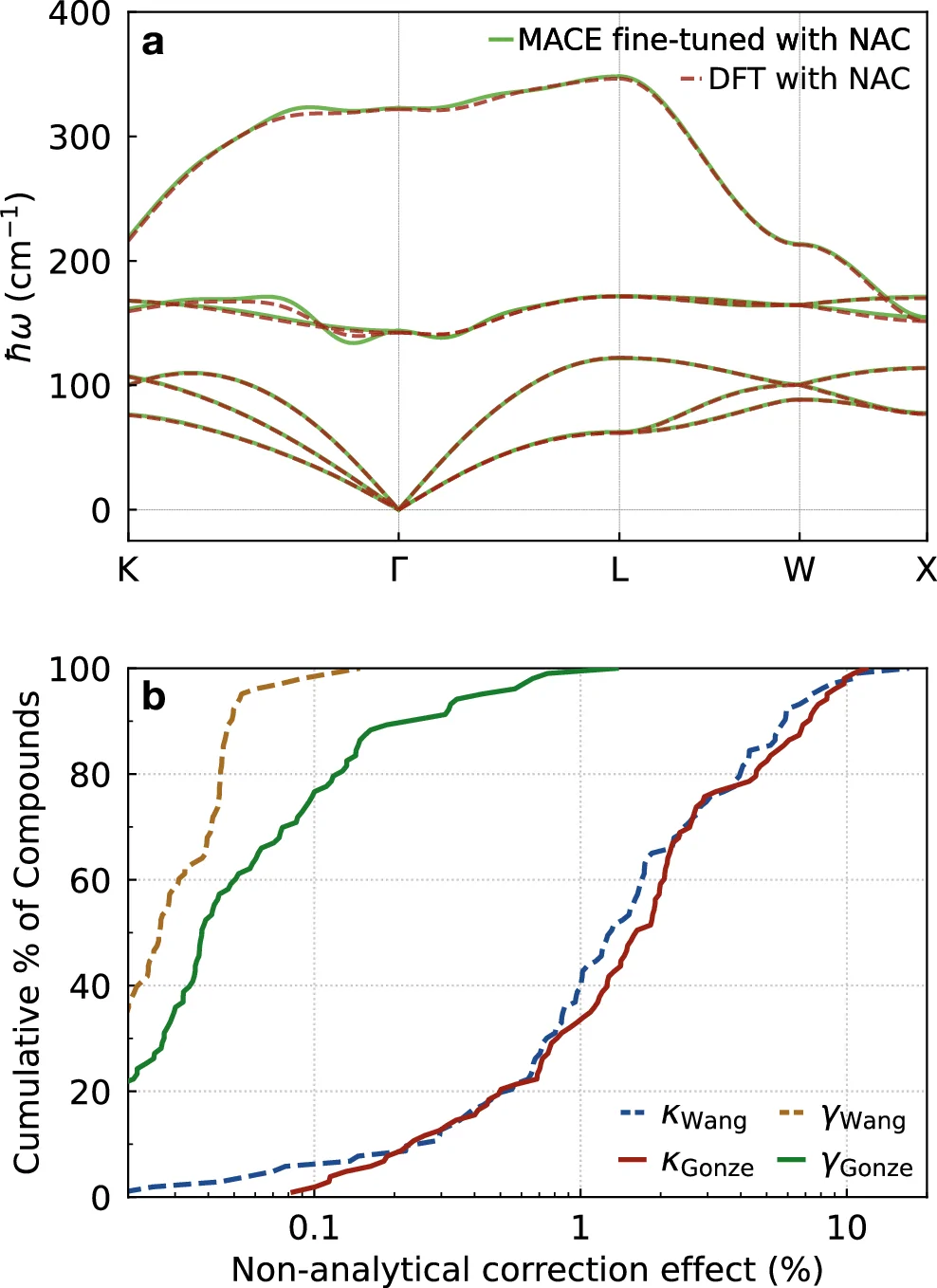
Effect of non-analytical correction on vibrational properties. **a** The hybrid fMLP-DFT approach shows good agreement with the reference band structure obtained from DFT. As an example, we used the MACE-MP-0 model fine-tuned on 3 frames over 600 gradient updates. **b** Cumulative distribution of the non-analytical correction term’s effect on conductivity and Grüneisen parameter, and comparison between the methods by Gonze and Lee^60^, and Wang et al. ^61^.

We propose a hybrid fMLP-DFT method to incorporate the NAC term^60^ computed from density functional perturbation theory (DFPT)^75,107^ into phonon band structures predicted by fMLPs. To limit the computational cost, we neglect changes in the Born effective charges that may originate from small structural differences between the DFT-relaxed and fMLP-relaxed primitive cell; the accuracy of this approximation will be discussed a posteriori. We demonstrate the effectiveness of this hybrid approach in LiBr, using the MACE-MP-0 potential fine-tuned on 3 frames for over 600 gradient updates, and combining it with DFT-quality Born effective charges from the phononDB-PBE database^49^. As shown in Fig. 6a, the hybrid approach with fine-tuned fMLPs and DFT Born effective charges is in good agreement with the DFT reference calculation, confirming the accuracy of our hybrid fMLP-DFT approach, and, a posteriori, the validity of the approximations performed therein.

### Influence of Born effective charges on phonon dispersion, conductivity, and Grüneisen parameter

The long-range NAC term to the phonon band structure can be described using two methodologies: (i) the one proposed by Gonze and Lee^60^ using density-functional perturbation theory in reciprocal space, and (ii) the mixed-space approach by Wang et al. ^61^. To evaluate the numerical difference between these two methods, we employed both of them to calculate the phonon band structures of LiBr, also checking how these two methods numerically converge with the size of the supercell used (all the other parameters were kept fixed and consistent with those of the phononDB-PBE dataset). Supplementary Fig. 8 shows that increasing the supercell size from 2 × 2 × 2 to 6 × 6 × 6 yields a strong effect in the band structures that do not include the NAC term (this was expected and is explained in detail in ref. ^61^), and a noticeable effect in the band structure computed accounting for NAC with Wang’s method. In contrast, Gonze’s method shows significantly smaller variations with respect to varying supercell size, displaying acceptable numerical convergence already in supercells of size 4 × 4 × 4. Therefore, in the plots of this work we account for NAC using the approach by Gonze and Lee^60^.

In Fig. 6b we show how thermal conductivity and Grüneisen parameter change when the NAC term is included or omitted, reporting the cumulative distribution of the relative variation for all the 103 materials analyzed. Overall, the materials analyzed do not show a significant dependence on whether the NAC term is included. In particular, the maximum variation for the Grüneisen parameter is 1.4% in rocksalt AgCl, while for conductivity it is 11.8% in wurtzite CdSe. The cumulative distribution of the relative NAC effect on conductivity and Grüneisen parameter is presented in Fig. 6b.

### Thermal expansion and bulk modulus within the quasi-harmonic approximation

To demonstrate that fine-tuned fMLPs can accurately describe not only thermal conductivity and Grüneisen parameter, but also other technologically relevant thermomechanical properties, we employed the fine-tuned fMLP to compute the volumetric thermal expansion and temperature-dependent bulk modulus of LiBr using free energy minimization within the quasi-harmonic approximation, as implemented in the phonopy package^66^. The capability of fine-tuned fMLPs to describe multiple different thermomechanical properties provides an advantage in terms of computational cost over the following established simulation approaches: (i) compressive sensing^64,108^, which requires separate DFT datasets at contracted and expanded unit-cell volumes to describe both conductivity and Grüneisen parameter; (ii) traditional non-foundation machine-learning potentials, which rely on much larger training datasets to reach accuracy comparable to fine-tuned fMLPs^92^.

**Fig. 7:**
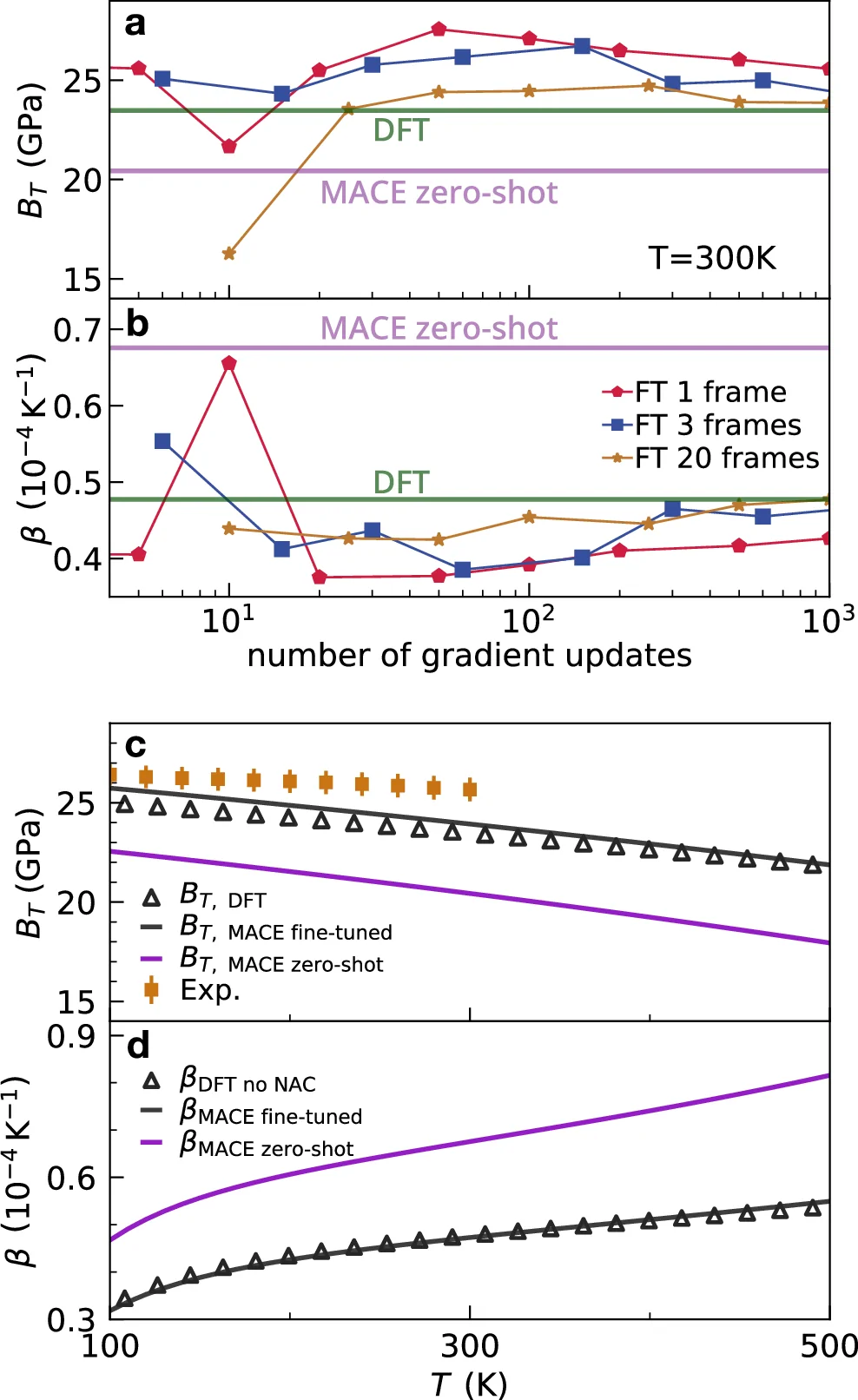
Volumetric thermal expansion and temperature-dependent bulk modulus of LiBr within the quasi-harmonic approximation. **a** Temperature-dependent bulk modulus and **b** volumetric thermal expansion coefficient of LiBr computed using MACE-MP-0 fine-tuned on different numbers of frames up to 1000 gradient updates, with DFT-PBE (green) and MACE-MP-0 zero-shot (purple) shown for comparison. The temperature dependence of the quantities is shown in panel **c**, **d** along with experimental bulk modulus values^109^.

The DFT reference data were generated with the same computational settings as the fine-tuning dataset, applying volume variations of  ± 1%,  ± 2%,  ± 3%,  ± 4%, and  ± 5%, alongside 0.03 Å displacements for interatomic force constants and the default Vinet equation of state. The MACE-MP-0 model was evaluated both in a zero-shot setting and after fine-tuning on three frames over 1000 gradient updates. Fig. 7 presents the resulting volumetric thermal expansion coefficients and temperature-dependent bulk modulus compared to the DFT reference. The fine-tuned MACE-MP-0 model closely reproduces the DFT results for thermal expansion within 1.5% and for bulk modulus within 3% across the full temperature range, whereas the zero-shot model significantly overestimates the thermal expansion coefficient and underestimates the bulk modulus. Experimental bulk modulus values^109^ are plotted for comparison. We note that the bulk modulus exhibits a clear temperature dependence over the plotted range, emphasizing that thermal expansion cannot be divided into independent single-phonon contributions. This is due to the temperature dependence of the bulk modulus^44^ and of the phonon-dependent terms appearing in the expression for the volumetric thermal expansion, $\beta=\frac{\upgamma C}{{B}_{T}}$, as discussed in the main text.

### Effect of finite-difference displacement amplitude on conductivity and Grüneisen parameter

**Fig. 8:**
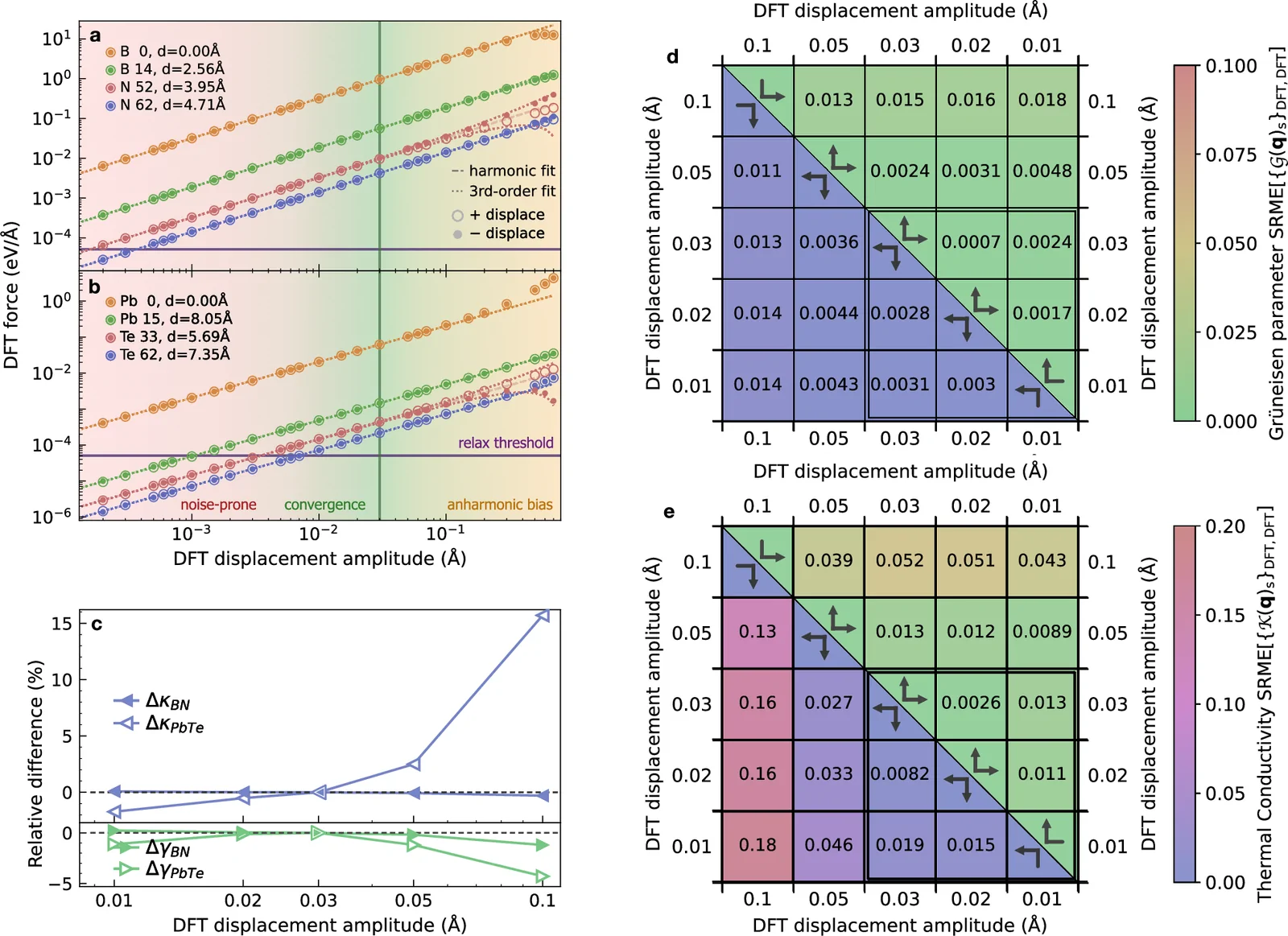
Selecting a finite-difference displacement amplitude for accurate DFT thermomechanical calculations. **a**, **b** DFT forces on different atoms as a function of the displacement amplitude of the index 0 atom in 2 × 2 × 2 supercells of zincblende BN (**a**) and rocksalt PbTe (**b**). Open and filled markers denote positive and negative displacements, respectively; the absolute value of the *x* component of the force is plotted. The different colored lines and markers correspond to forces exerted on different atoms (the legend provides the index, the chemical species, and the minimum-image-convention distance from the displaced atom). The linear behavior in the force (dashed line) corresponds to the harmonic dependence of the energy on the displacement. The vertical green line represents the default finite-difference (FD) amplitude 0.03 Å, while the horizontal purple line indicates the possible residual force magnitude corresponding to a relaxation threshold. The increasing separation of the filled and open markers represents third-order anharmonicity (dotted line), while deviations in a common direction show fourth-order anharmonicity. The shaded regions indicate the noise-prone regime (red), the converged FD regime (green), and the regime dominated by higher-order anharmonic bias (orange). **c** Relative deviations in the thermal conductivity, *κ*, and Grüneisen parameter, γ, calculated using each displacement amplitude with respect to the values obtained using the default 0.03 Å amplitude, for BN and PbTe. **d**, **e** DFT–DFT symmetric relative mean errors (SRME) of the mode-resolved contributions to *κ* (lower triangles) and γ (upper triangles) in BN (**d**) and PbTe (**e**). Each entry compares calculations performed using the corresponding pair of finite-difference displacement amplitudes. Diagonal entries vanish by construction. Separate color scales are used for the conductivity and Grüneisen-parameter SRMEs.

We use the finite-difference (FD) method to compute force constants, and in this section we discuss how the displacement amplitude influences conductivity and Grüneisen parameter. In DFT, the FD displacements have to be chosen within a physically and numerically sensible range, ensuring that force perturbations are numerically large enough to be above the DFT noise floor and physically small enough to be treated as perturbations. To balance these effects, a displacement amplitude of 0.03 Å is employed in standard Born-Oppenheimer DFT calculations^48,49^, and more generally a finite displacement within the range 0.005–0.05 Å is chosen as a default in other works^90^. In most DFT simulations, varying the FD amplitude within this range produces minor differences in the macroscopic thermomechanical properties, since the DFT PES is generally smooth (more on this later). An accurate fMLP is expected to reproduce the DFT PES, and hence to display the same weak or negligible dependence between FD amplitude and thermomechanical properties. However, due to suboptimal architectural choices or limited training data, fMLPs can encode a PES significantly less smooth than the DFT one^72,110^; in these cases, DFT and fMLPs can display very different relations between FD amplitude and derived thermomechanical quantities. To analyze these effects, we first illustrate the behavior of the DFT PES near the equilibrium of two example materials, zincblende BN and rocksalt PbTe, in Fig. 8a, b.

The atom with index 0 is displaced by different amplitudes along the *x*-direction, and the corresponding forces are computed with DFT in a 2 × 2 × 2 supercell. We obtain the dependence of the atomic forces on the displacement amplitude, which is plotted for some selected atoms. The details of these selected atoms are provided in the legend, including their index, chemical species, and minimum-image-convention distance from the displaced atom. These forces are used in the FD method to compute the force-constant elements representing the derivatives of the PES with respect to these displacements. In particular, the harmonic, second-order force constants are obtained as the first numerical derivative of the force with respect to this displacement, while the third-order force constants are calculated as second derivatives. We plot the linear fit to the force (dashed), and the quadratic fit (dotted), which are obtained from the harmonic and the third-order force constant elements, respectively, computed with a displacement amplitude of 0.03 Å. This correctly explains the increasing separation between the positive (open markers) and negative (filled markers) displacement directions of the red markers on the right-hand side of Fig. 8b, corresponding to large third-order force constants on the logarithmic scale. However, we observe an offset in the same direction in the case of the orange markers, which is caused by the fourth-order contribution becoming significant at large displacement amplitudes. This leads to errors in the finite-difference force constants computed at these large amplitudes. We illustrate this anharmonicity-biased region with the orange shaded area. In the opposite low-displacement regime (red shaded area), some forces can be lower than the relaxation force threshold, which we represent by 10^−6^ Ha/Bohr  = 5.14 × 10^−5^ eV/Å, similar to the threshold applied in the Materials Project phonon dataset^111^. This can be significant for very weak force constant elements even for the harmonic term, and much more pronounced for the third-order term. In this region of the displacement amplitude, we expect less reliable results and possible influence from noise—in particular, even if the current plots are smooth for small displacements, this might be due to their crystal symmetries, and more generally in complex glassy materials it is not reliable to compute FD quantities from force differences that are comparable to the DFT force-convergence threshold. Between these two regimes, converged force constants can be calculated, as shown by the green shaded area.

To analyze the effect of the displacement amplitude not only on the force constants, but also on the derived thermomechanical quantities, we have computed the thermal conductivity and the Grüneisen parameter with DFT at 0.1, 0.05, 0.03, 0.02, and 0.01 Å displacement amplitudes for zincblende BN and rocksalt PbTe. To have a comprehensive picture and resolve possible error cancellation, we compare both the macroscopic quantities and the microscopic mode-resolved contributions. Conveniently, the SRME is applicable in this case, as the metric is symmetric and allows the reference and estimate to be exchanged. Therefore, to compare the derived quantities obtained with different displacement amplitudes, we use a DFT-DFT SRME, defined as

16 $$	 {{\rm{SRME}}}\,\left[{\{{{{\mathcal{K}}}}{({{{\bf{q}}}})}_{s}\}}_{{{{\rm{DFT,DFT}}}}}\right]=\\ 	 \qquad \qquad \frac{2}{{N}_{c}{{{\mathcal{V}}}}}\frac{{\sum }_{{{{\bf{q}}}}s}\left\vert {{{{\mathcal{K}}}}}_{{{{\rm{DFT1}}}}}{({{{\bf{q}}}})}_{s}-{{{{\mathcal{K}}}}}_{{{{\rm{DFT2}}}}}{({{{\bf{q}}}})}_{s}\right\vert }{{\kappa }_{{{{\rm{DFT1}}}}}+{\kappa }_{{{{\rm{DFT2}}}}}},$$

where DFT1 and DFT2 represent two DFT calculations with different displacement amplitudes. We define the $\,{{\rm{SRME}}}\,\left[{\{{{{\mathcal{G}}}}{({{{\bf{q}}}})}_{s}\}}_{{{{\rm{DFT,DFT}}}}}\right]$ similarly. In this way, we compute the SRME values between the mode-resolved thermal conductivity and Grüneisen parameter obtained with DFT using different displacement amplitudes. The resulting SRME matrices of both quantities are symmetric with vanishing diagonals (the diagonal elements trivially correspond to comparing a DFT calculation with itself). Therefore, we plot the SRME matrices with the thermal-conductivity values in the lower triangular part and the Grüneisen-parameter values in the upper triangular part for BN and PbTe in Fig. 8d, e respectively. Shared colormaps are used, with upper limits chosen to represent large deviations comparable to the performance of recent fMLPs^72,110^. Because the SRME for the mode-resolved Grüneisen parameter is consistently about a factor of two lower than that for the thermal conductivity, we visualize them using different colormaps. The changes in BN are minor, while they can be significant in PbTe, due to strong higher-order anharmonicity^112^. For PbTe, the deviations are large at 0.05 Å and 0.1 Å, while they are smaller between 0.01 Å and 0.03 Å, which we identify as the converged region and highlight with a double border to guide the eye.

The macroscopic quantities are also displayed in Fig. 8c, showing the relative difference in the total thermal conductivity and Grüneisen parameter at 300 K as a function of the displacement amplitudes. The same trends are observed: the results at 0.05 Å and 0.1 Å amplitudes in PbTe deviate significantly, whereas the lower amplitudes are converged, as in the case of BN. This confirms that the effect of displacement amplitude on the force constants and the derived thermomechanical quantities is material dependent, and that the displacement amplitude used here lies within the converged range.

The displacement regimes identified above apply to DFT calculations. When assessing the utility of fMLPs for computing these thermomechanical quantities, we need to consider noise and possible architecture-dependent inaccuracies in the PES described by the fMLP, which can amplify the influence of the FD amplitude on the thermomechanical properties compared to DFT. Since the DFT reference data used here are available with 0.03 Å displacement amplitudes, and an ideal fMLP is expected to reproduce the DFT PES, benchmarks involving comparison between DFT and fMLP should use the original 0.03 Å displacement amplitude of the reference datasets.

## Supplementary information

Supplementary Information

Transparent Peer Review file

## Data Availability

The Matbench Discovery results are available at (https://matbench-discovery.materialsproject.org/). The phononDB-PBE dataset including the displacements generated by phono3py and the corresponding force sets are available at (https://github.com/atztogo/phonondb^48,49^). The phonon mode resolved thermal conductivity and the fine-tuning data generated in this study have been deposited in the Materials Cloud Archive^113^ database at (https://doi.org/10.24435/materialscloud:2d-4b).

## Code availability

The phono3py and phonopy packages are available at https://github.com/phonopy/; the ase package is available at https://gitlab.com/ase/ase; the spglib package is available at https://github.com/spglib/spglib. matgl containing the M3GNet model is available at https://github.com/materialsvirtuallab/matgl; the CHGNet model and package are available at https://github.com/CederGroupHub/chgnet; the SevenNet model and package are available at https://github.com/MDIL-SNU/SevenNet; the ORB-v1-MPtraj model and package are available at https://github.com/orbital-materials/orb-models. The MACE used through LAMMPS^103^ is available at https://github.com/ACEsuit/lammps, and the MACE-MP-0 model is available at https://github.com/ACEsuit/mace-mp. The Hiphive package is available at https://gitlab.com/materials-modeling/hiphive. The source code used to reproduce the results presented in this work is publicly available. The k_SRME code, which enables the thermal conductivity benchmarks, is accessible at https://github.com/MPA2suite/k_SRME^114^. The gamma_SRME code, used for computing Grüneisen parameter benchmarks, is available at https://github.com/MPA2suite/gamma_SRME^115^.
