# Supplementary material for: Thermal conductivity predictions with foundation atomistic models
Source: Nat Commun. 2026 Sep 5;17:10566. doi: 10.1038/s41467-026-76391-w (PMC13642343; doi:10.1038/s41467-026-76391-w)
Supplement: Supplementary file 1 [file 41467_2026_76391_MOESM1_ESM.pdf]

Supplementary Information to 'Thermal Conductivity Predictions with Foundation Atomistic Models'

Balázs Póta,[1] Paramvir Ahlawat,[1] Gábor Csányi,[2,3] and Michele Simoncelli[1,4]

[1] *Theory of Condensed Matter Group of the Cavendish Laboratory, University of Cambridge, Cambridge, UK*
[2] *Engineering Laboratory, University of Cambridge, Cambridge, UK*
[3] *Max Planck Institute for Polymer Research, Mainz, Germany*
[4] *Department of Applied Physics and Applied Mathematics, Columbia University, New York (USA)*

Supplementary Note

Failures of semiclassical Boltzmann transport

As anticipated in the main text, our automated framework can be used to identify materials in which the semiclassical particle-like BTE fails. This happens when particle-like propagation mechanisms — described by both the BTE and WTE[1–3] and determining κ_P in Eq. (1) of the main text — do not dominate over wave-like tunneling mechanisms — missing from the BTE but described by the WTE and determining κ_C in Eq. (1) of the main text. Failures of the BTE have been discussed, e.g., in strongly anharmonic complex crystals for energy harvesting[4–16], thermal barrier coatings[17–20], as well as in several disordered functional materials[21–26]. Our automated framework computes the total Wigner thermal conductivity $\kappa_P + \kappa_C$[27], and therefore can be used to find materials that violate semiclassical Boltzmann thermal transport. In Fig. 1 we show that among the 103 compounds analyzed at 300 K, the BTE fails in materials with low thermal conductivity ($\kappa_{\mathrm{TOT}} \lesssim 2$ W/mK), such as AgBr and AgCl, since the wave-like tunneling conductivity becomes comparable to the particle-like propagation conductivity. We also note that in simple crystals with large conductivity, the BTE is accurate because the particle-like propagation conductivity dominates over the wave-like tunneling conductivity.

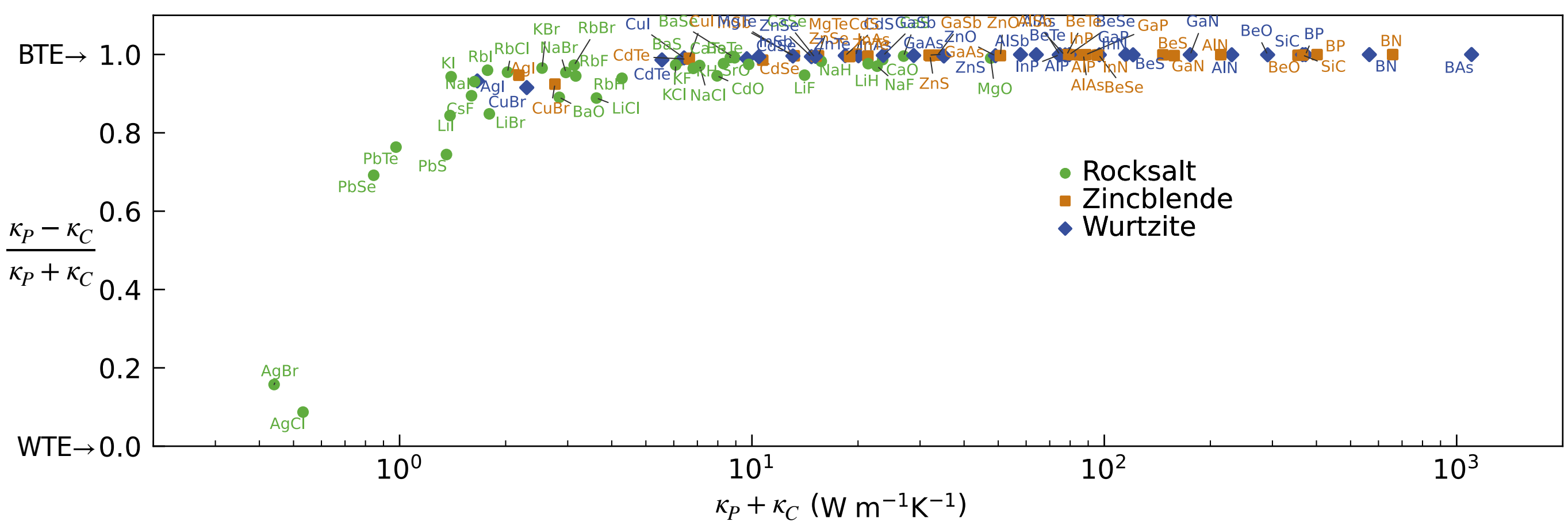

SUPPLEMENTARY FIGURE 1. **Failures of the semiclassical Boltzmann transport equation (BTE) in low-conductivity binary crystals** with rocksalt (green), zincblende (orange), and wurtzite (blue) structures. These occur when the conductivity described by the BTE in terms of particle-like propagation of phonons (κ_P) does not dominate over the coherence conductivity due to phonons' wave-like tunneling (κ_C). The WTE[27] accounts for both κ_P and κ_C, and failures of semiclassical Boltzmann transport are highlighted by a relative strength of particle-wave transport $(\kappa_P - \kappa_C)/(\kappa_P + \kappa_C)$ appreciably smaller than one (more intense red background, as in Fig. 1 of the main text). These conductivities were obtained from the force constants in the phononDB-PBE dataset[28,29].

Symmetric Relative Mean Error on harmonic and anharmonic vibrational properties

The analyses reported in the main text discuss SRME$[\{\mathcal{K}(\mathbf{q})_s\}]$ as a descriptor of the accuracy of fMLPs in describing microscopic harmonic and anharmonic properties. In this section we provide tools to resolve whether a large SRME$[\{\mathcal{K}(\mathbf{q})_s\}]$ originates from large errors in microscopic harmonic properties, large errors in anharmonic properties, or both. To this end, we employ the expression for the microscopic, single-phonon contributions to the thermal conductivity (see terms inside the square brackets in Eq. (1) of the main text) to define descriptors that quantify the accuracy with which harmonic and anharmonic properties are predicted. In particular, we note that the conductivity

contribution of a phonon $(\mathbf{q})_s$ is a function of: (i) all the phonon frequencies $\{\omega(\mathbf{q})_{s'}\}$ at a fixed wavevector $\mathbf{q}$ and variable mode $s'=1,\ldots,N_{\rm s}$ ($N_{\rm s}$ is the number of phonon bands, equal to 3 times the number of atoms in the crystal's unit cell[30]); (ii) all velocity-operator elements $\{\mathbf{v}(\mathbf{q})_{s,s'}\}$ at fixed $\mathbf{q}$, s and variable s'; (iii) all isotopic linewidths at fixed $\mathbf{q}$ and variable s', $\{\Gamma_{\rm i}(\mathbf{q})_{s'}\}$, which depend solely on harmonic properties, see Eq. (15) of the main text; (iv) the anharmonic linewidths $\{\Gamma_{\rm a}(\mathbf{q})_{s'}\}$ at fixed $\mathbf{q}$ and variable s'. This can be summarized using the following notation: $\mathcal{K}(\mathbf{q})_s=\mathcal{K}(\mathbf{q})_s[\{\omega(\mathbf{q})_{s'}\},\{\mathbf{v}(\mathbf{q})_{s,s'}\},\{\Gamma_{\rm i}(\mathbf{q})_{s'}\},\{\Gamma_{\rm a}(\mathbf{q})_{s'}\}]$ where the curly brackets denote the set of values of a certain quantity over all the bands at fixed $\mathbf{q}$ (e.g., $\{\omega(\mathbf{q})_{s'}\}=\{\omega(\mathbf{q})_1,\omega(\mathbf{q})_2,\ldots,\omega(\mathbf{q})_{N_s}\}$).

To quantify the impact of errors in the harmonic (har) and anharmonic (anh) properties on the single-phonon conductivity contributions, we define the Symmetric Relative Mean Error (SRME) on these properties as follows:

$$\text{SRME[har]}=2\frac{\sum_{\mathbf{q}s}\left|\mathcal{K}(\mathbf{q})_s\left[\{\omega_{\rm fMLP}(\mathbf{q})_{s'}\},\{\mathbf{v}_{\rm fMLP}(\mathbf{q})_{s,s'}\},\{\Gamma_{\rm i,fMLP}(\mathbf{q})_{s'}\},\{\Gamma_{\rm a,DFT}(\mathbf{q})_{s'}\}\right]-\mathcal{K}_{\rm DFT}(\mathbf{q})_s\right|}{\sum_{\mathbf{q}s}\left(\mathcal{K}(\mathbf{q})_s\left[\{\omega_{\rm fMLP}(\mathbf{q})_{s'}\},\{\mathbf{v}_{\rm fMLP}(\mathbf{q})_{s,s'}\},\{\Gamma_{\rm i,fMLP}(\mathbf{q})_{s'}\},\{\Gamma_{\rm a,DFT}(\mathbf{q})_{s'}\}\right]+\mathcal{K}_{\rm DFT}(\mathbf{q})_s\right)}, \tag{1}$$

$$\text{SRME[anh]}=2\frac{\sum_{\mathbf{q}s}\left|\mathcal{K}(\mathbf{q})_s\left[\{\omega_{\rm DFT}(\mathbf{q})_{s'}\},\{\mathbf{v}_{\rm DFT}(\mathbf{q})_{s,s'}\},\{\Gamma_{\rm i,DFT}(\mathbf{q})_{s'}\},\{\Gamma_{\rm a,fMLP}(\mathbf{q})_{s'}\}\right]-\mathcal{K}_{\rm DFT}(\mathbf{q})_s\right|}{\sum_{\mathbf{q}s}\left(\mathcal{K}(\mathbf{q})_s\left[\{\omega_{\rm DFT}(\mathbf{q})_{s'}\},\{\mathbf{v}_{\rm DFT}(\mathbf{q})_{s,s'}\},\{\Gamma_{\rm i,DFT}(\mathbf{q})_{s'}\},\{\Gamma_{\rm a,fMLP}(\mathbf{q})_{s'}\}\right]+\mathcal{K}_{\rm DFT}(\mathbf{q})_s\right)}, \tag{2}$$

where we have used the shorthand notation $\mathcal{K}_{\rm DFT}(\mathbf{q})_s=\mathcal{K}_{\rm DFT}(\mathbf{q})_s\left[\{\omega_{\rm DFT}(\mathbf{q})_{s'}\},\{\mathbf{v}_{\rm DFT}(\mathbf{q})_{s,s'}\},\{\Gamma_{\rm i,DFT}(\mathbf{q})_{s'}\},\{\Gamma_{\rm a,DFT}(\mathbf{q})_{s'}\}\right]$. Intuitively, SRME[har] (1) is large when harmonic vibrational properties ($\omega(\mathbf{q})_s$, or $\mathbf{v}(\mathbf{q})_{s,s'}$, or $\Gamma_{\rm i}(\mathbf{q})_s$) differ between DFT and fMLP, while SRME[anh] (2) is large when the anharmonic linewidths ($\Gamma_{\rm a}(\mathbf{q})_s$) differ between DFT and fMLP. We show in Fig. 2 that small SRME[har] and small SRME[anh] (e.g., as in BeO) imply a small SRE[κ]. Fig. 2 also shows that large SRME[har] and large SRME[anh] often translate into large SRE[κ] (e.g., LiBr); however, there are also cases (e.g., BeTe) in which large SRME[har] and large SRME[anh] can compensate each other and result in a small SRE[κ]. This confirms that SRE[κ] is not a reliable descriptor for the capability of fMLPs to capture the harmonic and anharmonic physics underlying heat conduction. In contrast, having both small SRME[har] and small SRME[anh] is a sufficient condition to accurately capture harmonic and anharmonic vibrational properties, as well as conductivity.

Overall, these tests illustrate the importance of benchmarking the accuracy of fMLPs in predicting both microscopic

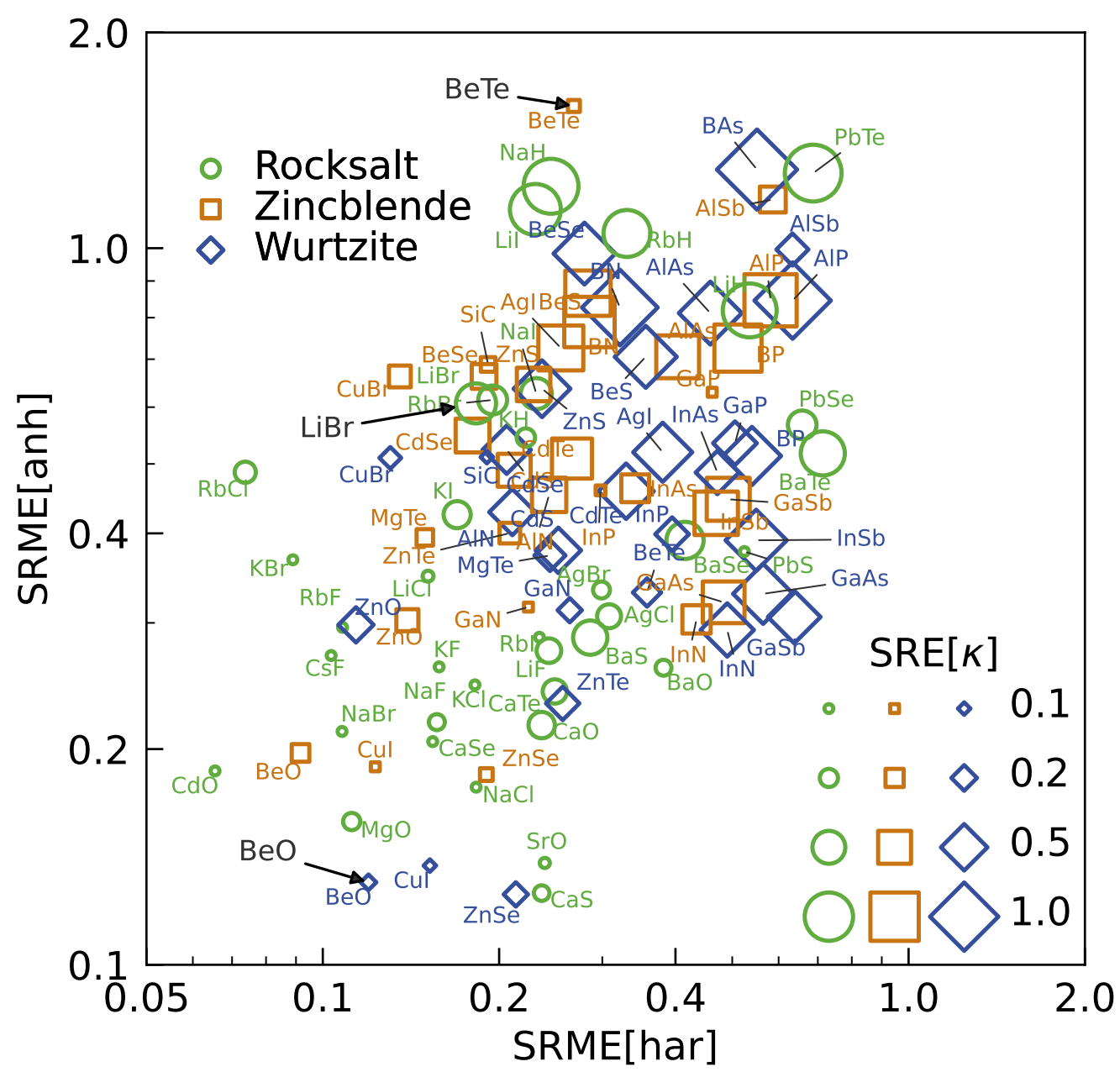

SUPPLEMENTARY FIGURE 2. **Relation between harmonic and anharmonic errors, and thermal conductivity,** calculated for 103 compounds using Eq. (1) and Eq. (2) considering density functional theory (DFT) and MACE-MP-0[31]. The area of each marker is proportional to the symmetric relative error in conductivity, SRE[κ], Eq. (3) of the main text. We highlight how the symmetric relative mean error, SRME[har] (Eq. (1)), SRME[anh] (Eq. (2)), and SRE[κ] are all low (large) in BeO (LiBr); in contrast, BeTe displays large SRME[har], large SRME[anh], but low SRE[κ] due to compensation of errors.

harmonic and anharmonic vibrational properties, further motivating the introduction of the SRME[$\{\mathcal{K}(\mathbf{q})_s\}$].

Influence of isotopic scattering on conductivity

To analyze the influence of isotope scattering on the conductivity, we compare thermal conductivity values with and without the linewidths from isotope mass disorder (Eq. (15) of the main text). The results are summarized in Table I. In wurtzite BeO and rocksalt LiBr, the impact of isotope scattering is minimal or small, respectively. However, in zincblende BeTe, isotopic and anharmonic linewidths are comparable (Fig. 2**b** of the main text), and therefore both affect the conductivity. In this material, when isotope scattering is included, the MACE-MP-0 errors in the linewidths relative to the DFT reference partially compensate across different regions of the spectrum. When isotope scattering is omitted, this compensation is reduced, and MACE-MP-0 therefore shows a significantly larger error in the thermal conductivity.

	wurtzite BeO		zincblende BeTe		rocksalt LiBr	
	DFT	MACE	DFT	MACE	DFT	MACE
with $\Gamma_{\rm i}(\mathbf{q})_s$	286.391	259.443	78.246	69.138	1.789	0.948
without $\Gamma_{\rm i}(\mathbf{q})_s$	291.963	263.956	289.374	402.030	1.803	0.951

TABLE I. **Influence of isotopic scattering on the thermal conductivity at 300 K** for BeO, BeTe, and LiBr.

Sensitivity of specific heat to fMLP accuracy

To show that inaccuracies in the PES described by SOTA fMLPs have a small (almost negligible) impact on the specific heat, we consider as paradigmatic cases the materials discussed in Fig. 2 of the main text, namely wurtzite BeO, zincblende BeTe, and rocksalt LiBr. Fig. 3 presents a comparison of the specific heat computed from DFT and zero-shot MACE-MP-0 for these materials, illustrating that specific heat — and more broadly, phonon spectrum–derived observables — exhibit reduced sensitivity to phonon band structure and hence to the accuracy of the model. This can be intuitively understood from the expression for the specific heat:

$$C = \frac{1}{VN_c}\sum_{\mathbf{q}s}\frac{\hbar^2\omega^2(\mathbf{q})_s}{k_{\rm B}T^2}\bar{N}(\mathbf{q})_s\left[\bar{N}(\mathbf{q})_s{+}1\right] \tag{3}$$

where $\mathbf{q}$ and s are the phonon wavevector and mode index, V is the unit cell volume and N_c is the number of wavevectors appearing in the sum; $\omega(\mathbf{q})_s$ is the phonon energy, and $\bar{N}(\mathbf{q})_s$=$[\exp(\hbar\omega(\mathbf{q})_s/k_{\rm B}T){-}1]^{-1}$ is the Bose-Einstein distribution at temperature T. Eq. (3) shows that at finite T the quantum specific heat is more sensitive to low-energy (acoustic) phonon modes and becomes progressively less sensitive to modes with increasingly higher frequency (e.g., optical phonon modes). SOTA fMLPs describe low-energy phonons relatively well and suffer from a systematic softening of optical phonons[32], and because the latter inaccuracy involves predominantly high-energy phonons that are unimportant for the specific heat, the specific heat is reasonably well described by SOTA fMLPs already at the zero-shot level.

Automated Wigner conductivity workflow

The workflow, outlined in Fig. 4, includes structure relaxation, force constant computation, thermal conductivity evaluation, and analysis. To automate the calculation of interatomic forces, we developed an interface between the packages `ase`[33] (version 3.23.0) and `phono3py`[28,34] (version 3.2.0). Atomic positions and unit-cell lattice parameters were simultaneously relaxed to minimize energy, forces, and stresses. We note that the determination of irreducible $\mathbf{q}$ points needed for thermal conductivity calculations depends on the crystal symmetry, and to evaluate SRME[$\{\mathcal{K}(\mathbf{q})_s\}$] (Eq. (4) of the main text) it is essential to compare structures that have the same crystal symmetry when studied using DFT or fMLPs.

We constructed a two-stage relaxation protocol, employing the FrechetCellFilter and FIRE algorithms in both stages to simultaneously optimize atomic positions and cell parameters. We used a force convergence threshold of 10^{-4} eV/Å and a maximum number of steps of 300. During the first stage, symmetries are explicitly enforced as constraints, whereas in the second stage the constraint on crystal symmetry is removed to test whether the fMLP correctly preserves the physical crystal symmetry. If the symmetry is preserved, the final relaxed structure is used. If symmetry is broken, the structure from the first stage, with enforced symmetry, is retained. We detect symmetries using the `spglib` package[35] with the default precision parameters. As discussed in Fig. 4**c** of the main

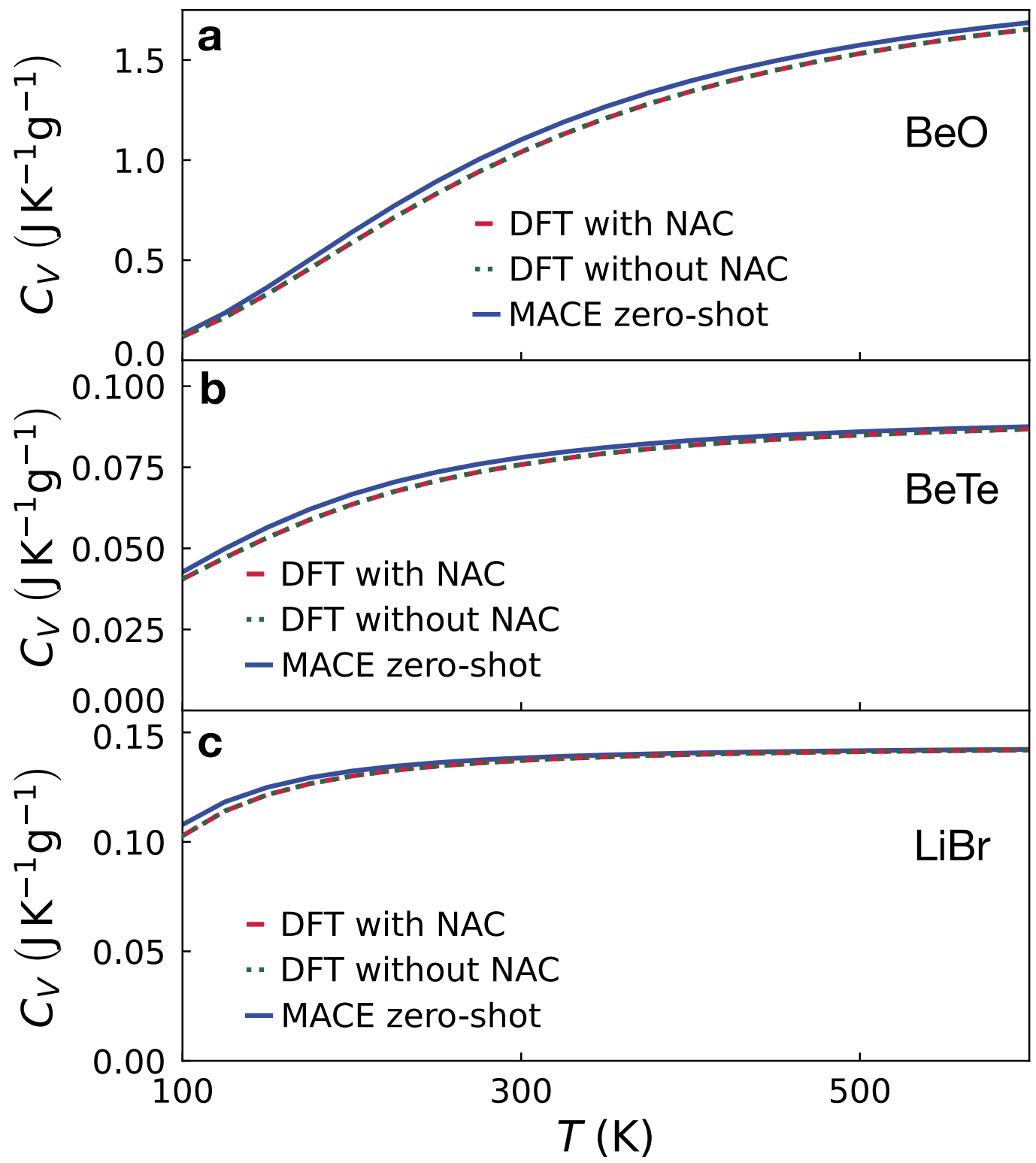

SUPPLEMENTARY FIGURE 3. **Specific heat of a) wurtzite BeO, b) zincblende BeTe, and c) rocksalt LiBr.** The zero-shot MACE-MP-0 predictions are in good agreement with density functional theory (DFT), confirming that optical phonon mode softening has a small (unimportant) effect on the specific heat.

text, in some cases retaining the symmetric structure yielded structural instabilities (imaginary phonon frequencies), which disappeared when the structure was allowed to break the symmetry and relax. While these structurally stable and broken-symmetry structures are not useful for evaluating SRME$[\{\mathcal{K}(\mathbf{q})_s\}]$, they provide information about shortcomings of fMLPs that could be addressed in future studies or used as a benchmark metric for accuracy in the description of crystal structures.

In the supercell force-constant calculations, we used the same parameters as in the DFT reference data[28,29].

Thermal conductivities were computed using `phono3py` following Refs.[28,34]. A $19 \times 19 \times 19$ **q**-mesh was used for rocksalt and zincblende structures, and a $19 \times 19 \times 15$ **q**-mesh for wurtzite structures. The collision operator was computed using the tetrahedron method. For Fig. 2 of the main text, linewidths were calculated on a $9 \times 9 \times 9$ **q**-mesh, for graphical clarity. We used version 3 of `phono3py` for anharmonic linewidth calculations and employed the Gonze method[36] for the NAC term, as implemented in the `phonopy`[34,37] package (version 2.26.6). For consistency with the phononDB-PBE dataset, in our data release we also include the conductivity results computed with the Wang method[38]. When structures with imaginary phonon frequencies were found with mp-fMLPs, these were discarded from subsequent thermal-conductivity analysis.

Automated Grüneisen parameter workflow

The workflow for computing Grüneisen parameters consists of structure relaxation, force constant computation for three structures (equilibrium volume, volume expanded 1%, and volume contracted 1%), Grüneisen parameter calculation, and analysis. The structure relaxation is carried out using the same two-stage protocol described in the previous section. To generate the configurations at volumes expanded or contracted by 1%, we scale the unit cells while keeping the relative atomic positions fixed, then relax the structures using the same procedure, with the cell volume held fixed at each step. Harmonic force constants for the three volumes are computed using 0.03 Å displacements and the same supercells used in the conductivity workflow. The `phonopy` package[34,37] is then used to calculate the mode Grüneisen parameters over the same **q**-mesh as in the conductivity calculations.

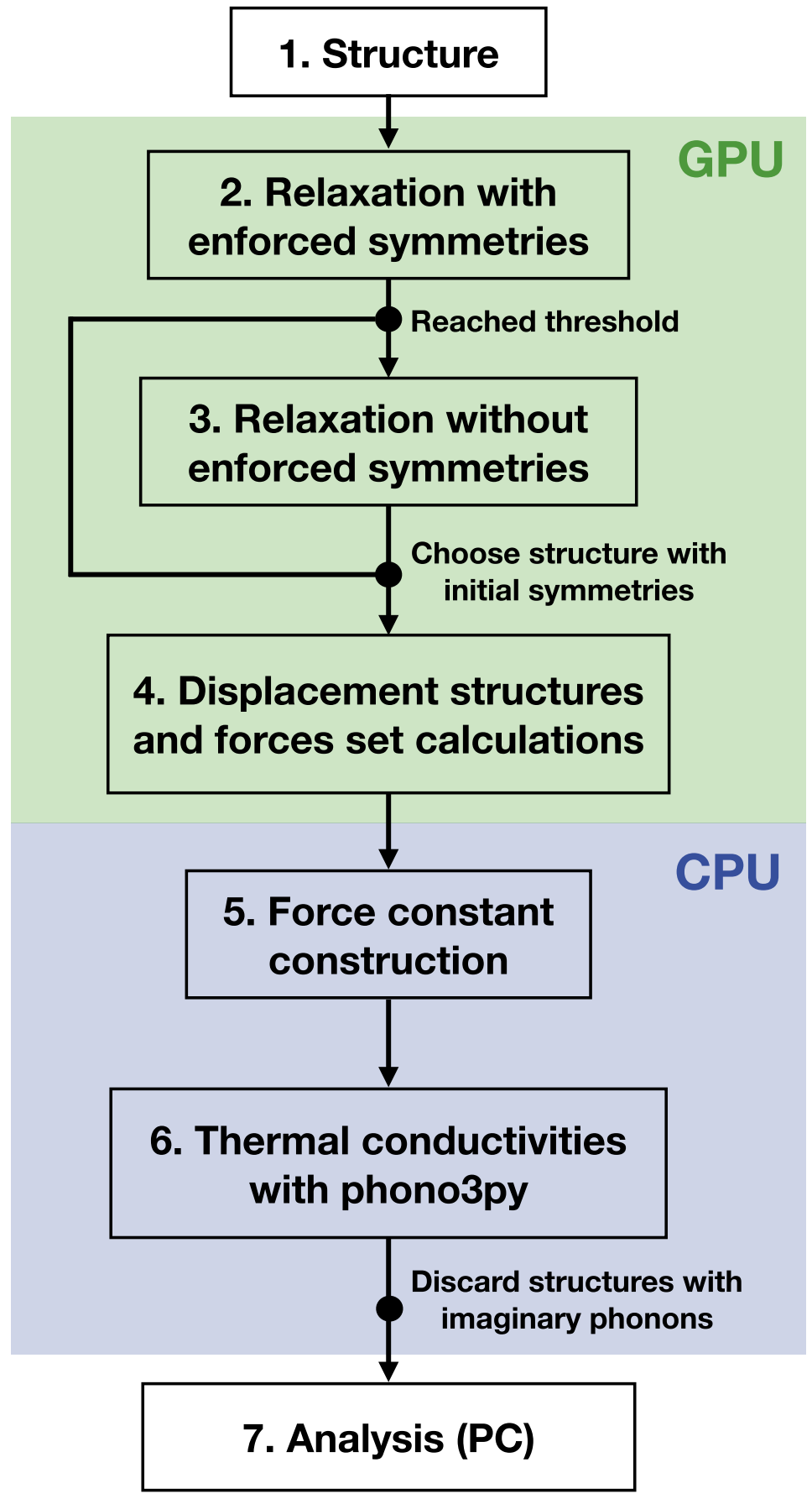

SUPPLEMENTARY FIGURE 4. **Automated Wigner Conductivity Workflow.** The first part of the workflow, involving force evaluation, is performed on a GPU. The input structures undergo relaxation in two stages. In the first stage, initial symmetries are enforced while simultaneously relaxing atomic positions and cell parameters. In the second stage, the structure is further relaxed by removing the symmetry constraint. If the symmetry group of the final structure changes, the structure with enforced symmetry relaxation is used instead. After initializing the displacement structures, the force sets are calculated for each displacement. CPUs handle the remainder of the workflow. Force constants are constructed from the displacement force sets, and thermal conductivity is calculated using `phono3py`. Structures with imaginary phonon frequencies are discarded from the final analysis (conducted on a PC).

Simultaneous fine-tuning for multiple materials

To demonstrate the applicability and generalizability of fine-tuning fMLPs for thermomechanical properties, we simultaneously fine-tuned models for three distinct materials: wurtzite AlP, zincblende BN, and rocksalt LiBr, targeting both thermal conductivity and the Grüneisen parameter. For each material, three structural configurations were used: a rattled supercell (atomic displacements sampled with a standard deviation of 0.1 Å) and two unit cells uniformly scaled by $\pm 1\%$ in volume. This setup enhances the accuracy of stress predictions and yields relaxed structures that more closely align with DFT references.

Fig. 5 shows that the fMLP effectively learns across multiple materials in parallel, improving accuracy while reducing the per-observable training cost. Fine-tuning was performed using the same settings as for LiBr, on an NVIDIA Tesla A100 SXM4 80GB GPU.

Special checks on phonon mode contributions to thermal conductivity

Following the `phono3py` default, we include boundary scattering, $\Gamma_b(\mathbf{q})_s = \frac{|\mathbf{v}(\mathbf{q})_s|}{L}$ with $L = 1$ m, in the linewidth calculations in addition to the anharmonic and isotopic linewidths. For any phonon mode with non-zero group velocity, this boundary term makes the total linewidth finite and the mode-conductivity contribution well defined. The remaining pathological limit is a mode with zero group velocity and zero total linewidth, which can occur numerically, for example, for very flat bands when the anharmonic and isotopic linewidths also vanish. In this limit, the particle-like mode-conductivity contribution is analytically zero because $\mathcal{K}(\mathbf{q})_s \propto |\mathbf{v}(\mathbf{q})_s|^2/\Gamma_b(\mathbf{q})_s = |\mathbf{v}(\mathbf{q})_s| L \to 0$,

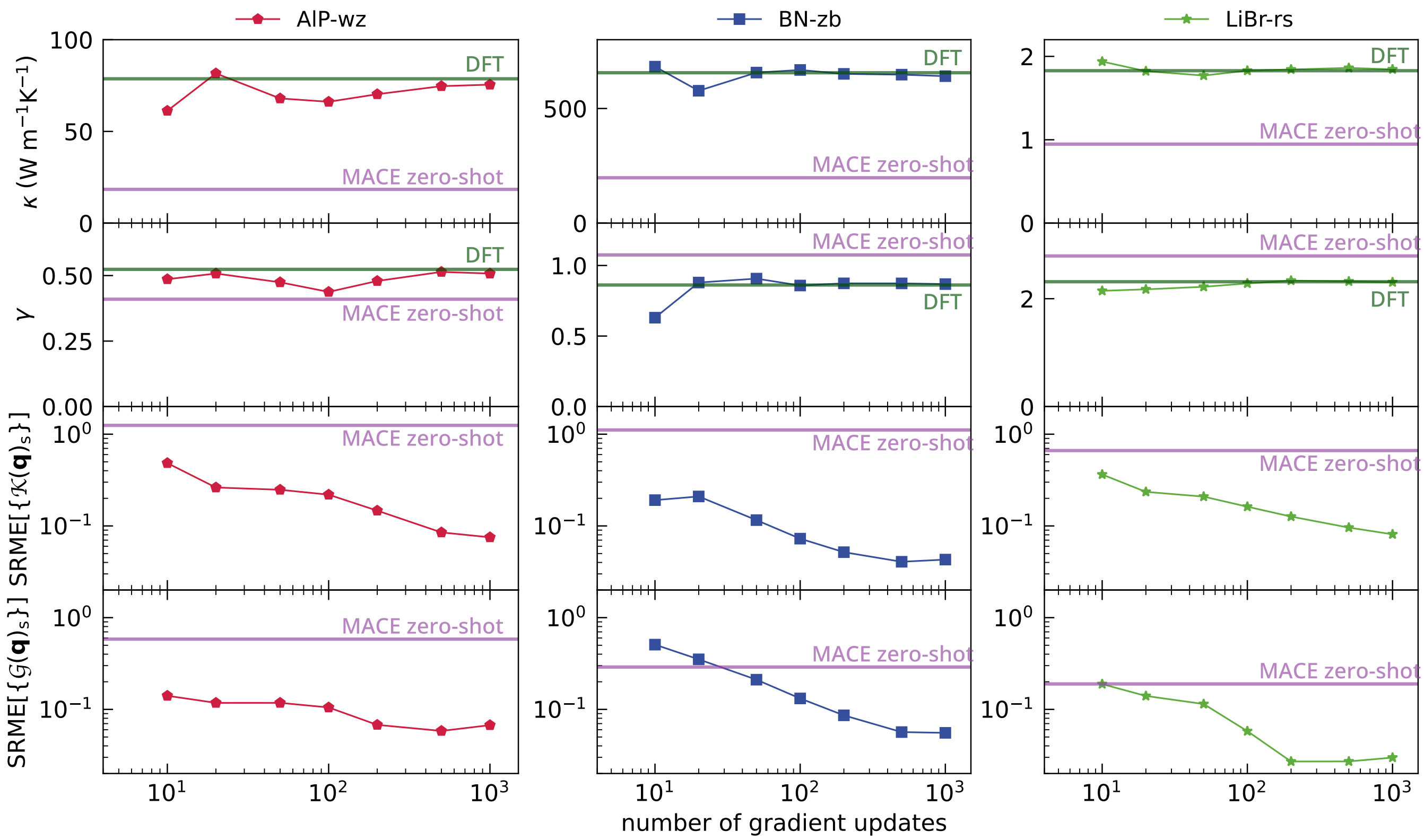

SUPPLEMENTARY FIGURE 5. **Improving foundation machine-learning potential accuracy via simultaneous fine-tuning of three materials.** Thermal conductivity, Grüneisen parameter, and the corresponding SRME values for wurtzite AlP, zincblende BN, and rocksalt LiBr after simultaneous fine-tuning on 3 frames (1 rattled supercell and 2 isotropically scaled unit cells), as a function of the number of gradient updates.

but direct numerical evaluation can yield an undefined value (`nan`). We therefore inserted a check that assigns zero mode conductivity to these cases. Very large but finite mode contributions are retained, since they reflect the harmonic and anharmonic properties predicted by the model and might be related to inaccuracies that should be detected.

Further Supplementary Figures

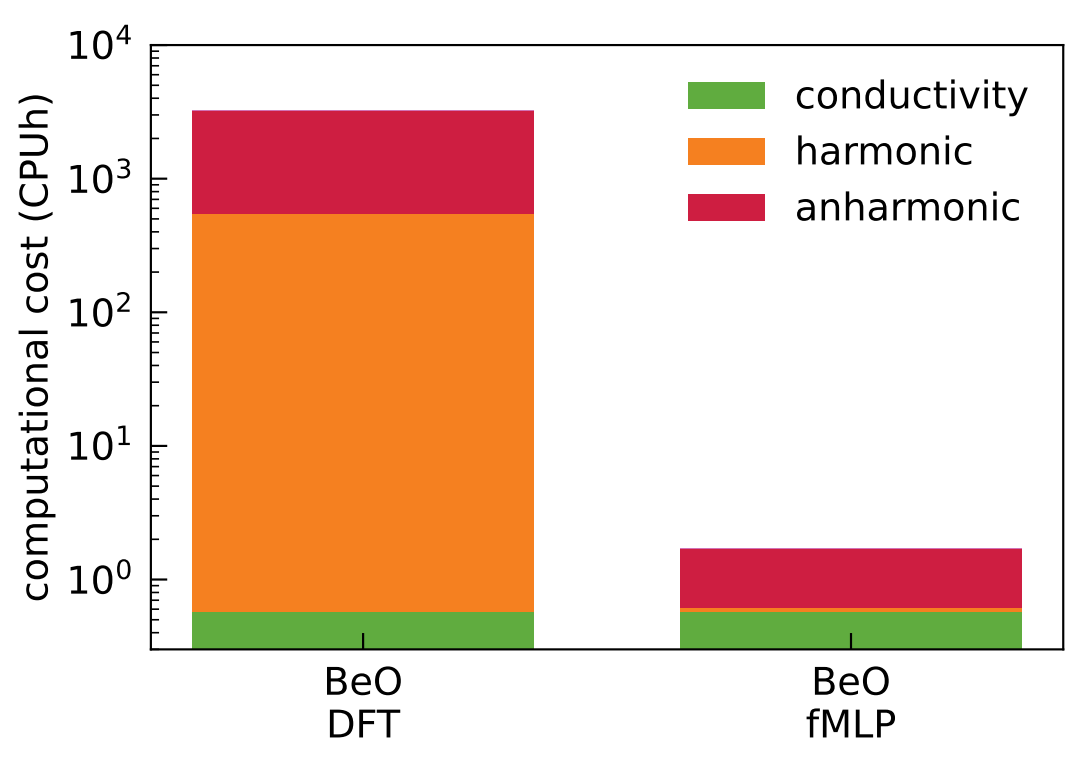

SUPPLEMENTARY FIGURE 6. **Computational cost: density functional theory (DFT) vs foundation machine-learning potential (fMLP)** MACE-MP-0-L2 for wurtzite BeO. Orange and red show the cost for computing harmonic and anharmonic force constants, respectively; green is the cost to obtain the conductivity from the force constants through the solution of the Wigner transport equation. The force constants were computed on CPUs for DFT and on GPUs for MACE (see text for details); these costs were compared using the conversion 1 GPUh = 55 CPUh[39].

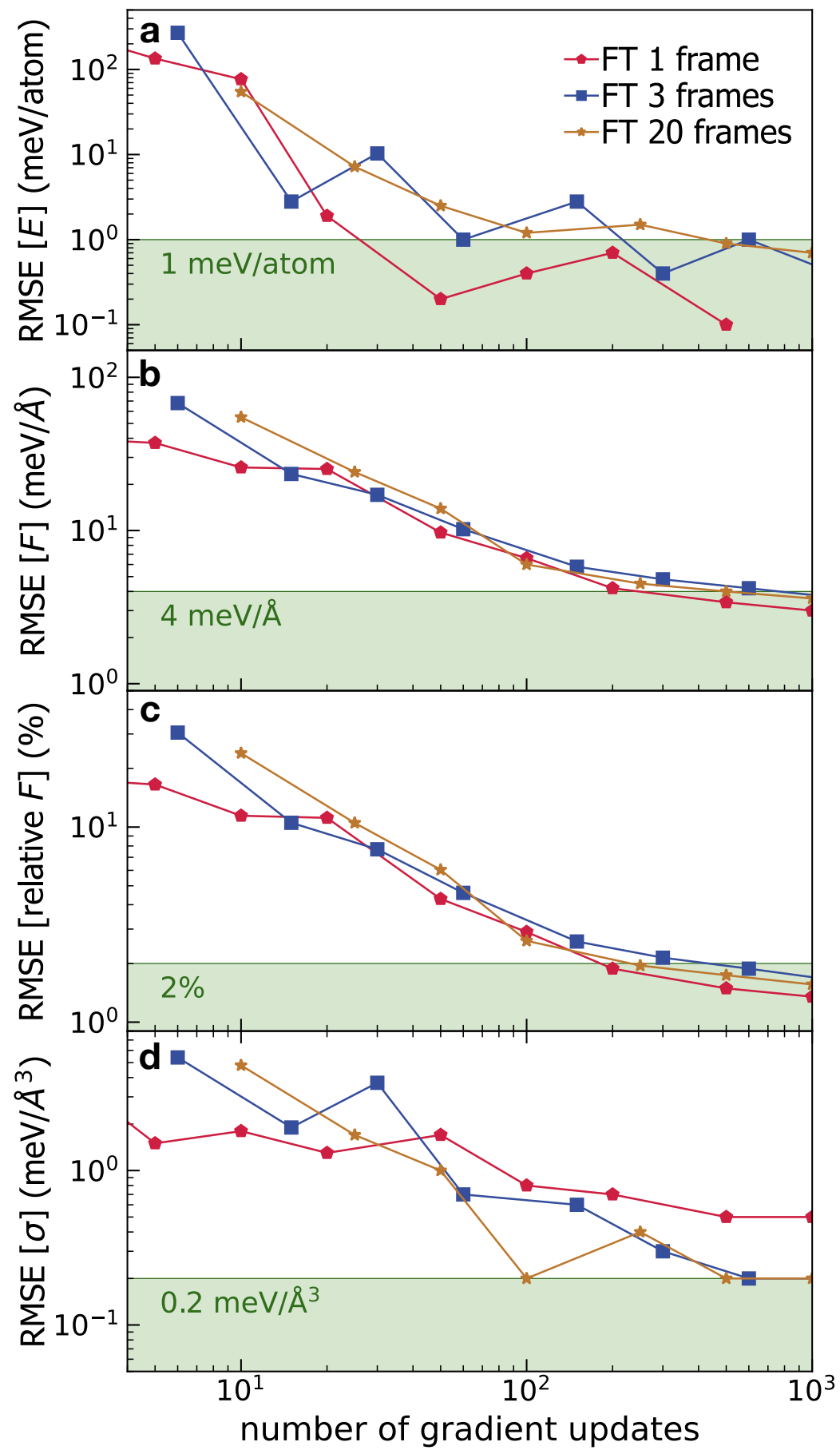

SUPPLEMENTARY FIGURE 7. **Improving foundation machine-learning potential accuracy in LiBr via fine-tuning.** Upon increasing the number of fine-tuning gradient updates, the root mean square training errors (RMSE) on energies (panel **a**), forces (**b** and **c**), and stresses (**d**) decrease. The green regions highlight the errors at the end of the 3-frame fine-tuning for MACE-MP-0, which was then used to produce Fig. 5**b–d** of the main text. These errors approximately correspond to a validation error at 300 K of 2% in SRE (Eq. (3) of the main text), and of 0.1 in SRME (Eq. (4) of the main text).

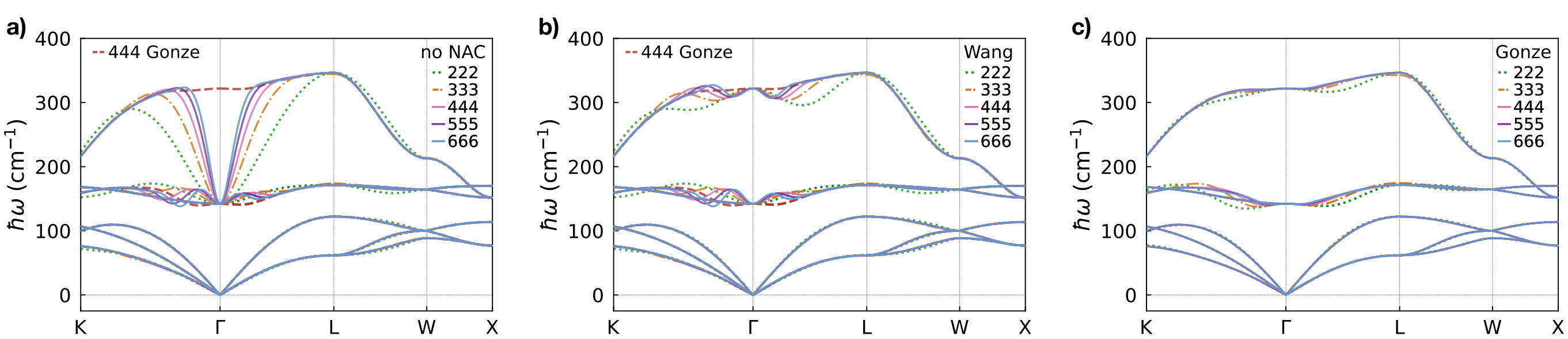

SUPPLEMENTARY FIGURE 8. **Numerical performance of different approaches to account for the effect of the non-analytical correction on phonon dispersion.** We show the phonon band structure of LiBr without the non-analytical correction (NAC) (a), including NAC using Wang's[38] approach (b) or Gonze's[40] method (c). These three cases are compared in simulations that use supercells of different sizes.

Supplementary References

[1] Simoncelli, M., Marzari, N. & Mauri, F. Unified theory of thermal transport in crystals and glasses. *Nature Physics* **15**, 809–813 (2019).
[2] Simoncelli, M., Marzari, N. & Cepellotti, A. Generalization of fourier's law into viscous heat equations. *Phys. Rev. X* **10**, 011019 (2020).
[3] Dragašević, J., Rajkov, B. & Simoncelli, M. Viscous Heat Backflow and Temperature Resonances in Extreme Thermal Conductors. *Physical Review Letters* **136**, 186302 (2026).
[4] Di Lucente, E., Simoncelli, M. & Marzari, N. Crossover from Boltzmann to Wigner thermal transport in thermoelectric skutterudites. *Physical Review Research* **5**, 033125 (2023).
[5] Jain, A. Multichannel thermal transport in crystalline tl_3vse_4. *Phys. Rev. B* **102**, 201201 (2020).
[6] Xia, Y. *et al.* High-throughput study of lattice thermal conductivity in binary rocksalt and zinc blende compounds including higher-order anharmonicity. *Phys. Rev. X* **10**, 041029 (2020).
[7] Shen, X. *et al.* Amorphous-Like Ultralow Thermal Transport in Crystalline Argyrodite Cu7PS6. *Advanced Science* **11**, 2400258 (2024).
[8] Pandey, T., Du, M.-H., Parker, D. S. & Lindsay, L. Origin of ultralow phonon transport and strong anharmonicity in lead-free halide perovskites. *Materials Today Physics* **28**, 100881 (2022).
[9] Fiorentino, A. & Baroni, S. From Green-Kubo to the full Boltzmann kinetic approach to heat transport in crystals and glasses. *Physical Review B* **107**, 054311 (2023).
[10] Zhou, S. *et al.* Phonon Thermal Transport in UO 2 via Self-Consistent Perturbation Theory. *Physical Review Letters* **132**, 106502 (2024).
[11] Zheng, J. *et al.* Unravelling ultralow thermal conductivity in perovskite Cs2AgBiBr6: dominant wave-like phonon tunnelling and strong anharmonicity. *npj Computational Materials* **10**, 30 (2024).
[12] Jia, F. *et al.* Cu3BiS3: Two-Dimensional Coordination Induces Out-of-Plane Phonon Scattering Enabling Ultralow Thermal Conductivity. *Angewandte Chemie* **135**, e202315642 (2023).
[13] Tadano, T. & Saidi, W. A. First-Principles Phonon Quasiparticle Theory Applied to a Strongly Anharmonic Halide Perovskite. *Physical Review Letters* **129**, 185901 (2022).
[14] Bernges, T. *et al.* Considering the Role of Ion Transport in Diffuson-Dominated Thermal Conductivity. *Advanced Energy Materials* **12**, 2200717 (2022).
[15] Yang, X., Tiwari, J. & Feng, T. Reduced anharmonic phonon scattering cross-section slows the decrease of thermal conductivity with temperature. *Materials Today Physics* **24**, 100689 (2022).
[16] Zeng, Z. *et al.* Pushing thermal conductivity to its lower limit in crystals with simple structures. *Nature Communications* **15**, 3007 (2024).
[17] Caldarelli, G., Simoncelli, M., Marzari, N., Mauri, F. & Benfatto, L. Many-body Green's function approach to lattice thermal transport. *Physical Review B* **106**, 024312 (2022).
[18] Xia, Y. *et al.* A unified understanding of minimum lattice thermal conductivity. *Proceedings of the National Academy of Sciences* **120**, e2302541120 (2023).
[19] Luo, Y., Yang, X., Feng, T., Wang, J. & Ruan, X. Vibrational hierarchy leads to dual-phonon transport in low thermal conductivity crystals. *Nature Communications* **11**, 2554 (2020).
[20] Pazhedath, A., Bastonero, L., Marzari, N. & Simoncelli, M. First-principles characterization of thermal conductivity in $lapo_4$-based alloys. *Phys. Rev. Appl.* **22**, 024064 (2024).
[21] Harper, A. F., Iwanowski, K., Witt, W. C., Payne, M. C. & Simoncelli, M. Vibrational and thermal properties of amorphous alumina from first principles. *Physical Review Materials* **8**, 043601 (2024).
[22] Liu, Y. *et al.* Unraveling thermal transport correlated with atomistic structures in amorphous gallium oxide via machine learning combined with experiments. *Advanced Materials* **35**, 2210873 (2023).
[23] Ndour, M., Jund, P. & Chaput, L. Practical approach to thermal conductivity calculations of small SiO2 samples. *Journal of Non-Crystalline Solids* **621**, 122618 (2023).
[24] Lundgren, N. W., Barbalinardo, G. & Donadio, D. Mode localization and suppressed heat transport in amorphous alloys. *Physical Review B* **103**, 024204 (2021).
[25] Kielar, S. *et al.* Anomalous lattice thermal conductivity increase with temperature in cubic GeTe correlated with strengthening of second-nearest neighbor bonds. *Nature Communications* **15**, 6981 (2024).
[26] Simoncelli, M. *et al.* Temperature-invariant crystal–glass heat conduction: From meteorites to refractories. *Proceedings of the National Academy of Sciences* **122**, e2422763122 (2025).
[27] Simoncelli, M., Marzari, N. & Mauri, F. Wigner Formulation of Thermal Transport in Solids. *Physical Review X* **12**, 041011 (2022).
[28] Togo, A., Chaput, L. & Tanaka, I. Distributions of phonon lifetimes in brillouin zones. *Phys. Rev. B* **91**, 094306 (2015).
[29] Seko, A. *et al.* Prediction of Low-Thermal-Conductivity Compounds with First-Principles Anharmonic Lattice-Dynamics

Calculations and Bayesian Optimization. *Physical Review Letters* **115**, 205901 (2015).
[30] Ziman, J. M. *Electrons and Phonons: The Theory of Transport Phenomena in Solids* (Oxford University Press, New York, 1960).
[31] Batatia, I. *et al.* A foundation model for atomistic materials chemistry. *The Journal of Chemical Physics* **163**, 184110 (2025).
[32] Deng, B. *et al.* Systematic softening in universal machine learning interatomic potentials. *npj Computational Materials* **11**, 9 (2025).
[33] Larsen, A. H. *et al.* The atomic simulation environment—a python library for working with atoms. *Journal of Physics: Condensed Matter* **29**, 273002 (2017).
[34] Togo, A., Chaput, L., Tadano, T. & Tanaka, I. Implementation strategies in phonopy and phono3py. *Journal of Physics: Condensed Matter* **35**, 353001 (2023).
[35] Togo, A. & Tanaka, I. `Spglib`: a software library for crystal symmetry search. Preprint at https://arxiv.org/abs/1808.01590 (2018).
[36] Gonze, X. & Lee, C. Dynamical matrices, born effective charges, dielectric permittivity tensors, and interatomic force constants from density-functional perturbation theory. *Physical Review B* **55**, 10355–10368 (1997).
[37] Togo, A. First-principles Phonon Calculations with Phonopy and Phono3py. *Journal of the Physical Society of Japan* **92**, 012001 (2023).
[38] Wang, Y. *et al.* A mixed-space approach to first-principles calculations of phonon frequencies for polar materials. *Journal of Physics: Condensed Matter* **22**, 202201 (2010).
[39] The Cambridge Service for Data Driven Discovery (CSD3), cost of CPU and GPU hours (2024-09-02). https://docs.hpc.cam.ac.uk/hpc/user-guide/upgrade2021.html.
[40] Gonze, X. & Lee, C. Dynamical matrices, Born effective charges, dielectric permittivity tensors, and interatomic force constants from density-functional perturbation theory. *Physical Review B* **55**, 10355–10368 (1997).
