# Supplementary material for: Thermal conductivity predictions with foundation atomistic models
Source: Nat Commun. 2026 Sep 5;17:10566. doi: 10.1038/s41467-026-76391-w (PMC13642343; doi:10.1038/s41467-026-76391-w)
Supplement: Supplementary file 2 [file 41467_2026_76391_MOESM2_ESM.pdf]

nature portfolio

# Peer Review File

# Thermal Conductivity Predictions with Foundation Atomistic Models

Corresponding Author: Dr Michele Simoncelli

This manuscript has been previously reviewed at another journal. This document only contains information relating to versions considered at Nature Communications.

**This file contains all reviewer reports in order by version, followed by all author rebuttals in order by version.**

Version 0:

Reviewer comments:

Reviewer #1

(Remarks to the Author)
This is a good piece of work. I would love to see it be published at Nat Comm. It seems the authors have already gone through at least one round of review, which addressed some of the questions I have. That being said, I still have a few outstanding questions before giving it a green light:
1. How robust is the method? We've tested the code on many structures. Some of them give rise to singularities, say very high tthermal conductivity contribution at specific frequencies. I think this should be something to address. At least, there should be some ad hoc checking protocol to manage the breakdown of the prediction.
2. The code leverages finite difference for force constants. With different step size on such finite difference, the final conductivity value may change a lot. And different models perform quite differently. Some give very different kappa while some give consistent results. How is the default step size chosen? Will the performance order change if different step size is chosen? Will it give different results on what has been analyzed?
3. I suggest the author hold out a small dataset to benchmark the models independently. So that these models are less likely to overfit on the benchmark.

Again, I'd love to see this paper published. Nice work.

(Remarks on code availability)

Version 1:

Reviewer comments:

Reviewer #1

(Remarks to the Author)
Looks good to me. I'd like to see it publish.

(Remarks on code availability)

**Open Access** This Peer Review File is licensed under a Creative Commons Attribution 4.0 International License, which permits use, sharing, adaptation, distribution and reproduction in any medium or format, as long as you give appropriate credit to the original author(s) and the source, provide a link to the Creative Commons license, and indicate if changes were made.
In cases where reviewers are anonymous, credit should be given to 'Anonymous Referee' and the source.
The images or other third party material in this Peer Review File are included in the article's Creative Commons license, unless indicated otherwise in a credit line to the material. If material is not included in the article's Creative Commons license and your intended use is not permitted by statutory regulation or exceeds the permitted use, you will need to obtain permission directly from the copyright holder.
To view a copy of this license, visit https://creativecommons.org/licenses/by/4.0/

# Response to Reviewers for "Thermal Conductivity Predictions with Foundation Atomistic Models"


Balázs Póta,[1] Paramvir Ahlawat,[1] Gábor Csányi,[2] and Michele Simoncelli[1,3]

[1] *Theory of Condensed Matter Group of the Cavendish Laboratory, University of Cambridge, Cambridge, UK*
[2] *Engineering Laboratory, University of Cambridge, Cambridge, UK*
[3] *Department of Applied Physics and Applied Mathematics, Columbia University, New York (USA)*


## NOTE FOR THE EDITOR

We thank the Editor for considering our manuscript in Nature Communications. We thank the Reviewer for the constructive, insightful and valuable comments along with their positive evaluation. In this revised version, we have addressed these comments with the following points:

1. We have added a paragraph explaining how to interpret the values of the mode-resolved thermal conductivity, and we updated the `k_SRME` package to handle cases in which very large values could emerge due to inaccurate potentials or exotic physics (zero-group-velocity or zero-linewidth limit).
2. We have included a detailed analysis of the effect of the finite-difference displacement amplitude on the force constants and the derived thermomechanical properties. We discuss how to set default values for the displacement amplitude, and possible material-dependent uncertainties on the physical properties that originate from the finite precision with which numerical derivatives are evaluated.
3. To discuss and estimate the transferability of our findings, we have also computed the thermal conductivity (`k_SRME`) and Grüneisen parameter ($\gamma$`_SRME`) of two additional materials. We have observed that the performance correlates strongly with the $\kappa_{SRME}$ and $\gamma_{SRME}$ computed on the 103 materials from the phononDB-PBE reference dataset.

We report below a detailed point-by-point answer to the Reviewers' comments. As a convention, text in *italic black* is original text written by the Reviewers. The text quoted from the article is:
- blue if unchanged compared to the original version,
- green if added in the revised version,
- ~~strikethrough red if deleted (no longer in the revised article)~~.

We also attach a version of the document with modifications with respect to the previous version highlighted.

## REVIEWER

**Reviewer:** *This is a good piece of work. I would love to see it be published at Nat Comm. It seems the authors have already gone through at least one round of review, which addressed some of the questions I have.*

**Response:** We thank the Reviewer for the positive evaluation of our work, and for recognizing our earlier efforts on improving the quality of the manuscript.

**Reviewer:** *That being said, I still have a few outstanding questions before giving it a green light: 1. How robust is the method? We've tested the code on many structures. Some of them give rise to singularities, say very high thermal conductivity contribution at specific frequencies. I think this should be something to address. At least, there should be some ad hoc checking protocol to manage the breakdown of the prediction.*

**Response:** This is an important and useful comment. While the problem discussed by the Reviewer has not emerged in the materials we studied so far, we agree that it is possible in principle. Without knowing the specific material and foundation model that produce these very high thermal-conductivity contributions, we discuss the potential origins of this problem and the related checking protocols.
Within the Wigner Transport Equation (WTE), exceedingly high mode thermal-conductivity contributions can be caused by extremely large group velocities or vanishing linewidths. In fact, for crystalline materials, the Peierls-Boltzmann conductivity is recovered from the diagonal terms of the WTE. In the Peierls-Boltzmann conductivity,

the linewidths enter the denominator and can yield diverging thermal-conductivity contributions, while the group velocities enter squared in the numerator.

In particular, unphysically steep phonon bands can create very large group velocities, which could cause substantial contributions to the thermal conductivity. In polar materials, neglecting the non-analytical term correction (NAC)[1] can overestimate group velocities. However, for all the 103 materials studied in this work, we have compared the thermal-conductivity predictions considering or neglecting the NAC term, and in Fig. 14 we verified that variations are always $\lesssim$10% (less than 5% for 80% of the materials studied). In the present manuscript, we discuss how the NAC contributions can be accounted for in a hybrid DFT-fMLP approach, and more generally, in the future, they could be computed using electrostatic fMLP models such as MACE-Field-MP-0[2]. However, these recent models are still under active development and have not yet been systematically benchmarked for these applications.

Focusing on the other possible source of the problem, i.e., a very small linewidth, we note that the effective linewidth of a real finite crystal is never exactly zero once all relevant scattering mechanisms are included, as a real quantum system is not perfectly harmonic and must have a finite lifetime. The anharmonic linewidths depend on third- and higher-order derivatives of the potential energy surface (PES), and therefore can be significantly influenced by the accuracy of fMLPs; in some cases, they might give rise to vanishing anharmonic linewidths for particular phonon modes. Given that fMLPs can have difficulties in describing the PES, we think that fMLP inaccuracies could be the most likely source of such very high conductivity contributions. However, from a theoretical point of view, very high conductivity contributions could originate from missing scattering processes such as four-phonon scattering[3], or from neglecting collisional broadening. The latter mechanism has recently been shown to extend the BTE by leading to coupling between phonon modes that do not necessarily conserve energy in Refs.[4,5], and hence to a larger number of scattering channels that are less likely to produce vanishing numerical linewidths. These developments have not yet been implemented in `phono3py`, and while we acknowledge that they might be relevant in some exotic cases, we discuss numerical and physical mechanisms that allow us to neglect these effects for benchmarking purposes.

First, real materials have a finite size, which causes a finite thermal conductivity even in the absence of anharmonicity[6]. To see this, we recall that the established Casimir model[6] for phonon-boundary scattering attributes a finite lifetime to all phonons with non-zero group velocity, as described by the following equation[7],

$$\tau_b(\boldsymbol{q})_s = [\Gamma_b(\boldsymbol{q})_s]^{-1} = \frac{L}{|\boldsymbol{v}(\boldsymbol{q})_s|}\,, \tag{1}$$

where $L$ is the boundary mean free path, and $|\boldsymbol{v}(\boldsymbol{q})_s|$ is the Cartesian norm of the group velocity at wavevector $\boldsymbol{q}$ and band $s$. In the `k_SRME` package, we use the `phono3py` default $L = 1$ m for the boundary mean free path. In the special case of a flat band with zero group velocity, the single-phonon contribution to the conductivity is trivially zero, because $\mathcal{K}(\boldsymbol{q})_s \propto ||\boldsymbol{v}(\boldsymbol{q})_s||^2\tau_b(\boldsymbol{q})_s \propto ||\boldsymbol{v}(\boldsymbol{q})_s||L = 0$. For the coherence conductivity, it is straightforward to show that it vanishes in crystalline materials when the linewidth approaches zero[8], and therefore it cannot yield diverging conductivity even in the limit of anharmonic linewidths.

We note that the brute-force numerical evaluation of the exotic case of zero group velocity and zero linewidth would yield the ratio between two zeros. Because we have shown that, from an analytical viewpoint, the particle-like mode-resolved conductivity in such a case is zero, we have modified the `k_SRME` package to ensure that zero is obtained if this special case emerges.

In cases where the mode contribution to the thermal conductivity is extremely large, but finite, the most probable explanation is that the force constants obtained from fMLPs yield extremely low anharmonic linewidths. For benchmarking purposes, we believe that reproducing the DFT force constants and linewidths is important, and very large but non-diverging contributions should not be filtered from the $\kappa_{SRME}$ metric, as they would be indicative of inaccuracies of the fMLP.

Finally, considering that `phono3py` (on which the `k_SRME` package is based) has been continuously debugged for the past 10 years, widely used by academia and industry, and tested against multiple other codes[9], we consider it unlikely that these very high contributions originate from bugs in the underlying transport solver.

**Action:** We have updated the `k_SRME` package to detect anomalous values arising from the zero-group-velocity/zero-linewidth limit and to assign the analytically correct value, zero, to those modes. Very large but finite mode contributions are left unchanged, because they signal inaccurate harmonic or anharmonic properties of the fMLP and should be reflected in the benchmark. This does not change any already computed results for the models studied here. Please find the details in the following pull request: https://github.com/MPA2suite/k_SRME.

We have also added an explanation in the Methods:

**Special checks on phonon mode contributions to thermal conductivity**

Following the `phono3py` default, we include boundary scattering, $\Gamma_b(\boldsymbol{q})_s = \frac{|\boldsymbol{v}(\boldsymbol{q})_s|}{L}$ with $L = 1$ m, in the linewidth

calculations in addition to the anharmonic and isotopic linewidths. For any phonon mode with non-zero group velocity, this boundary term makes the total linewidth finite and the mode-conductivity contribution well defined. The remaining pathological limit is a mode with zero group velocity and zero total linewidth, which can occur numerically, for example, for very flat bands when the anharmonic and isotopic linewidths also vanish. In this limit, the particle-like mode-conductivity contribution is analytically zero because $\mathcal{K}(\boldsymbol{q})_s \propto |\boldsymbol{v}(\boldsymbol{q})_s|^2/\Gamma_b(\boldsymbol{q})_s = |\boldsymbol{v}(\boldsymbol{q})_s|L \to 0$, but direct numerical evaluation can yield an undefined value (`nan`). We therefore inserted a check that assigns zero mode conductivity to these cases. Very large but finite mode contributions are retained, since they reflect the harmonic and anharmonic properties predicted by the model and might be related to inaccuracies that should be detected.

**Reviewer:** *2. The code leverages finite difference for force constants. With different step size on such finite difference, the final conductivity value may change a lot. And different models perform quite differently. Some give very different kappa while some give consistent results. How is the default step size chosen? Will the performance order change if different step size is chosen? Will it give different results on what has been analyzed?*

**Response:** The accuracy of the finite-displacement (FD) method used to evaluate derivatives of the potential-energy surface is indeed a subtle aspect that requires careful discussion. Here, we discuss it both from the perspective of benchmarking foundation machine learning potentials (fMLPs) and from a more general property-prediction point of view.

There are two competing limitations to the accuracy of fMLP predictions of thermal conductivity when different FD displacement amplitudes are used. We start by considering the "ground truth" potential-energy surface (PES) of a specific atomic structure, in our case predicted from DFT. The derivatives of this PES computed by the FD method can be affected by an error that depends on the displacement amplitude, since the derivatives are defined in the limit of infinitesimal displacement but a finite displacement has to be used for numerical reasons. More specifically, in DFT the finite displacements have to be chosen within a physically and numerically sensible range, ensuring that force perturbations are numerically large enough to be above the DFT noise floor and physically small enough to be treated as perturbations. In standard Born-Oppenheimer DFT calculations, varying the finite displacement within the range 0.005–0.05 Å produces small differences in vibrational and thermal properties, although the sensitivity can be material dependent, as discussed below.

Since a good MLP is expected to reproduce the DFT PES, for a fixed finite displacement a good MLP is expected to reproduce the reference DFT value computed with the same displacement. Importantly, the weak dependence of DFT vibrational and thermal properties on the finite displacement is material dependent; therefore, an fMLP that accurately reproduces DFT data for vibrational and thermal properties of many materials should display a weak or negligible dependence on the finite displacement, as DFT does. Whenever an MLP predicts vibrational properties with a dependence on the displacement larger than that observed in reference DFT data, it means that the MLP fails to reproduce the smoothness of the DFT PES. In particular, the PES encoded by fMLPs can be significantly less smooth than the DFT one[10]; therefore, the displacement amplitude can significantly influence the relative error in the FD force constants, propagating to observable quantities such as thermal conductivity (see also Refs.[11,12]). Comparing small-displacement DFT with large-displacement fMLP can result in cancellation of errors; hence, it is important to choose the FD displacement amplitude consistently when benchmarking DFT and fMLPs. Additionally, using too large a displacement can alter the apparent dynamical stability in certain materials.

To illustrate the concepts above with an example, we show in Fig. 1 how the DFT PES changes near equilibrium in zincblende BN (a high thermal conductor with weak anharmonicity) and rocksalt PbTe (a low thermal conductor with strong anharmonicity, including significant fourth-order anharmonic effects[13]). We plot the $x$-directional component of the forces on different atoms in $2 \times 2 \times 2$ supercells against the displacement amplitude of the atom with index 0. The open markers correspond to positive displacements, the filled markers correspond to negative displacements, and the absolute value of the force components is plotted. The different lines and markers correspond to forces exerted on different atoms. This is indicated in the legend by providing the index, the chemical species, and the minimum-image-convention distance from the displaced atom.

The linear behavior in the DFT force in Fig. 1 corresponds to the harmonic (second-order) dependence of the energy with respect to the displacement. In general, two displaced atoms are required to calculate higher-order force constants. However, even from displacements of a single atom, we can compute force-constant elements that have at most one index associated with another atom (e.g., $iij$ or $iiij$ atom indices). Therefore, the upward or downward deviations between the positive and negative displacements at larger amplitudes (in Fig. 1) represent the third-order anharmonicity, while deviations in a common direction show the fourth-order anharmonicity. We note that the harmonic (dashed) and third-order (dotted) fits are obtained at the default 0.03Å displacement amplitude.

As expected, for both BN (Fig. 1**a**) and PbTe (Fig. 1**b**), the forces are dominated by the harmonic, linear term, indicating that a Taylor expansion around a local minimum of the PES is valid in this range. At low displacements, some forces can be lower than the relaxation force threshold, which we represent by $10^{-6}$ Ha/Bohr = $5.14 \times 10^{-5}$ eV/Å, similar to the threshold applied in the Materials Project phonon dataset[14]. This can be significant for very weak force constant elements even for the harmonic term, and much more pronounced for the third-order term. In this region of the displacement amplitude, we expect less reliable results and possible influence from noise—in particular, even if the current plots are smooth for small displacements, this might be due to their crystal symmetries, and more generally in complex glassy materials it is not reliable to compute FD quantities from force differences that are comparable to the force-convergence threshold.

To further assess the effect of the FD displacement amplitude on the derived thermomechanical quantities from DFT calculations, we have computed the thermal conductivity and the Grüneisen parameter for zincblende BN and rocksalt PbTe with 0.1Å, 0.05Å, 0.03Å, 0.02Å, and 0.01Å FD displacement amplitudes. Since the symmetric relative mean error (SRME) provides a symmetric comparison between two sets of mode-resolved quantities and is invariant to exchanging the reference and estimate, we use SRME between these DFT calculations. In contrast to comparing fMLPs, here the reference and the estimate are both DFT-computed quantities. Therefore,

$$\begin{aligned}
\mathrm{SRME}\big[\{\mathcal{K}(\boldsymbol{q})_s\}_{\mathrm{DFT,DFT}}\big] &= \frac{2}{N_c\mathcal{V}}\frac{\sum_{\boldsymbol{q}s}|\mathcal{K}_{\mathrm{DFT1}}(\boldsymbol{q})_s - \mathcal{K}_{\mathrm{DFT2}}(\boldsymbol{q})_s|}{\kappa_{\mathrm{DFT1}}+\kappa_{\mathrm{DFT2}}},\\
\mathrm{SRME}\big[\{\mathcal{G}(\boldsymbol{q})_s\}_{\mathrm{DFT,DFT}}\big] &= \frac{2}{N_c\mathcal{V}}\frac{\sum_{\boldsymbol{q}s}|\mathcal{G}_{\mathrm{DFT1}}(\boldsymbol{q})_s - \mathcal{G}_{\mathrm{DFT2}}(\boldsymbol{q})_s|}{\sum_{\boldsymbol{q}s}|\mathcal{G}_{\mathrm{DFT1}}(\boldsymbol{q})_s| + \sum_{\boldsymbol{q}s}|\mathcal{G}_{\mathrm{DFT2}}(\boldsymbol{q})_s|},
\end{aligned} \tag{2}$$

where $\kappa$ is the mean thermal conductivity, $\mathcal{K}(\boldsymbol{q})_s$ is the thermal conductivity contribution, $\mathcal{G}(\boldsymbol{q})_s$ is the Grüneisen parameter contribution of a phonon mode at wavevector $\boldsymbol{q}$ and band $s$, and DFT1 and DFT2 represent the two sets of quantities computed with different settings (displacement amplitudes). This way, we compute the SRMEs between mode-resolved thermal conductivities and Grüneisen parameters obtained with different FD displacement amplitudes. The resulting SRME matrix is symmetric with vanishing diagonals across the five amplitudes for both quantities, as shown in Fig. 2**a,b** for zincblende BN and rocksalt PbTe, respectively. The lower triangular matrix represents the SRMEs of thermal conductivity, while the upper matrix represents the Grüneisen parameter for each material. We use shared colormaps for the materials, starting from blue and green for thermal conductivity and Grüneisen parameter progressing toward red. The upper limits of the colormaps were chosen to be 0.2 and 0.1 for conductivity and Grüneisen parameter, respectively, as these represent significant differences in the SRME values, slightly larger than the results from recent fMLPs. Since SRME in mode-resolved conductivity appears to be about twice as high as SRME in mode-resolved Grüneisen parameter, this is reflected in the colormap limits. To guide the eye, a double border is drawn around matrix elements of the converged values, from 0.01Å to 0.03Å displacement amplitudes.

In Fig. 2**c**, we plot the relative difference in the macroscopic thermal conductivity and Grüneisen parameter at 300 K against FD displacement amplitude relative to the default 0.03Å.

For BN, the changes in both the macroscopic and the microscopic, mode-resolved quantities are minor, which is explained by the low anharmonicity and hence large thermal conductivity in this material. Conversely, in PbTe, both the macro- and microscopic quantities exhibit large deviations for 0.1Å and 0.05Å displacement amplitudes, becoming less significant, but still present between 0.01Å and 0.03Å. This confirms that the finite-difference displacement amplitude can have a significant effect on the force constants and the derived thermomechanical quantities.

We note that the default `phono3py` displacement amplitude of 0.03Å appears to be sufficient. Importantly, this is also the amplitude employed by the DFT datasets[15,16] referenced in this study, and by the Grüneisen-parameter dataset computed here to remain consistent. However, other codes might use different default settings. For second-order force constants, `phonopy` uses default values of 0.01Å and 0.02 bohr (0.0106Å), depending on the default units of the software; VASP uses 0.015Å; CP2K uses 0.01 bohr (0.0053Å); and FHI-aims uses 0.0025Å as the default FD displacement amplitude. For third-order force constants, the `thirdorder.py` code of the `ShengBTE` package uses 0.01Å[17]. We also note that larger displacement amplitudes are used in projector-based estimation with random displacements[18]. In general, the larger the displacement amplitude, the more the previously mentioned fourth-order effects influence the FD results. However, as we showed above, the importance of this amplitude is material dependent.

These displacement amplitudes are chosen for DFT calculations and are set to balance the noise in the DFT calculation, errors due to higher-order anharmonicity, and residual forces after relaxation. Therefore, when assessing the utility of fMLPs in computing thermal conductivity, we need to consider the noise, the artifacts, and the discontinuities in the PES described by the fMLPs, not the DFT. Similarly to DFT, this is software- and architecture-dependent but can be

more significant, because in certain models and materials, the noisy regime may overlap with the displacement range where higher-order anharmonicity becomes significant. Using larger displacement amplitudes may seem to overcome the noise, but as shown above, this can lead to modified force constants, masked discontinuities in the PES, and significant changes in the derived quantities.

However, DFT reference data are only available with 0.03Å displacement amplitude across the 103 binary compounds in the phononDB-PBE dataset, which limits the freedom of choosing displacement amplitudes for benchmarking purposes. Fig. 2 shows that larger displacement amplitudes can in fact have a significant effect on the SRME of both studied thermomechanical quantities, comparable to or larger than the results of the state-of-the-art models[10,12]. This provides a practical estimate of the SRME scale introduced by changing displacement amplitudes, since error cancellation is less probable given the large number of phonon mode contributions evaluated in the SRME, unlike when considering a single macroscopic quantity. Although this error cancellation is not expected, a target model evaluated against the current 0.03Å DFT reference could in principle have a lower SRME at a different displacement amplitude than the DFT-DFT SRME computed above for that same displacement-amplitude change. Therefore, comparative benchmarking should only use the original 0.03Å displacement amplitude.

However, we do not mean that benchmarking with a displacement amplitude different from 0.03Å is not useful. For model developers who want to understand their model architecture or differences in hyperparameter settings, it can be highly valuable to compare the $\kappa_{SRME}$ values computed with different FD displacement amplitudes against the same 0.03Å DFT reference.

From a utility point of view, this noise limitation could be overcome by fitting the force constants on structures with random displacements using the fMLP as a surrogate model[19]. Effectively, this distills the fMLP into a highly constrained model for small displacements around the equilibrium. However, achieving better results with compressive sensing[19] can mask these artifacts and therefore does not necessarily indicate a better description of the PES, as these artifacts can influence other computed properties[10,20] or energy conservation[10,21]. Therefore, we believe that thermomechanical quantities derived from finite-difference force constants are effective, transferable, and physically motivated benchmarks that test both smoothness and property-prediction utility at the same time.

**Action:** We have included this analysis in the Methods section:

**Effect of finite-difference displacement amplitude on conductivity and Grüneisen parameter.**
We use the finite-difference (FD) method to compute force constants, and in this section we discuss how the displacement amplitude influences conductivity and Grüneisen parameter. In DFT, the FD displacements have to be chosen within a physically and numerically sensible range, ensuring that force perturbations are numerically large enough to be above the DFT noise floor and physically small enough to be treated as perturbations. To balance these effects, a displacement amplitude of 0.03Å is employed in standard Born-Oppenheimer DFT calculations[15,16], and more generally a finite displacement within the range 0.005–0.05 Å is chosen as a default in other works[17]. In most DFT simulations, varying the FD amplitude within this range produces minor differences in the macroscopic thermomechanical properties, since the DFT PES is generally smooth (more on this later). An accurate fMLP is expected to reproduce the DFT PES, and hence to display the same weak or negligible dependence between FD amplitude and thermomechanical properties. However, due to suboptimal architectural choices or limited training data, fMLPs can encode a PES significantly less smooth than the DFT one[10,12]; in these cases, DFT and fMLPs can display very different relations between FD amplitude and derived thermomechanical quantities. To analyze these effects, we first illustrate the behavior of the DFT PES near the equilibrium of two example materials, zincblende BN and rocksalt PbTe, in Fig. **1a,b**. The atom with index 0 is displaced by different amplitudes along the $x$-direction, and the corresponding forces are computed with DFT in a $2 \times 2 \times 2$ supercell. We obtain the dependence of the atomic forces on the displacement amplitude, which is plotted for some selected atoms. The details of these selected atoms are provided in the legend, including their index, chemical species, and minimum-image-convention distance from the displaced atom. These forces are used in the FD method to compute the force-constant elements representing the derivatives of the PES with respect to these displacements. In particular, the harmonic, second-order force constants are obtained as the first numerical derivative of the force with respect to this displacement, while the third-order force constants are calculated as second derivatives. We plot the linear fit to the force (dashed), and the quadratic fit (dotted), which are obtained from the harmonic and the third-order force constant elements, respectively, computed with a displacement amplitude of 0.03Å. This correctly explains the increasing separation between the positive (open markers) and negative (filled markers) displacement directions of the red markers on the right-hand side of Fig. **1b**, corresponding to large third-order force constants on the logarithmic scale. However, we observe an offset in the same direction in the case of the orange markers, which is caused by the fourth-order

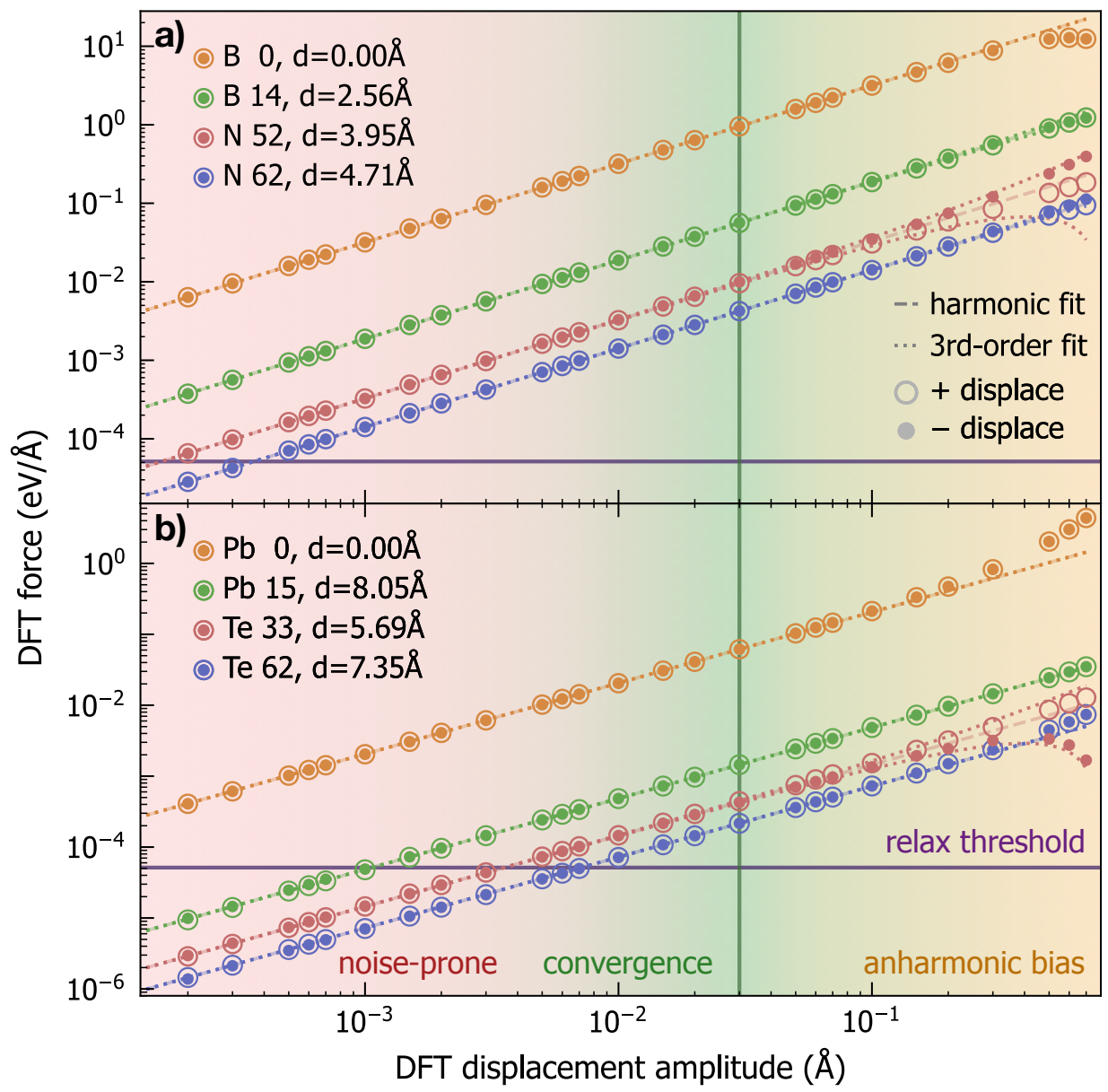


FIG. 1. **DFT forces on different atoms as a function of the displacement amplitude of the index 0 atom in a) zincblende BN and b) rocksalt PbTe** in a $2 \times 2 \times 2$ supercell. The open (filled) markers correspond to positive (negative) displacements, and the absolute value of the $x$-directional component of the forces is plotted. The different colored lines and markers correspond to forces exerted on different atoms (the legend provides the index, the chemical species, and the minimum-image-convention distance from the displaced atom). The linear behavior in the force (dashed line) corresponds to the harmonic dependence of the energy on the displacement. The vertical green line represents the default FD amplitude 0.03Å, while the horizontal purple line indicates the possible residual force magnitude corresponding to a relaxation threshold. The increasing separation of the filled and open markers represents third-order anharmonicity (dotted line), while deviations in a common direction show fourth-order anharmonicity. The shaded regions indicate the noise-prone regime (red), the converged FD regime (green), and the regime dominated by higher-order anharmonic bias (orange).

contribution becoming significant at large displacement amplitudes. This leads to errors in the finite-difference force constants computed at these large amplitudes. We illustrate this anharmonicity-biased region with the orange shaded area. In the opposite low-displacement regime (red shaded area), some forces can be lower than the relaxation force threshold, which we represent by $10^{-6}$ Ha/Bohr $= 5.14 \times 10^{-5}$ eV/Å, similar to the threshold applied in the Materials Project phonon dataset[14]. This can be significant for very weak force constant elements even for the harmonic term, and much more pronounced for the third-order term. In this region of the displacement amplitude, we expect less reliable results and possible influence from noise—in particular, even if the current plots are smooth for small displacements, this might be due to their crystal symmetries, and more generally in complex glassy materials it is not reliable to compute FD quantities from force differences that are comparable to the DFT force-convergence threshold. Between these two regimes, converged force constants can be calculated, as shown by the green shaded area.

To analyze the effect of the displacement amplitude not only on the force constants, but also on the derived thermomechanical quantities, we have computed the thermal conductivity and the Grüneisen parameter with DFT at 0.1Å, 0.05Å, 0.03Å, 0.02Å, and 0.01Å displacement amplitudes for zincblende BN and rocksalt PbTe. To have a comprehensive picture and resolve possible error cancellation, we compare both the macroscopic quantities and the microscopic mode-resolved contributions. Conveniently, the SRME is applicable in this case, as the metric is symmetric and allows the reference and estimate to be exchanged. Therefore, to compare the derived quantities obtained with different displacement amplitudes, we use a DFT-DFT SRME, defined as

$$\mathrm{SRME}\big[\{\mathcal{K}(\boldsymbol{q})_s\}_{\mathrm{DFT,DFT}}\big] = \frac{2}{N_c\mathcal{V}} \frac{\sum_{\boldsymbol{q}s} |\mathcal{K}_{\mathrm{DFT1}}(\boldsymbol{q})_s - \mathcal{K}_{\mathrm{DFT2}}(\boldsymbol{q})_s|}{\kappa_{\mathrm{DFT1}} + \kappa_{\mathrm{DFT2}}}, \tag{3}$$

where DFT1 and DFT2 represent two DFT calculations with different displacement amplitudes. We define the $\mathrm{SRME}\big[\{\mathcal{G}(\boldsymbol{q})_s\}_{\mathrm{DFT,DFT}}\big]$ similarly. In this way, we compute the SRME values between the mode-resolved thermal conductivity and Grüneisen parameter obtained with DFT using different displacement amplitudes. The resulting

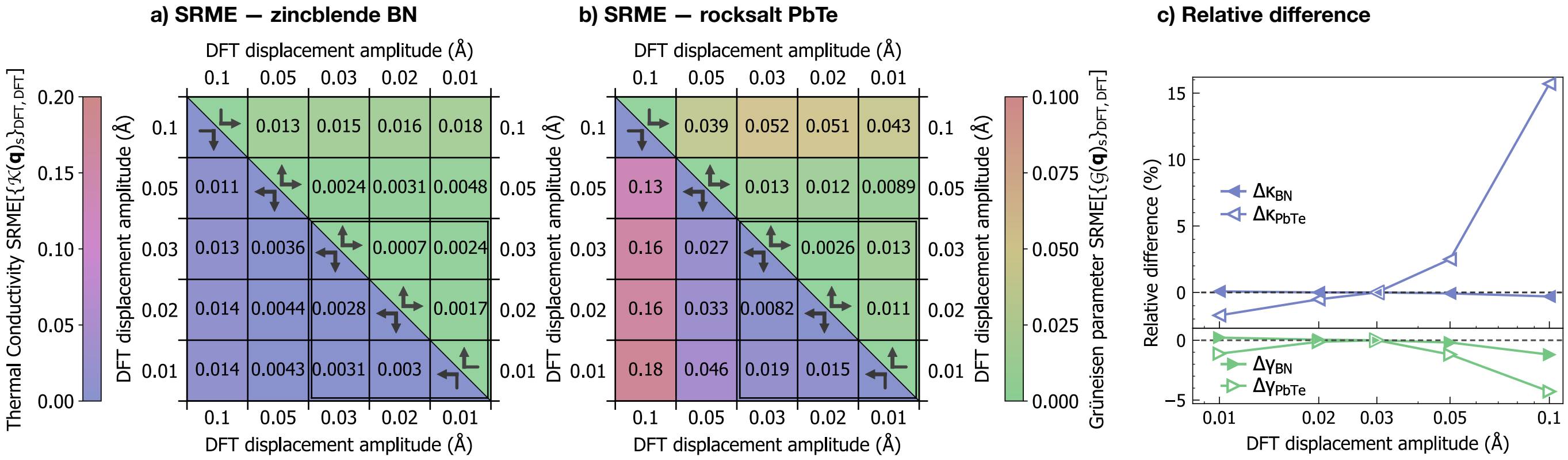


FIG. 2. **Effect of FD displacement amplitude on DFT conductivity and Grüneisen parameter** for microscopic SRME in BN (**a**) and PbTe (**b**), and corresponding macroscopic quantities (**c**). The DFT-DFT SRME of the phonon mode contribution to each thermomechanical quantity is calculated between each pair of DFT displacement amplitudes. Because the resulting matrices are symmetric with vanishing diagonals, we plot in the same matrix both the conductivity (lower triangle) and the Grüneisen parameter (upper triangle), using different color bars. **c** shows the relative deviation in the conductivity and Grüneisen parameter as a function of the FD amplitude compared to the results computed with the default 0.03Å.

SRME matrices of both quantities are symmetric with vanishing diagonals (the diagonal elements trivially correspond to comparing a DFT calculation with itself). Therefore, we plot the SRME matrices with the thermal-conductivity values in the lower triangular part and the Grüneisen-parameter values in the upper triangular part for BN and PbTe in Fig. 2**a,b**, respectively. Shared colormaps are used, with upper limits chosen to represent large deviations comparable to the performance of recent fMLPs[10,12]. Because the SRME for the mode-resolved Grüneisen parameter is consistently about a factor of two lower than that for the thermal conductivity, we visualize them using different colormaps. The changes in BN are minor, while they can be significant in PbTe, due to strong higher-order anharmonicity[13]. For PbTe, the deviations are large at 0.05Å and 0.1Å, while they are smaller between 0.01Å and 0.03Å, which we identify as the converged region and highlight with a double border to guide the eye.
The macroscopic quantities are also displayed in Fig. 2**c**, showing the relative difference in the total thermal conductivity and Grüneisen parameter at 300 K as a function of the displacement amplitudes. The same trends are observed: the results at 0.05Å and 0.1Å amplitudes in PbTe deviate significantly, whereas the lower amplitudes are converged, as in the case of BN. This confirms that the effect of displacement amplitude on the force constants and the derived thermomechanical quantities is material dependent, and that the displacement amplitude used here lies within the converged range.

The displacement regimes identified above apply to DFT calculations. When assessing the utility of fMLPs for computing these thermomechanical quantities, we need to consider noise and possible architecture-dependent inaccuracies in the PES described by the fMLP, which can amplify the influence of the FD amplitude on the thermomechanical properties compared to DFT. Since the DFT reference data used here are available with 0.03Å displacement amplitudes, and an ideal fMLP is expected to reproduce the DFT PES, benchmarks involving comparison between DFT and fMLP should use the original 0.03Å displacement amplitude of the reference datasets.

**Reviewer:** *3. I suggest the author hold out a small dataset to benchmark the models independently. So that these models are less likely to overfit on the benchmark.*

**Response:** We thank the Reviewer for the very useful suggestion. While this is outside of the focus of the current manuscript, in a future work we will retain a subset of the next released dataset to make it harder for models to overfit on these materials.
For the scope of the present work, to show that $\kappa_{SRME}$ and $\gamma_{SRME}$ computed on the 103 test materials in phononDB-PBE correlate with thermal conductivity and Grüneisen parameter predictions on other materials outside this 103-material database, we proceeded as follows. We studied rocksalt MgS, with Materials Project ID `mp-1315`, and BaLiAs, with Materials Project ID `mp-10616`, two high-symmetry crystal structures with small primitive cells that are not present in the 103-material database studied in the original version of the article.
Fig. 3 shows the SRME of conductivity and Grüneisen parameter for models from the same architectures discussed in this work, plotted against their $\kappa_{SRME}$ and $\gamma_{SRME}$ values on the 103 phononDB-PBE materials. The architectures

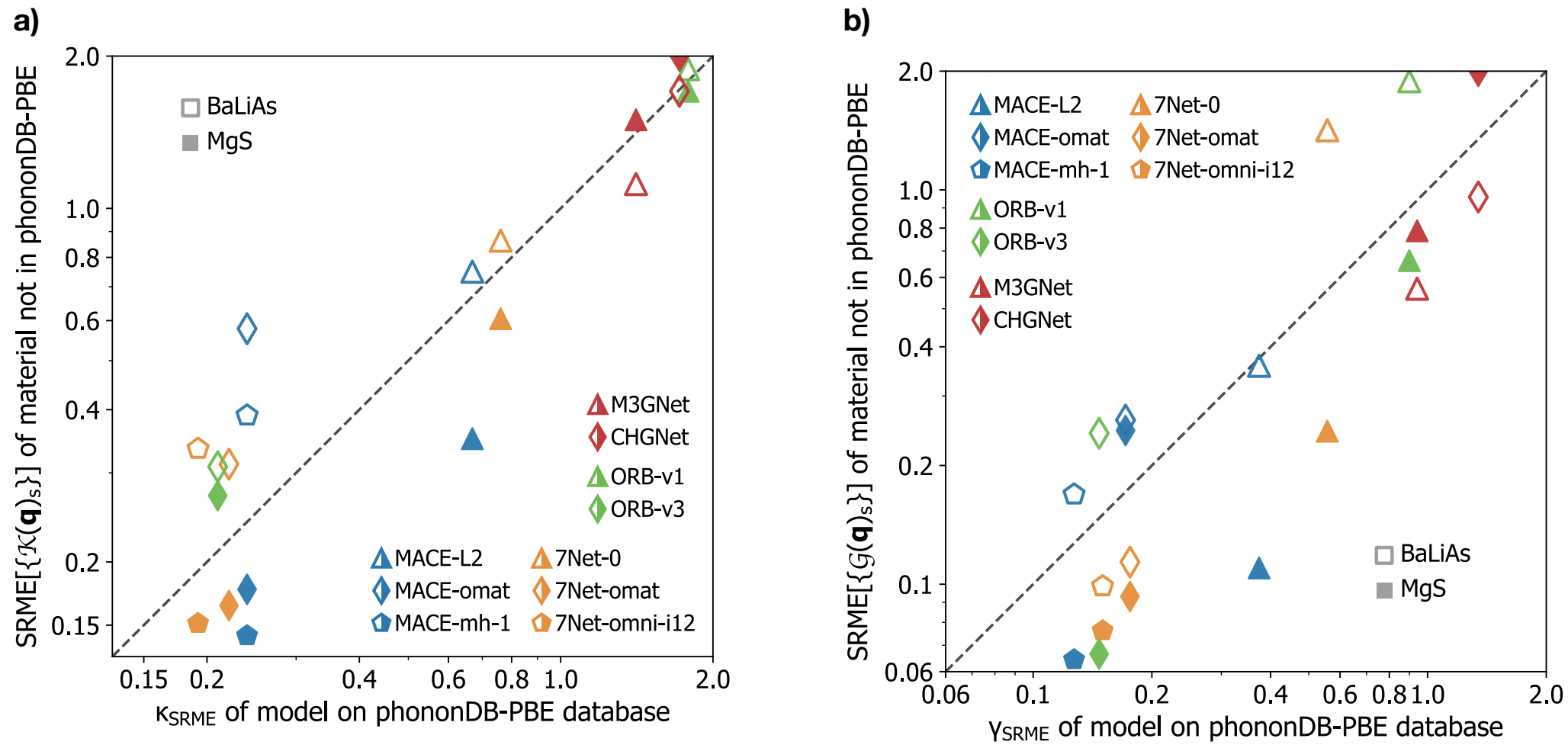


FIG. 3. **a) Thermal conductivity and b) Grüneisen parameter SRME against $\kappa_{SRME}$ and $\gamma_{SRME}$ values on the 103 phononDB-PBE materials** computed with models from the same architectures discussed in this work. The architectures are grouped by color, while the model generations are differentiated by marker style (MPTrj, OMAT, cross-domain). CHGNet and M3GNet are displayed under the same color for simplicity. Filled markers show the MgS results, and open markers represent BaLiAs.

are grouped by color, while the model generations are differentiated by marker style, corresponding to models trained on MPTrj, OMAT, or cross-domain datasets including molecules, surfaces and other systems. The exceptions are CHGNet and M3GNet, which are displayed under the same color for simplicity. Filled markers show the MgS results, while open markers represent BaLiAs. We see a clear correlation between the $\kappa_{SRME}/\gamma_{SRME}$ results on the phononDB-PBE 103-material dataset and the SRME results on these two additional materials, which suggests that these benchmark metrics are indicative of performance on additional materials.

Since the aim of this computation was to showcase additional materials that would be held out from the next dataset release, this analysis is only included in the peer review report and not in the manuscript. We thank the Reviewer again for the valuable comment, and in a future work we will retain a subset of the next released dataset to make it harder for models to overfit on these materials.

**Minor additional changes:** In the figures showing the fine-tuning dynamics (Fig. 5a,b; Fig. 11; Fig. 15), we changed the x-axis from the number of epochs to the number of gradient updates. In our setup, the number of gradient updates is the number of epochs multiplied by the number of fine-tuning frames. This representation allows a more consistent comparison between fine-tuning runs performed with datasets of different sizes.

**Reviewer:** *Again, I'd love to see this paper published. Nice work.*

**Response:** We are thankful for the Reviewer's positive evaluation.

---


[1] X. Gonze and C. Lee, Physical Review B **55**, 10355 (1997).
[2] B. A. A. Martin, A. M. Ganose, V. Kapil, T. Li, and K. T. Butler, General learning of the electric response of inorganic materials (2025), arXiv:2508.17870 [cond-mat.mtrl-sci].
[3] Z. Han, X. Yang, W. Li, T. Feng, and X. Ruan, Computer Physics Communications **270**, 108179 (2022).
[4] E. D. Lucente, N. Marzari, and M. Simoncelli, arXiv:2603.16753 [cond-mat.mtrl-sci] (2026).
[5] A. Castellano, J. Batista, and M. J. Verstraete, arXiv preprint arXiv:2502.03362 (2025).
[6] J. M. Ziman, *Electrons and phonons: the theory of transport phenomena in solids* (Oxford university press, 1960).
[7] A. Katre, A. Togo, I. Tanaka, and G. K. H. Madsen, Journal of Applied Physics **117**, 045102 (2015).
[8] M. Simoncelli, N. Marzari, and F. Mauri, Physical Review X **12**, 041011 (2022).
[9] A. J. H. McGaughey, L. Lindsay, H. Bao, T. Hamakawa, R. Juneja, S. Li, W. Li, R. Masuki, F. Meng, H. Meng, T. Pandey, C. Shao, J. Shiomi, T. Tadano, A. Togo, A. Wang, and X. Zhang, Journal of Applied Physics **138**, 135108 (2025).

[10] X. Fu, B. M. Wood, L. Barroso-Luque, D. S. Levine, M. Gao, M. Dzamba, and C. L. Zitnick, in *Forty-second International Conference on Machine Learning* (2025).
[11] Lattice thermal conductivity calculation with PFP (2025).
[12] F. Bigi, P. Pegolo, A. Mazitov, and M. Ceriotti, Pushing the limits of unconstrained machine-learned interatomic potentials (2026), arXiv:2601.16195 [physics.chem-ph].
[13] Y. Xia, Applied Physics Letters **113**, 073901 (2018).
[14] G. Petretto, S. Dwaraknath, H. P. C. Miranda, D. Winston, M. Giantomassi, M. J. van Setten, X. Gonze, K. A. Persson, G. Hautier, and G.-M. Rignanese, Scientific Data **5**, 180065 (2018).
[15] A. Togo, L. Chaput, and I. Tanaka, Phys. Rev. B **91**, 094306 (2015).
[16] A. Seko, A. Togo, H. Hayashi, K. Tsuda, L. Chaput, and I. Tanaka, Physical Review Letters **115**, 205901 (2015).
[17] W. Li, J. Carrete, N. A. Katcho, and N. Mingo, Computer Physics Communications **185**, 1747 (2014).
[18] A. Seko and A. Togo, Phys. Rev. B **110**, 214302 (2024).
[19] M. Al-Fahdi, R. Rurali, J. Hu, C. Wolverton, and M. Hu, npj Computational Materials (2025).
[20] H. Kraß, J. Huang, and S. M. Moosavi, npj Computational Materials **12** (2025).
[21] B. Rhodes, S. Vandenhaute, V. Šimkus, J. Gin, J. Godwin, T. Duignan, and M. Neumann, Orb-v3: atomistic simulation at scale (2025), arXiv:2504.06231 [cond-mat].
